# Supplementary figures and images for: Improved bacterial leaf blight disease resistance in the major elite Vietnamese rice cultivar TBR225 via editing of the OsSWEET14 promoter
Source: PLoS One. 2021 Sep 9;16(9):e0255470. doi: 10.1371/journal.pone.0255470 (PMC8428762; doi:10.1371/journal.pone.0255470)

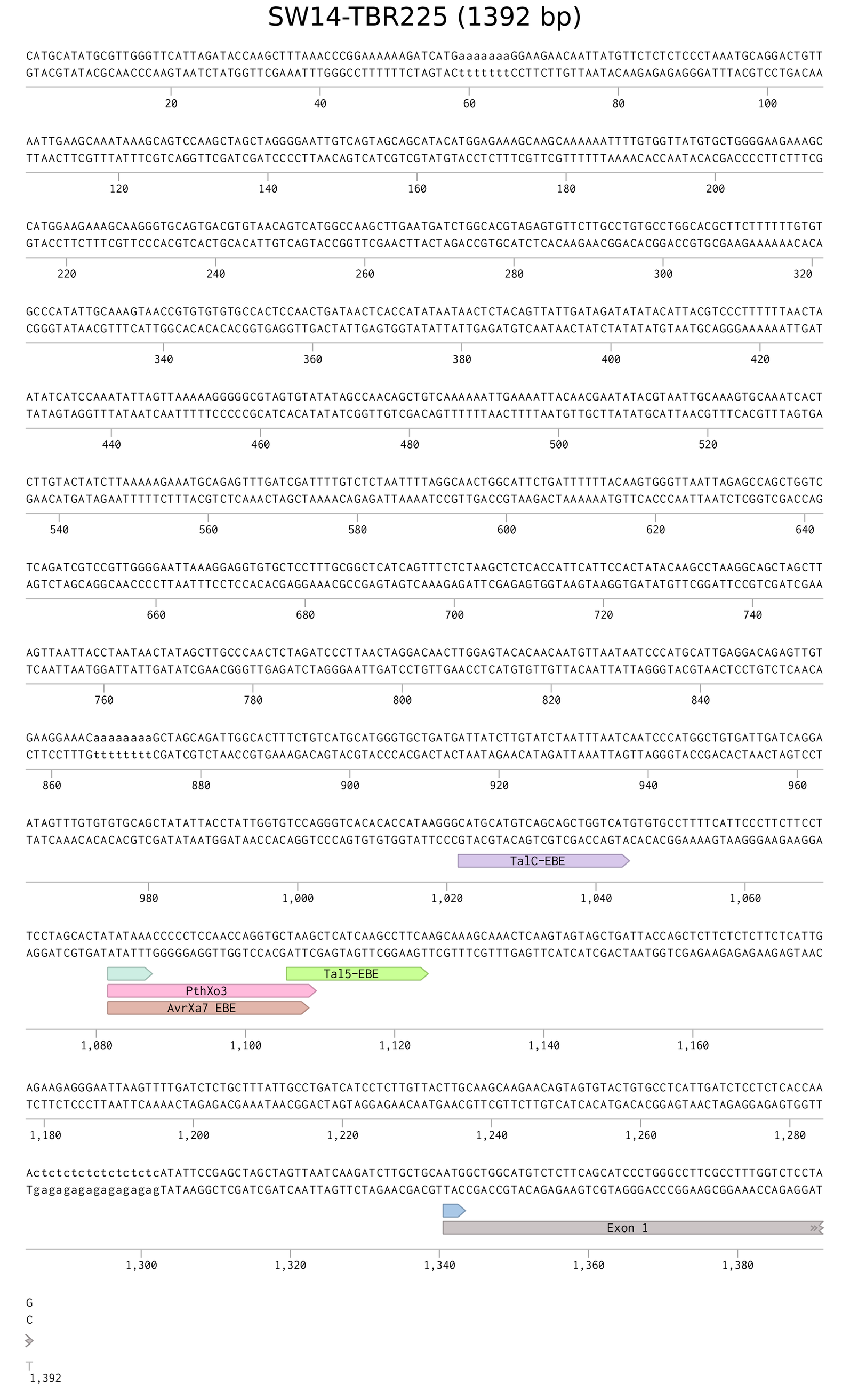

Supplement: S1 Fig — (TIF) [file pone.0255470.s001.tif]

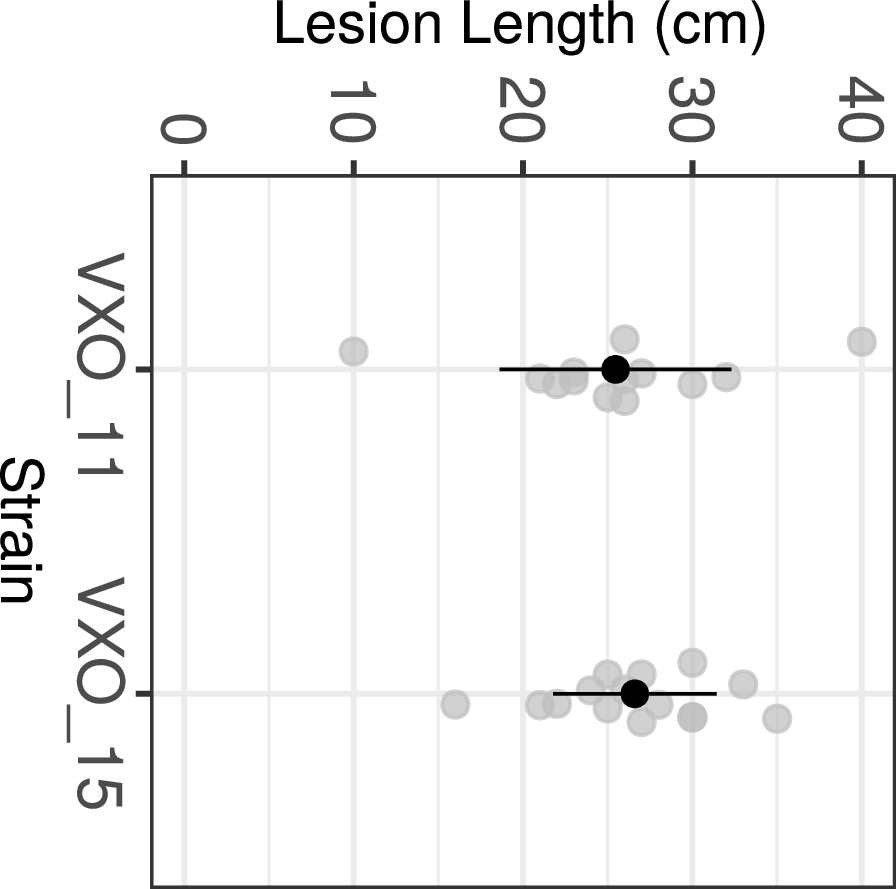

Supplement: S2 Fig — Grey points correspond to individual lesion length measurements while the black points indicate the calculated average value. The line range represents standard deviation. (TIF) [file pone.0255470.s002.tif]

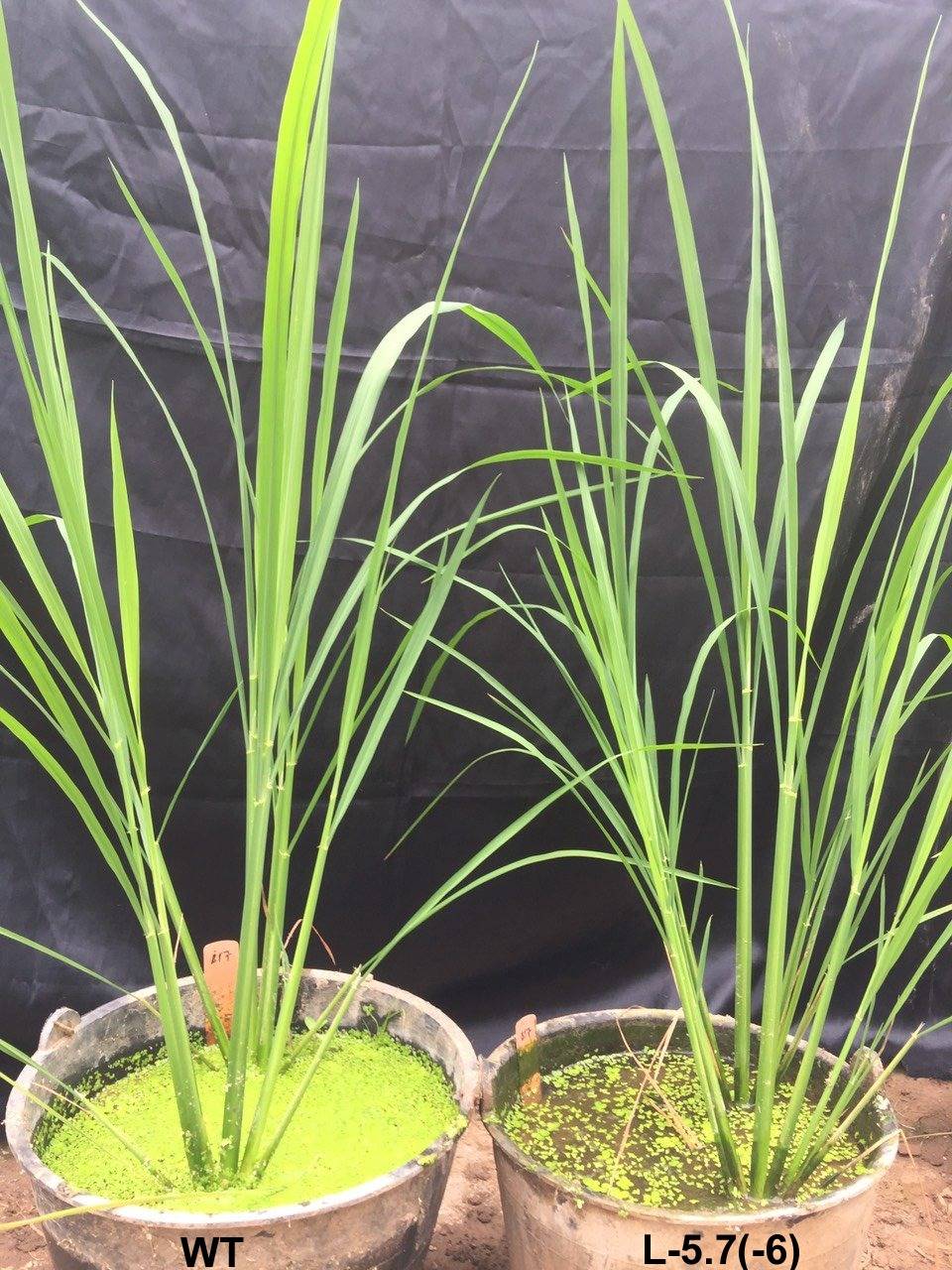

Supplement: S3 Fig — (TIF) [file pone.0255470.s003.tif]

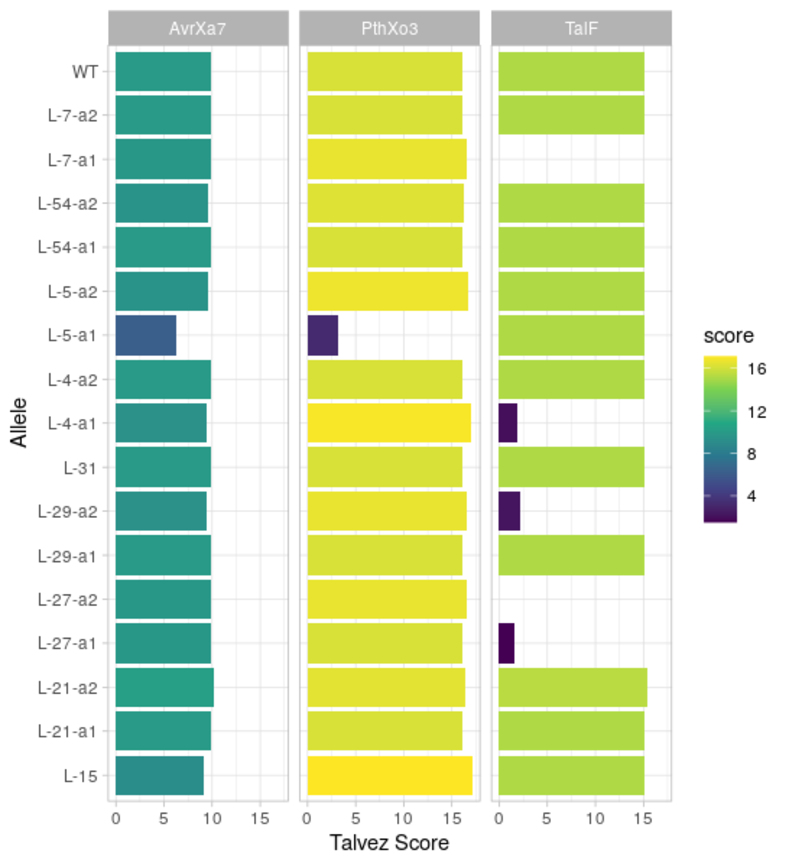

Supplement: S4 Fig — Score values are represented both by the length of the horizontal bar and a fill color scale. Higher Talvez prediction scores reflect a better match between a predicted EBE and the sequence of RVD of the query TALE. (TIF) [file pone.0255470.s004.tif]

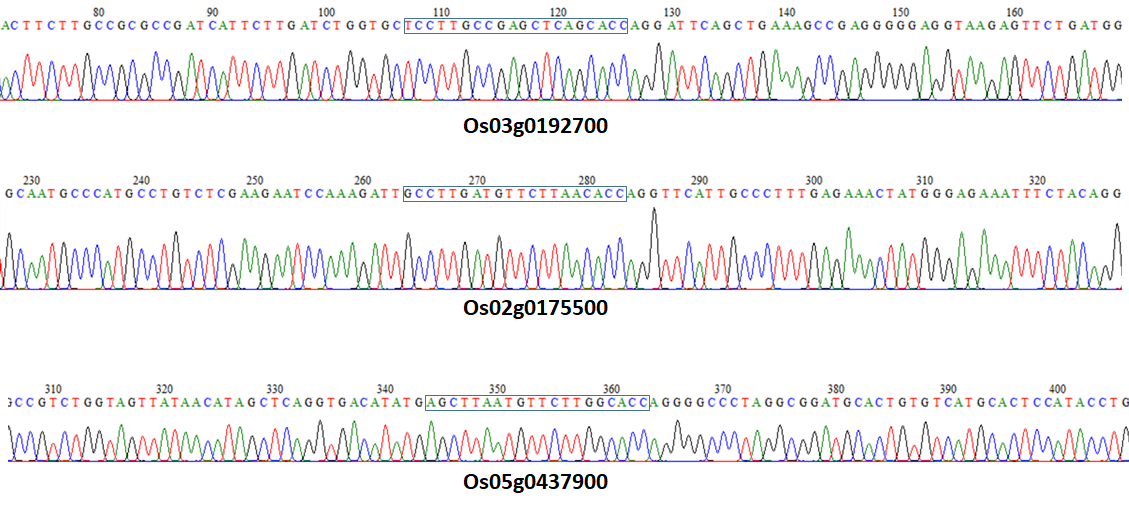

Supplement: S5 Fig — Potential unintended target sequences including the PAM are highlighted in boxes. They are all identical to the expected wild type Nipponbare sequences. (TIF) [file pone.0255470.s005.tif]
