# Supplementary material for: Improved bacterial leaf blight disease resistance in the major elite Vietnamese rice cultivar TBR225 via editing of the OsSWEET14 promoter
Source: PLoS One. 2021 Sep 9;16(9):e0255470. doi: 10.1371/journal.pone.0255470 (PMC8428762; doi:10.1371/journal.pone.0255470)
Supplement: S1 Table — (DOCX) [file pone.0255470.s006.docx]

| **Experiments** | **No. of selected samples** |
| --- | --- |
| Callus induction | 200 |
| Co-cultivation | 186 |
| Hygromycin selection | 41 |
| Shoot regeneration | 19 |
| PCR selection | 10 |
| *OsSWEET14* sequencing | 9 |
