# Supplementary material for: Improved bacterial leaf blight disease resistance in the major elite Vietnamese rice cultivar TBR225 via editing of the OsSWEET14 promoter
Source: PLoS One. 2021 Sep 9;16(9):e0255470. doi: 10.1371/journal.pone.0255470 (PMC8428762; doi:10.1371/journal.pone.0255470)
Supplement: S2 Table — (DOCX) [file pone.0255470.s007.docx]

| **Coordinates** | **MM** | **Target_seq** | **PAM** | **distance** | | **gene name** | **gene id** | **Primers for PCR amplification** | **Size of amplicon**  **(bp)** |
| --- | --- | --- | --- | --- | --- | --- | --- | --- | --- |
| [7:11741998-11742020](http://plants.ensembl.org/oryza_sativa/Location/View?r=7:11741998-11742020) | 4 | G**CGAC**GATGA[GCTTAGCACC] | AGG | 990 | - |  | [Os07g0298900](http://plants.ensembl.org/oryza_sativa/Gene/Summary?g=Os07g0298900) |  |  |
| [8:1692769-1692791](http://plants.ensembl.org/oryza_sativa/Location/View?r=8:1692769-1692791) | 4 | G**AT**TTGAT**TG**[GCTTAGCACC] | GGG | 3965 | - |  | [Os08g0130100](http://plants.ensembl.org/oryza_sativa/Gene/Summary?g=Os08g0130100) |  |  |
| [1:33530280-33530302](http://plants.ensembl.org/oryza_sativa/Location/View?r=1:33530280-33530302) | 4 | **A**G**A**TTGATG**G**[G**T**TTAGCACC] | AGG | 1467 | - | Intron_gpII | [ENSRNA049471700](http://plants.ensembl.org/oryza_sativa/Gene/Summary?g=ENSRNA049471700) |  |  |
| [4:8187721-8187743](http://plants.ensembl.org/oryza_sativa/Location/View?r=4:8187721-8187743) | 4 | G**CT**TTGAT**C**A[GC**C**TAGCACC] | AGG | 20942 | - |  | [Os04g0222250](http://plants.ensembl.org/oryza_sativa/Gene/Summary?g=Os04g0222250) |  |  |
| [11:24024507-24024529](http://plants.ensembl.org/oryza_sativa/Location/View?r=11:24024507-24024529) | 5 | G**CTC**TGAT**C**A[GC**C**TAGCACC] | AGG | 7324 | - |  | [Os11g0617532](http://plants.ensembl.org/oryza_sativa/Gene/Summary?g=Os11g0617532) |  |  |
| [10:7750146-7750168](http://plants.ensembl.org/oryza_sativa/Location/View?r=10:7750146-7750168) | 5 | G**CTC**TGAT**C**A[GC**C**TAGCACC] | AGG | 9408 | - |  | [Os10g0208900](http://plants.ensembl.org/oryza_sativa/Gene/Summary?g=Os10g0208900) |  |  |
| [4:801363-801385](http://plants.ensembl.org/oryza_sativa/Location/View?r=4:801363-801385) | 4 | G**C**CTTG**G**T**C**A[GCT**C**AGCACC] | GGG | 2053 | - |  | [Os04g0113000](http://plants.ensembl.org/oryza_sativa/Gene/Summary?g=Os04g0113000) |  |  |
| [3:4826386-4826408](http://plants.ensembl.org/oryza_sativa/Location/View?r=3:4826386-4826408) | 5 | **TC**CTTG**CC**GA[GCT**C**AGCACC] | AGG | 0 | E |  | [Os03g0192700](http://plants.ensembl.org/oryza_sativa/Gene/Summary?g=Os03g0192700) | F: TGCCGTATGTTGGAGACAG  R: AGCTCAGGATGGTAGCCACT | 338 |
| [1:35763341-35763363](http://plants.ensembl.org/oryza_sativa/Location/View?r=1:35763341-35763363) | 5 | G**TT**TTG**G**TGA[G**G**T**A**AGCACC] | CGG | 370 | - |  | [Os01g0834600](http://plants.ensembl.org/oryza_sativa/Gene/Summary?g=Os01g0834600) |  |  |
| [8:16276218-16276240](http://plants.ensembl.org/oryza_sativa/Location/View?r=8:16276218-16276240) | 4 | GG**T**TTGAT**C**A[GC**CC**AGCACC] | TGG | 8711 | - |  | [Os08g0356001](http://plants.ensembl.org/oryza_sativa/Gene/Summary?g=Os08g0356001) |  |  |
| [1:26275768-26275790](http://plants.ensembl.org/oryza_sativa/Location/View?r=1:26275768-26275790) | 5 | GGC**CA**GA**G**GA[**A**CTT**G**GCACC] | TGG | 1584 | - |  | [Os01g0650200](http://plants.ensembl.org/oryza_sativa/Gene/Summary?g=Os01g0650200) |  |  |
| [10:14248355-14248377](http://plants.ensembl.org/oryza_sativa/Location/View?r=10:14248355-14248377) | 5 | G**CT**TTGAT**C**A[GC**CC**AGCACC] | AGG | 7354 | - |  | [Os10g0410350](http://plants.ensembl.org/oryza_sativa/Gene/Summary?g=Os10g0410350) |  |  |
| [6:11384543-11384565](http://plants.ensembl.org/oryza_sativa/Location/View?r=6:11384543-11384565) | 5 | G**CT**TTGAT**C**A[GC**CC**AGCACC] | AGG | 51821 | - |  | [Os06g0302650](http://plants.ensembl.org/oryza_sativa/Gene/Summary?g=Os06g0302650) |  |  |
| [1:37435683-37435705](http://plants.ensembl.org/oryza_sativa/Location/View?r=1:37435683-37435705) | 5 | G**C**C**C**TGAT**A**A[GC**CC**AGCACC] | AGG | 4729 | - |  | [Os01g0865100](http://plants.ensembl.org/oryza_sativa/Gene/Summary?g=Os01g0865100) |  |  |
| [12:8623196-8623218](http://plants.ensembl.org/oryza_sativa/Location/View?r=12:8623196-8623218) | 5 | G**C**C**C**TGAT**C**A[GC**CC**AGCACC] | GGG | 35470 | - |  | [Os12g0254201](http://plants.ensembl.org/oryza_sativa/Gene/Summary?g=Os12g0254201) |  |  |
| [5:21461225-21461247](http://plants.ensembl.org/oryza_sativa/Location/View?r=5:21461225-21461247) | 5 | **A**GCTT**A**ATG**T**[**T**CTT**G**GCACC] | AGG | 0 | E |  | [Os05g0437900](http://plants.ensembl.org/oryza_sativa/Gene/Summary?g=Os05g0437900) | F: GCGGATAGTGGCAAGTTCCT  R: GTGGTGCTGCGAAATGACTC | 527 |
| [2:4130176-4130198](http://plants.ensembl.org/oryza_sativa/Location/View?r=2:4130176-4130198) | 4 | G**C**CTTGATG**T**[**T**CTTA**A**CACC] | AGG | 0 | E |  | [Os02g0175500](http://plants.ensembl.org/oryza_sativa/Gene/Summary?g=Os02g0175500) | F: CAGCAAGGACATGACAGCCA  R: GGGTATATCTGCGCCACACC | 347 |
| [2:31257196-31257218](http://plants.ensembl.org/oryza_sativa/Location/View?r=2:31257196-31257218) | 4 | **A**GCT**A**GAT**T**A[GCTTAGC**T**CC] | TGG | 1715 | - |  | [Os02g0745000](http://plants.ensembl.org/oryza_sativa/Gene/Summary?g=Os02g0745000) |  |  |
