# Supplementary material for: Improved bacterial leaf blight disease resistance in the major elite Vietnamese rice cultivar TBR225 via editing of the OsSWEET14 promoter
Source: PLoS One. 2021 Sep 9;16(9):e0255470. doi: 10.1371/journal.pone.0255470 (PMC8428762; doi:10.1371/journal.pone.0255470)
Supplement: S1 Raw images — (DOCX) [file pone.0255470.s008.docx]

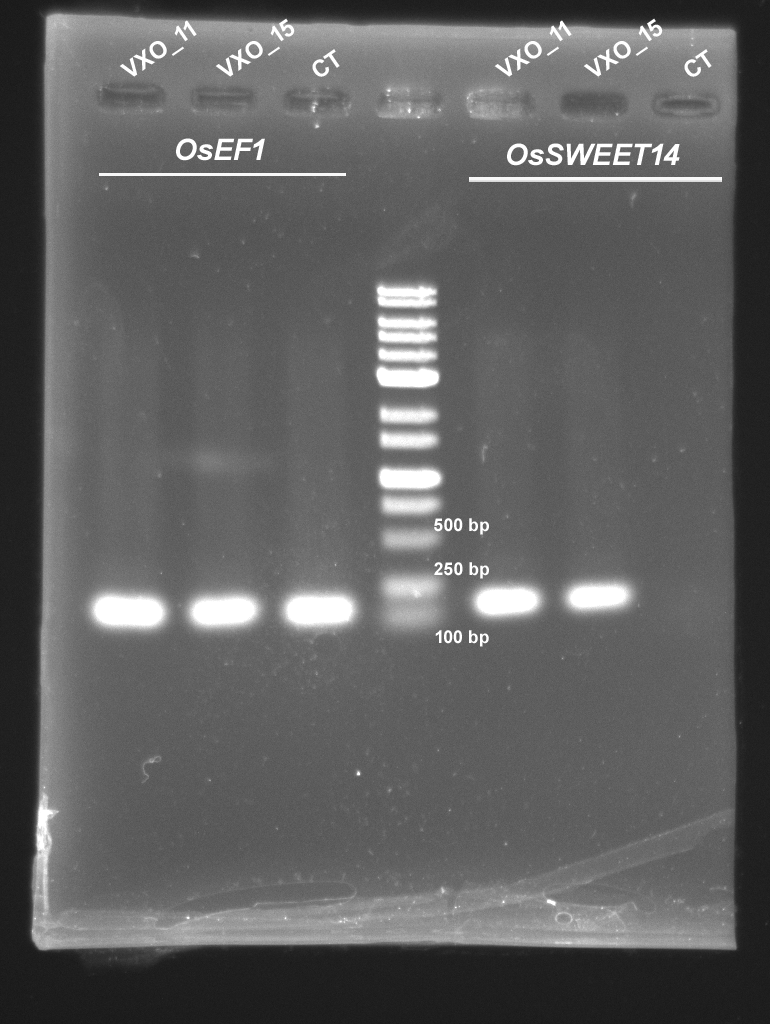


**(Raw_image for Figure2B):** Expression of *OsSWEET14* two day post-infiltration of TBR225 rice leaves with Vietnamese *Xoo* strains.(VXO_11) Plants were inoculated with VXO_11 strain; (VXO_15) Plants were inoculated with VXO_15 strain; (CT) Plants were inoculated with the water only. (*OsEF1*) RT-PCR with oligo (dT) primer followed by PCR with *OsEF1α*-specific primers. (*OsEF1*) RT-PCR with oligo (dT) primer followed by PCR with *OsSWEET14*-specific primers*.*
