# Supplementary material for: Improved bacterial leaf blight disease resistance in the major elite Vietnamese rice cultivar TBR225 via editing of the OsSWEET14 promoter
Source: PLoS One. 2021 Sep 9;16(9):e0255470. doi: 10.1371/journal.pone.0255470 (PMC8428762; doi:10.1371/journal.pone.0255470)
Supplement: S2 Raw images — (DOCX) [file pone.0255470.s009.docx]

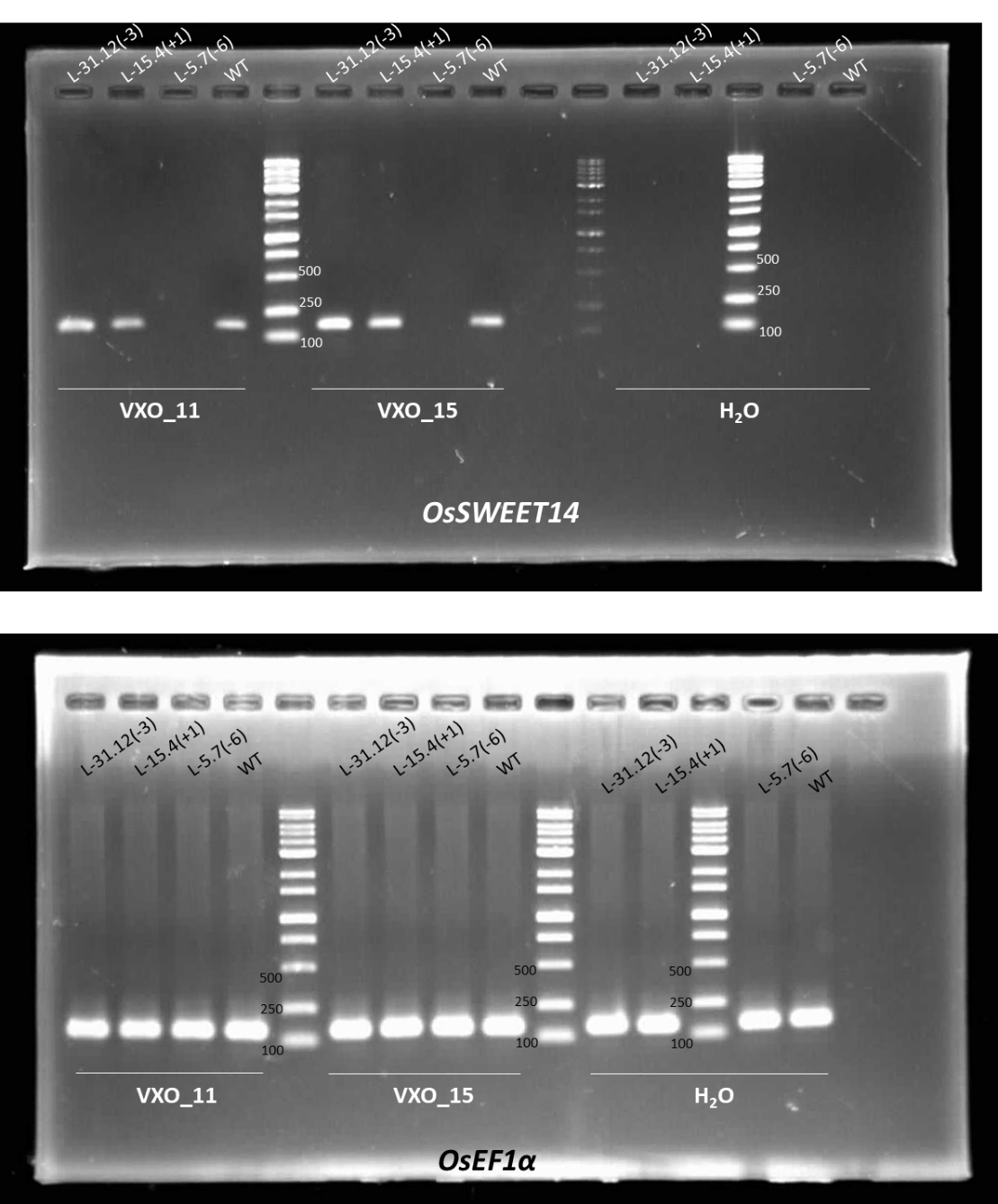


**(Raw_image for Figure 3C):** Expression of *OsSWEET14* and *OsEF1* two day post-infiltration of edited TBR225 lines L-31.12(-3), L-15.4(+1) and L-5.7(-6) with Vietnamese *Xoo* strains. (VXO_11) Plants were inoculated with VXO_11 strain; (VXO_15) Plants were inoculated with VXO_15 strain; (H_2_O) Plants were inoculated with the water only. (*OsEF1*) RT-PCR with oligo (dT) primer followed by PCR with *OsEF1α*-specific primers.
