# Supplementary material for: Improved bacterial leaf blight disease resistance in the major elite Vietnamese rice cultivar TBR225 via editing of the OsSWEET14 promoter
Source: PLoS One. 2021 Sep 9;16(9):e0255470. doi: 10.1371/journal.pone.0255470 (PMC8428762; doi:10.1371/journal.pone.0255470)
Supplement: Attachment — Submitted filename: Annotated file_PONE-D-20-33201_R2.pdf [file pone.0255470.s013.pdf]

PLOS ONE

Improved bacterial leaf blight disease resistance in the major elite Vietnamese rice cultivar TBR225 via editing of the OsSWEET14 promoter

--Manuscript Draft--

**Manuscript Number:**	PONE-D-20-33201R2
**Article Type:**	Research Article
**Full Title:**	Improved bacterial leaf blight disease resistance in the major elite Vietnamese rice cultivar TBR225 via editing of the OsSWEET14 promoter
**Short Title:**	Improved bacterial leaf blight disease resistance in Vietnamese rice cultivar TBR225
**Corresponding Author:**	Hoi Xuan Pham, Ph.D.<br>Institute of Agricultural Genetics<br>Hanoi, VIET NAM
**Keywords:**	Bacterial leaf blight; CRISPR/Cas9; Xanthomonas oryzae pv. oryzae; OsSWEET14; TBR225; transgene-free plants.
**Abstract:**	TBR225 is one of the most popular commercial rice varieties in Northern Vietnam. However, this variety is very susceptible to bacterial leaf blight (BLB), a disease caused by Xanthomonas oryzae pv. oryzae (Xoo) which inflicts important yield losses. OsSWEET14 belongs to the SWEET gene family that encodes sugar transporters. Together with other Clade III members, it behaves as a susceptibility (S) gene whose induction by Asian Xoo Transcription-Activator-Like Effectors (TALEs) is absolutely necessary for disease. In this study, we sought to introduce BLB resistance in the TBR225 elite variety. First, two Vietnamese Xoo strains were shown to up-regulate OsSWEET14 upon TBR225 infection. To investigate if this induction is connected with disease susceptibility, nine TBR225 mutant lines with mutations in the AvrXa7, PthXo3 or TalF TALEs DNA target sequences of the OsSWEET14 promoter were obtained using the CRISPR/Cas9 editing system. Genotyping analysis of T 0 and T 1 individuals showed that mutations were stably inherited. None of the examined agronomic traits of three transgene-free T2 edited lines were significantly different from those of wild-type TBR225. Importantly, one of these T 2 lines, harboring the largest homozygous 6-bp deletion, displayed decreased OsSWEET14 expression as well as a significantly reduced susceptibility to a Vietnamese Xoo strains and complete resistance to the other one. Our finding indicated that CRISPR/Cas9 is a useful and effective approach to improve BLB resistance of commercial elite rice varieties.
**Order of Authors:**	Phuong Duy Nguyen
	Dai Lan Tran
	Hang Thu Pham
	Phung Thi Thu Huong
	Ha Thanh Nguyen
	Ngoc Phuong Pham
	Florence Auguy
	Huong Thi Thu Bui
	Bao Manh Tran
	Sebastien Cunnac
	Hoi Xuan Pham, Ph.D.
**Response to Reviewers:**	We would like to thank you for your time and efforts handling and assessing our work. We hope our modifications of the initial manuscript address the concerns raised by the journal and reviewers.
**Additional Information:**	

Powered by Editorial Manager® and ProduXion Manager® from Aries Systems Corporation

Question	Response
**Financial Disclosure**<br><br>Enter a financial disclosure statement that describes the sources of funding for the work included in this submission. Review the submission guidelines for detailed requirements. View published research articles from *PLOS ONE* for specific examples.<br><br>This statement is required for submission and **will appear in the published article** if the submission is accepted. Please make sure it is accurate.<br><br>**Unfunded studies**<br>Enter: *The author(s) received no specific funding for this work.*<br><br>**Funded studies**<br>Enter a statement with the following details:<br>• Initials of the authors who received each award<br>• Grant numbers awarded to each author<br>• The full name of each funder<br>• URL of each funder website<br>• Did the sponsors or funders play any role in the study design, data collection and analysis, decision to publish, or preparation of the manuscript?<br>• **NO** - Include this sentence at the end of your statement: *The funders had no role in study design, data collection and analysis, decision to publish, or preparation of the manuscript.*<br>• **YES** - Specify the role(s) played.<br><br>* typeset	This work was supported by the National Technology Innovation Program of Vietnam (Grant No. ĐM.36.DN/18) funded by the Vietnam Ministry of Science and Technology (https://most.gov.vn/vn/pages/Trangchu.aspx) and ThaiBinh Seed Corporation (https://thaibinhseed.com.vn/trang-chu.aspx?lang=en-US).
**Competing Interests**<br><br>Use the instructions below to enter a competing interest statement for this submission. On behalf of all authors, disclose any competing interests that could be perceived to bias this work—acknowledging all financial support and any other relevant financial or non-financial competing interests.<br><br>This statement **will appear in the published article** if the submission is	The authors have declared that no competing interests exist

Powered by Editorial Manager® and ProduXion Manager® from Aries Systems Corporation

accepted. Please make sure it is accurate. View published research articles from *PLOS ONE* for specific examples.<br><br>**NO authors have competing interests**<br><br>Enter: *The authors have declared that no competing interests exist.*<br><br>**Authors with competing interests**<br><br>Enter competing interest details beginning with this statement:<br><br>*I have read the journal's policy and the authors of this manuscript have the following competing interests: [insert competing interests here]*<br><br>* typeset	
**Ethics Statement**<br><br>Enter an ethics statement for this submission. This statement is required if the study involved:<br><br>• Human participants<br>• Human specimens or tissue<br>• Vertebrate animals or cephalopods<br>• Vertebrate embryos or tissues<br>• Field research<br><br>Write "N/A" if the submission does not require an ethics statement.<br><br>General guidance is provided below. Consult the submission guidelines for detailed instructions. **Make sure that all information entered here is included in the Methods section of the manuscript.**	N/A

Powered by Editorial Manager® and ProduXion Manager® from Aries Systems Corporation

**Format for specific study types**<br><br>**Human Subject Research (involving human participants and/or tissue)**<br>• Give the name of the institutional review board or ethics committee that approved the study<br>• Include the approval number and/or a statement indicating approval of this research<br>• Indicate the form of consent obtained (written/oral) or the reason that consent was not obtained (e.g. the data were analyzed anonymously)<br><br>**Animal Research (involving vertebrate animals, embryos or tissues)**<br>• Provide the name of the Institutional Animal Care and Use Committee (IACUC) or other relevant ethics board that reviewed the study protocol, and indicate whether they approved this research or granted a formal waiver of ethical approval<br>• Include an approval number if one was obtained<br>• If the study involved *non-human primates*, add *additional details* about animal welfare and steps taken to ameliorate suffering<br>• If anesthesia, euthanasia, or any kind of animal sacrifice is part of the study, include briefly which substances and/or methods were applied<br><br>**Field Research**<br><br>Include the following details if this study involves the collection of plant, animal, or other materials from a natural setting:<br>• Field permit number<br>• Name of the institution or relevant body that granted permission	
**Data Availability**<br><br>Authors are required to make all data underlying the findings described fully available, without restriction, and from the time of publication. PLOS allows rare exceptions to address legal and ethical concerns. See the PLOS Data Policy and FAQ for detailed information.	Yes - all data are fully available without restriction

A Data Availability Statement describing where the data can be found is required at submission. Your answers to this question constitute the Data Availability Statement and **will be published in the article**, if accepted.<br><br>**Important:** Stating 'data available on request from the author' is not sufficient. If your data are only available upon request, select 'No' for the first question and explain your exceptional situation in the text box.<br><br>Do the authors confirm that all data underlying the findings described in their manuscript are fully available without restriction?	
**Describe where the data may be found in full sentences. If you are copying our sample text, replace any instances of XXX with the appropriate details.**<br><br>• If the data are **held or will be held in a public repository**, include URLs, accession numbers or DOIs. If this information will only be available after acceptance, indicate this by ticking the box below. For example: *All XXX files are available from the XXX database (accession number(s) XXX, XXX.).*<br>• If the data are all contained **within the manuscript and/or Supporting Information files**, enter the following: *All relevant data are within the manuscript and its Supporting Information files.*<br>• If neither of these applies but you are able to provide **details of access elsewhere**, with or without limitations, please do so. For example:<br><br>*Data cannot be shared publicly because of [XXX]. Data are available from the XXX Institutional Data Access / Ethics Committee (contact via XXX) for researchers who meet the criteria for access to confidential data.*<br><br>*The data underlying the results presented in the study are available from (include the name of the third party*	All relevant data are within the manuscript and its Supporting Information files.

and contact information or URL).<br>• This text is appropriate if the data are owned by a third party and authors do not have permission to share the data.<br><br>* typeset	
Additional data availability information:	Tick here if the URLs/accession numbers/DOIs will be available only after acceptance of the manuscript for publication so that we can ensure their inclusion before publication.; Tick here if your circumstances are not covered by the questions above and you need the journal's help to make your data available.

Powered by Editorial Manager® and ProduXion Manager® from Aries Systems Corporation

Manuscript Click here to access/download;Manuscript;Revised manuscript.docx

Full title:

Improved bacterial leaf blight disease resistance in the major elite Vietnamese rice cultivar TBR225 via editing of the *OsSWEET14* promoter

Short title:

Improved bacterial leaf blight disease resistance in Vietnamese rice cultivar TBR225

Nguyen Duy Phuong[1¶], Tran Lan Dai[1,2¶], Pham Thu Hang[1], Phung Thi Thu Huong[1], Nguyen Thanh Ha[1], Pham Phuong Ngoc[1,#a], Florence Auguy[3], Bui Thi Thu Huong[4], Tran Manh Bao[5], Sebastien Cunnac[3], Pham Xuan Hoi[1*]

[1]*Department of Molecular Pathology, Institute of Agricultural Genetics, Vietnam Academy of Agricultural Sciences, Hanoi, Vietnam.*

[2]*Department of Applied Biology and Agriculture, Faculty of Natural Sciences, Quynhon University, Quynhon, Vietnam.*

[3]*PHIM Plant Health Institute, Univ Montpellier, IRD, CIRAD, INRAE, Institut Agro, Montpellier, France.*

[4]*Vietnam National University of Agriculture, Hanoi, Vietnam.*

[5]*ThaiBinh Seed Corporation, Thaibinh, Vietnam.*

* Corresponding author: xuanhoi.pham@gmail.com

[¶] These authors contributed equally to this work.

[#a] Current address: Faculty of Biology, Hanoi University of Sciences, Hanoi, Vietnam.

Abstract

TBR225 is one of the most popular commercial rice varieties in Northern Vietnam. However, this variety is very susceptible to bacterial leaf blight (BLB), a disease caused by *Xanthomonas oryzae* pv. *oryzae* (*Xoo*) which inflicts important yield losses. *OsSWEET14* belongs to the *SWEET* gene family that encodes sugar transporters. Together with other Clade III members, it behaves as a susceptibility (*S*) gene whose induction by Asian *Xoo* Transcription-Activator-Like Effectors (TALEs) is absolutely necessary for disease. In this study, we sought to introduce BLB resistance in the TBR225 elite variety. First, two Vietnamese *Xoo* strains were shown to up-regulate *OsSWEET14* upon TBR225 infection. To investigate if this induction is connected with disease susceptibility, nine TBR225 mutant lines with mutations in the AvrXa7, PthXo3 or TalF TALEs DNA target sequences of the *OsSWEET14* promoter were obtained using the CRISPR/Cas9 editing system. Genotyping analysis of T_0 and T_1 individuals showed that mutations were stably inherited. None of the examined agronomic traits of three transgene-free T2 edited lines were significantly different from those of wild-type TBR225. Importantly, one of these T_2 lines, harboring the largest homozygous 6-bp deletion, displayed decreased *OsSWEET14* expression as well as a significantly reduced susceptibility to a Vietnamese *Xoo* strains and complete resistance to the other one. Our finding indicated that CRISPR/Cas9 is a useful and effective approach to improve BLB resistance of commercial elite rice varieties.

Keywords**: **Bacterial leaf blight; CRISPR/Cas9; Xanthomonas oryzae **pv.** ***oryzae; OsSWEET14; TBR225; transgene-free plants.***

Introduction

Bacterial leaf blight (BLB) caused by *Xanthomonas oryzae* pv. *oryzae* (*Xoo*) is a major bacterial disease that causes 10%-20% annual reduction in rice production worldwide [1]. The use of improved rice varieties resistant to *Xoo* is probably the most efficient, economical and environmentally-friendly way to control BLB.

The virulence of *Xoo* depends on the transcriptional activation of specific host disease-susceptibility (*S*) genes by a subgroup of bacterial type III effectors, called transcription activator-like effectors (TALEs) [2]. Upon translocation into the plant cell, TALEs bind to specific host nuclear gene promoter sequences termed Effector-Binding Elements (EBEs) and induce target gene expression to the benefit of the pathogen. The central repetitive domain of TALEs is responsible for DNA target sequence binding. DNA binding involves recognition principles that have been largely deciphered and applied to the computational prediction of TALEs target DNA sequences [3,4]. This and earlier work has fostered the identification of TALEs transcriptional targets in the rice genome and ultimately, of rice BLB *S* genes [2].

All *Xoo* strains recurrently target *S* genes belonging to the *SWEET* gene family and coding for transmembrane sugar exporter proteins [3]. The over accumulation of SWEETs due to TALE induction is presumed to provide an additional ration of apoplastic carbohydrates for full bacterial pathogen multiplication and disease expression [5]. Although all five rice clade III *SWEET* genes can function as *S* genes for bacterial

blight, only three, namely *OsSWEET11*, *OsSWEET13* and *OsSWEET14*, are known to be targeted by several unrelated TALEs in nature [6–11]. *OsSWEET11* is activated by PthXo1 [6], *OsSWEET13* is targeted by different variants of PthXo2 [11,12], while *OsSWEET14* is a target of multiple TAL effectors, including AvrXa7, PthXo3, TalC and TalF [7–9].

Previous studies established that rice resistance to *Xoo* resulting from "TALE-unresponsive" alleles can be conferred by natural DNA polymorphisms or targeted editions in EBEs located in *OsSWEET* genes promoters of rice germplasm accessions or engineered rice varieties, respectively [6,13–16]. For example, early resistance engineering work has used TALENs to individually alter the AvrXa7, TalC or TalF EBEs in the *OsSWEET14* promoter and successfully obtained resistance to some Asian *Xoo* strains [13,15]. However, strains collected in Asian countries such as China, Japan, Phillippines, Taiwan, Thailand, India, Nepal or South Korea can express combinations of up to three major TALEs redundantly targeting clade III *OsSWEET* genes with either PthXo3 or AvrXa7 being occasionally associated with PthXo2 [11,17]. Broad BLB resistance engineering thus required multiplex *OsSWEET* promoters EBE editing using the CRISPR/Cas9 system [11,12].

The clustered regularly interspaced short palindromic repeats/CRISPR-associated protein-9 nuclease (CRISPR/Cas9) system is a simple and efficient gene-editing tool developed in the past few years. Moreover, the targeted mutations generated by CRISPR/Cas9 can be stably transmitted to the next generation. Thus, CRISPR/Cas9 has

become a routine tool in plant laboratories around the world to create various mutants for many applications, including the genetic improvement of crops [18].

BLB is a major rice disease which occurs in many rice cultivating areas of Vietnam [19,20]. Most Vietnamese commercial rice varieties, including TBR225, are susceptible to BLB, resulting in annual yield loss of about 15 – 30% on average [21]. A few studies have identified rice resistance genes effective against Vietnamese *Xoo* lineages [20,21]. However, no information is currently available on the nature of Vietnamese *Xoo* TALEs and their corresponding *S* genes. Despite the large number of mapped rice BLB resistance genes [22], there is a need for alternative breeding approaches that enable the rapid introduction of broad BLB resistance in elite varieties in order to cope with swift pathogen populations adaptive shifts in the fields [11,23].

Here, we report on the identification of *OsSWEET14* as a transcriptional target of Vietnamese *Xoo*. CRISPR/Cas9-mediated mutagenesis of the *OsSWEET14* promoter in TBR225, a major elite variety in rice production areas of North Vietnam is shown to confer BLB resistance without detectable yield penalty. This study found this quintessential *S* gene to be associated with the virulence of Vietnamese *Xoo* strains. This is an important step for the future design and implementation of broad-spectrum BLB-resistant in elite rice varieties using genome editing in Vietnam.

Materials and methods

Plant and pathogen materials

Rice cultivar TBR225 (*Oryza sativa* L. ssp. *indica*) were obtained from ThaiBinh Seed Cor. [24]. All edited and wild-type (WT) TBR225 plants were grown in a net-house under the following average conditions: 30°C for 14 h (light) and 25°C for 10 h (dark) with 80% humidity. The *Xoo* VXO_11 and VXO_15 strains used in this study were isolated from diseased leaves collected in Hanoi-Vietnam in 2013 and 2016, respectively. Bacteria were cultured as described in Zhou et al. (2015) [25].

Gene expression analysis

Gene expression analyses were carried out as described previously [26] by RT-PCR method. The rice leaves were infiltrated with the indicated bacterial strains and used for total RNA extraction 48 h post inoculation using the TRIzol reagent (Invitrogen, USA). One microgram of RNA was used for each RT-PCR with oligo (dT) primer followed by PCR with *OsSWEET14*-specific primers (forward 5′-ACTTGCAAGCAAGAACAGTAGT-3′ and reverse 5′-ATGTTGCCTAGGAGACCAAAGG-3′). An *Eppendorf Mastercycler ep Gradient S* was used for 35 PCR cycles. The *OsEF1α* gene was used as a constitutive control [15] using specific primers (forward 5′-GAAGTCTCATCCTACCTGAAGAAG-3′ and reverse 5′-GTCAAGAGCCTCAAGCAAGG-3′).

gRNA design

The *OsSWEET14* promoter (GenBank, accession number: AP014967.1) was amplified by PCR with forward primer 5’-TTGCGGCTCATCAGTTTCTC-3’ and reverse primer 5’-CTAGGAGACCAAAGGCGAAG-3’ from genomic DNA of TBR225 rice plants and ligated in pGEM-T Easy vector (Promega) for sequencing. The gRNA target sequence (Fig 1A) for editing the TBR225 *OsSWEET14* promoter was designed based on the sequence of the cloned TBR225 *OsSWEET14* promoter using a combination of two bioinformatics tools CRISPR-P v2.0 (http://crispr.hzau.edu.cn/CRISPR2/) and CCTop (https://crispr.cos.uni-heidelberg.de/). A gRNA sequence with high on-target and low off-target scores in both prediction tools was chosen for vector construction.

Fig 1. CRISPR/Cas9-induced *OsSWEET14* promoter modification in TBR225 rice. (A) A region of the *OsSWEET14* promoter containing four EBEs (TalC, PthXo3, AvrXa7 and TalF) and putative TATA box from TBR225. The target site (complementary to the guide RNA) is shown in the box, immediately following the protospacer adjacent motif (PAM). (B) T-DNA region of the CRISPR/Cas9-mediated genome editing construct carrying OsSWEET14-sgRNA (indicated by the black box). The expression of *Cas9* is driven by the maize *ubiquitin* promoter (P-Ubi); the expression of the OsSWEET14-sgRNA is driven by the rice *OsU6* promoter (P-OsU6a); the expression of *HPT* is driven by two *CaMV35S* promoters (P-2×35S); T-35S, T-Nos and TTTTTT: gene terminators; LB and RB: left and right border, respectively. (C) Alignment of the *OsSWEET14* promoter fragment in the nine T_0 transgenic TBR225 rice plants edited in the AvrXa7,

PthXo3 and TalF EBEs. The lines on top of the wild-type sequence represent the binding sites of AvrXa7, PthXo3 and TalF. The arrow indicates the expected cutting site of the Cas9 complex used in this study. The labels on the left indicate the name of examined mutant lines; (a1) and (a2) distinguish alleles in the same line. The numbers on the right indicate the type of mutation and the number of nucleotides involved; (+) and (-) indicate insertion and deletion, respectively.

Vector construction

The Cas9 rice expression vector (pUbi-Cas9) [27] and the sgRNA expression vector (pENTR-sgRNA) under the control of the *OsU6* promoter [28] were used to construct the pCas9/OsSWEET14-gRNA expression vector. The complementary oligonucleotides with appropriate 4-bp overhangs were synthesized by Macrogen (Korea). After heat denaturation, the complementary oligonucleotides (5′-gtgtGGTGCTAAGCTCATCAAGCC-3’ and 5′-aaacGGCTTGATGAGCTTAGCACC-3’) were first annealed to each other, phosphorylated, and ligated into the *Bsa*I-digested vector pENTR-sgRNA. The integrity of the inserted fragment was verified by sequencing. Subsequently, the sgRNA cassette was cloned into pUbi-Cas9 using the Gateway LR clonase (Life Technologies) (Fig 1B). The resulting construct was confirmed by Sanger sequencing of the insertion junctions.

Agrobacterium-mediated rice transformation

The pCas9-OsSWEET14-gRNA was electroporated into *Agrobacterium tumefaciens* EHA105 and the resulting strain was used to transform rice using the method described by Hiei et al. (1994) [29]. The presence of the transgene in the genome of T_0 hygromycin-resistant plants or segregating T_1 individuals was evaluated by PCR using 5′-ATGGCCCCAAAGAAGAAG-3′ and 5′- GCCTCGGCTGTCTCGCCA-3′ primers specific for *Cas9*. T1 individuals were analyzed by PCR using *Cas9*, *OsSWEET14-gRNA* (5′- GGATCATGAACCAACG-3′ and 5′- GAATTCGATATCAAGCTT-3′) and *HPT* (5’-AAACTGTGATGGACGACACCGT-3’ and 5′- GTGGCGATCCTGCAAGCTCC -3′) specific diagnostic primer pairs together with a positive control pair (5′-TTGCGGCTCATCAGTTTCTC-3′ and 5′- TGGATCAGATCAAAGGCAAC -3′) specific to the *OsSWEET14* promoter.

Bacterial blight inoculation

Rice cultivation and disease assays were done according to the methods of Blanvillain-Baufumé et al. (2017) [15]. Bacteria were cultured in PSA media (10 g/liter peptone, 10 g/liter sucrose, 1 g/liter glutamic acid, 15 g/liter Bacto Agar) at 28°C for two days [30] and inoculated at an optical density (OD_{600}) of 0.5 (infiltrations) or 0.4 (leaf clipping) in water. For lesion length measurements, at least three inoculated leaves per plant and three plants for each line were measured 14 days after inoculation (DAI), and scored as

follows: high resistance (lesion length < 8 cm), moderate resistance (lesion length 8-12 cm) and susceptibility (lesion length > 12 cm). For gene expression analyses, 4-cm leaf sections which were infiltrated with bacterial suspensions were collected at 48 h after inoculation for RNA extraction. Experiments included samples from three pooled biological replicate leaves. The plants inoculated with distilled water only were used as negative controls.

Analysis of *OsSWEET14* edited allele sequences

To determine the nature of the mutation at the target site, all transgenic T_0 or T_1 plants were analyzed by PCR using genomic DNA (50 ng) as a template and *OsSWEET14* specific primers (5′-TTGCGGCTCATCAGTTTCTC-3′ and 5′-TGGATCAGATCAAAGGCAAC -3′). The PCR products were directly sequenced using the Sanger method. The sequencing chromatograms were decoded using the Degenerate Sequence Decoding method [31] in order to identify the mutations.

Evaluation of major agronomic traits under net-house conditions

WT and selected mutant plants were planted under net-house conditions in a randomized pot design experiment. At maturity, five plants of each line were investigated for the following agronomic traits: growth duration, plant height, number of tillers per plant, number of grains per panicle, number of filled grains per panicle and yield (seed mass)

per plant. The experiment was repeated three times, so a total of fifteen plants were evaluated for each line.

Analysis of potential off-target editing

Off-target sequences were predicted with the CCTop tool (https://crispr.cos.uni-heidelberg.de) **against the** *OsSWEET14* promoter **sgRNA** and the rice Nipponbare genome with default parameters. A total of 18 potential off-target sequences were identified. Three of them were located in coding regions (Table S2). These regions were amplified by PCR using the specific primers listed in Table S2 and analyzed by sequencing.

Results

Vietnamese *Xoo* strains induce *OsSWEET14* during infection of the TBR225 rice variety

OsSWEET14/Os11N3 was previously identified as a susceptibility gene for *Xoo* strains relying on either of the AvrXa7, PthXo3, TalF (formerly Tal5) or TalC TALEs for infection of the rice cultivars Nipponbare and Kitaake [11]. Because *Xoo* strains tend to frequently target this gene, we first sequenced a region of the *OsSWEET14* promoter from rice cultivar TBR225 to examine if it also carries documented target EBEs. Based

on the Nipponbare genome sequence in database (AP014967.1), the region encompassing 1343 bp sequence upstream and 52 bp sequence downstream of the predicted transcription start site of *OsSWEET14* gene from TRB225 rice cultivar was PCR amplified and sequenced (S1 Fig). The promoter region including the putative TATA box (TATAAA) and the AvrXa7, PthXo3, TalF/Tal5 and TalC EBEs (Fig 1A), located 319 bp to 216 bp upstream of the ATG initiation codon, showed 100% identity to the Nipponbare sequence. This therefore implied that in principle, the TBR225 *OsSWEET14* promoter can be recognized by characterized major *Xoo* TALEs.

As illustrated by the representative experiment of Fig 2A, we also challenged TBR225 plants with two Vietnamese *Xoo* strains VXO_11 and VXO_15, both originating from the Hanoi province, using leaf clipping assays. We consistently obtained typical extended disease lesions 14 days after inoculation (25.5 cm and 26.6 cm average lesions length for VXO_11 and VXO_15, respectively in the experiment of S2 Fig), indicating that the TBR225 variety is susceptible to BLB.

To test if *OsSWEET14* is a potential direct virulence target of Vietnamese *Xoo* strains, we infiltrated TBR225 rice leaves with the two Vietnamese *Xoo* strains. Forty-eight hours post infiltration, TBR225 plants inoculated with VXO strains displayed a strong induction of *OsSWEET14* relative to water controls (Fig 2B). These results suggest that *OsSWEET14* is a transcriptional target of VXO strains and that it may act as a susceptibility gene in TBR225.

Fig 2. *OsSWEET14* is likely a susceptibility gene for Vietnamese *Xoo* strains in rice cultivar TBR225. (A) Representative images of the disease lesions obtained 14 days after leaf clipping inoculation of TBR225 rice leaves with Vietnamese *Xoo* strains VXO_11 and VXO_15 or with water (CT). The chevrons above the leaves indicate the maximum visible extent of lesions away from the inoculation point on the left (B). *OsSWEET14* expression pattern obtained by RT-PCR two day post-infiltration of TBR225 rice leaves with Vietnamese *Xoo* strains. CT Plants were inoculated with water only. The experiment was repeated three times.

CRISPR/Cas9 design for *OsSWEET14* promoter editing

Our main objective was to engineer resistance to BLB caused by Vietnamese *Xoo* strains. To this end, we subsequently sought to specifically modify the *OsSWEET14* promoter in TBR225 rice with CRISPR/Cas9-mediated editing. Previous work revealed that while African *Xoo* strains rely on TalC and occasionally, TalF, all Asian *Xoo* strains use either PthXo3- or AvrXa7-like TALEs to activate *OsSWEET14* [11]. Because the *talC* gene is currently exclusively found in African strains, we reasoned that it is unlikely that Vietnamese strains carry a *talC* copy. Thus, to maximize our chances to perturb all remaining documented EBEs, we selected a 20-bp nucleotide target site overlapping the PthXo3, AvrXa7 and TalF EBEs and having a predicted cut site located near the 3'-end of the AvrXa7 EBE (Fig 1A). The recombinant binary plasmid pCas9/OsSWEET14-gRNA for CRISPR/Cas9 mediated editing of *OsSWEET14* was transformed into the rice variety

TBR225 via *Agrobacterium*-mediated transformation (S1 Table). A total of nine TBR225 transformants were selected from 10 independent PCR-validated transgenic T_0 TBR225 plants to further investigate CRISPR/Cas9-targeted mutagenesis of the *OsSWEET14* promoter. In order to decipher the nature of the editing events in *OsSWEET14*, the promoter sequencing data of transgenic lines were analyzed using the Degenerate Sequence Decoding software [31]. All 9 T_0 transgenic plants harbored at least an edition event (Fig 1C): two were heterozygous mutant/wild type, two had homozygous mutations, and five had bi-allelic mutations. Regarding the type of mutations, 66.7% were nucleotide deletions, 11.1% of the mutations were nucleotide insertions and no substitution was detected (Table 1).

Table 1. Frequencies of mutant genotypes and target mutation types in T_0 transgenic plants.

Mutant genotype ratios[a] (%)			Mutation type ratios[b] (%)		
**Heterozygote**	**Homozygote**	**Bi-allelic**	**Deletion**	**Insertion**	**Substitution**
22.2 (2/9)	22.2 (2/9)	55.6 (5/9)	66.7 (12/18)	11.1 (2/18)	0 (0/18)

[a] (Number of on-target mutant genotype/total number of on-target mutant genotypes) x 100%.
[b] (Number of allele mutation type/number of all allele mutation types) x 100%.

Inheritance of CRISPR/Cas9-induced mutations in the T_1 generation

To assess the inheritance of the CRISPR/Cas9-induced *OsSWEET14* mutations in the next generation, all T_0 mutant transgenic plants (Fig 1C) were allowed to self-pollinate, and T_1 transgenic plants were randomly selected in the progeny of T_0 plants for sequencing and analysis of their edited site (Table 2). All T_1 individuals derived from T_0 plants previously genotyped as homozygous possessed the same allele as their parent, suggesting stable inheritance of the mutations to the next generation. Similarly, the T1 progeny of each of both bi-allelic and heterozygous mutation T_0 lines showed a segregation ratio which is consistent with Mendelian segregation ($\chi^2 < \chi^2_{0.05,\ 2} = 5.99$), indicating that the CRISPR/Cas9-induced mutations in T_0 plants were transmitted as expected to the next generation. Interestingly, no new mutant allele was detected in the T_1 generation of both heterozygous mutants L-21 and L-27, even though most of them still carried the transgene. Overall, consistent with previous similar studies, our results indicate that the CRISPR/Cas9-mediated mutations generated here are stably transmitted to the next generation in a Medelian fashion.

Table 2. Transmission of CRISPR/Cas9 editing events to the T_1 generation.

T_0 plant	Genotype	Allele(s)	No. of T_1 plants tested	Mutation inheritance in the T_1 generation		No. of T-DNA-free plants
				Alleles segregation	χ^2 (1:2:1)	
L-4	Bi-allelic	-5/-3	32	10 (-5), 18 (-5/-3), 4 (-3)	2,750	5 (2*)
L-5	Bi-allelic	-6/+1	44	9 (-6), 22 (-6/+1), 13 (+1)	0,727	10 (2*)
L-7	Bi-allelic	-4/-3	38	14 (-4), 17 (-4/-3), 7 (-3)	3,000	11 (4*)

L-15	Homozygote	+1	5	5 (+1)	-	1 (1*)
L-21	Heterozygote	-3	26	3 (-3), 13 (-3/wt), 10 (wt)	3,769	7 (1*)
L-27	Bi-allelic	-5/-4	7	1 (-5), 3(-5/-4), 3 (-4)	1,286	0
L-29	Heterozygote	-5	33	6 (-5), 19 (-5/wt), 8 (wt)	1,000	2 (0*)
L-31	Homozygote	-3	15	15 (-3)	-	5 (5*)
L-54	Bi-allelic	-3/-2	21	3 (-3), 12 (-3/-2), 6(-2)	1,286	3 (0*)

"+" and "-" indicate respectively, insertion and deletion, of the indicated number of nucleotides.
"w", wild type.
*Number of homozygous mutant plants without T-DNA.

Selection of transgene-free mutant TBR225 rice lines

To identify T-DNA free T_1 rice plants containing a mutation in EBEs of the *OsSWEET14* promoter, PCR analysis was carried out using primers specific to *Cas9*, sgRNA and *HPT* sequences (Table 2). A T1 individual was considered devoid of the transgene if the control amplification of the *OsSWEET14* promoter was successful and if none of the PCR reactions with independent primer pairs designed on the T-DNA produced a detectable diagnostic band. The results of this PCR screen show that the T-DNA could be segregated out in the progeny of most T_0 lines, with 88.9% of the T_0 lines generating T-DNA-free progeny. In total, 44 of 221 analyzed edited T_1 plants did not generate a specific amplicon from the T-DNA construct and 15 of them were homozygous mutant harboring the desired *OsSWEET14* modifications. Our results demonstrate that transgene-free, homozygous mutant individuals could be obtained in the segregating progeny of selfed T_0 individuals.

TBR225 *OsSWEET14* promoter editing confers resistance to Vietnamese *Xoo*

To characterize the BLB-resistance phenotype of the generated rice mutants, three T-DNA-free, homozygous TBR225 edited lines, namely, L-5.7(-6), L-31.12(-3) and L-15.4(+1) with *OsSWEET14* promoter alleles corresponding respectively to L-5-a1 (6bp deletion), L-31 (3bp deletion) and L-15 (1bp insertion) in Fig 1C, were established. Selected T_1 individuals were propagated to obtain T_2 seeds which were used to perform BLB susceptibility assays. Edited T_2 and WT TBR225 plants were inoculated by leaf-clipping with the VXO_11 and VXO_15 strains at the eight-week stage. The inoculated leaves of wild type TBR225 plants and of edited lines L-15.4(+1) and L-31.12(-3) developed long water-soaked lesions typical of BLB, ranging from 18.3 cm to 29.0 cm in length. In contrast, the edited line L-5.7(-6), harboring a longer 6-bp deletion at the target site, displayed high (1.2 cm average lesion length) and moderate (7.3 cm average lesion length) resistance to VXO_11 and VXO_15 strains, respectively (Fig 3). Means comparisons with a Tukey's HSD test further indicated that irrespective of the inoculated strain, the mean lesion lengths measured on the L-15.4(+1), L-31.12(-3) or wild type lines were not significantly different. In contrast, the mean lesion lengths recorded on the L-5.7(-6) mutant line were significantly different from those obtained on the wild type and the two other edited lines challenged with either of the Vietnamese strains (Fig 3B). Furthermore, our off-target editing analysis on line L-5.7(-6) did not reveal unintended

modifications of other annotated rice loci (Table S2 and Figure S5), indicating that the 6-bp deletion in the *OsSWEET14* promoter is probably responsible for this phenotype.

Consistent with disease assays and as shown in Figure 3C, whereas a semiquantitative RT-PCR signal for *OsSWEET14* expression was detected on the parental variety and the L-15.4(+1) and L-31.12(-3) edited lines following VXO_11 and VXO_15 infiltration, this amplicon was undetectable in the resistant L-5.7(-6) line.

In conclusion, this data shows that the 6-bp deletion in the AvrXa7/PthXo3 EBE reduces dramatically *OsSWEET14* expression following VXO strains inoculation and confers resistance to these strains. In contrast, shorter modifications on the 3'-end of this EBE are insufficient to perturb *OsSWEET14* expression after inoculation and do not confer detectable protection against the corresponding strains. Finally, while these results strongly support the view that *OsSWEET14* functions as a unique susceptibility gene in the interaction between strain VXO_11 and the TBR225 rice variety, the resistance to strain VXO_15 is not as dramatic and may suggest that other mechanisms partially counteract the effects of the AvrXa7/PthXo3 EBE 6-bp deletion in edited TBR225 plants.

Fig 3. BLB resistance assays for homozygous mutant rice lines L-5.7(-6), L-15.4(+1) and L-31.12(-3). (A) Leaves were photographed 14 days post-leaf clipping inoculation of *Xoo* strains VXO_11 and VXO_15; arrow heads indicate the end of the lesion. (B) Mean lesion lengths (bars) and standard deviations (error bars). Values were measured 14 days post-leaf clipping inoculation of two *Xoo* strains VXO_11 and VXO_15 and were

computed from at least three leaves from each of three plants. Asterisks indicate significant differences relative to wild type plants (Tukey's HSD test; $**P < 0.05$). The number in the parentheses following the line name indicates the type of mutation and the number of nucleotides involved. The letters above strain labels indicate susceptibility score (R - high resistance; M – moderate resistance; S - susceptibility). The experiment was repeated three times. (C) *OsSWEET14* expression pattern obtained by RT-PCR two day post-infiltration of genome edited homozygous mutant rice lines L-31.12(-3), L-15.4(+1) and L-5.7(-6) and parental TBR225 rice leaves with Vietnamese *Xoo* strains. This experiment was repeated two times with similar results.

TBR225 *OsSWEET14* promoter edited lines agronomic performances are undistinguishable from the parental variety

To determine if mutations in the *OsSWEET14* promoter affect agronomic traits of TRB225 rice plants, three independent homozygous mutant lines were analyzed by measuring their growth duration, plant height, number of tillers per plant, number of grains per panicle, number of filled grains per panicle, yield per plant and amylose content under net-house conditions (see picture of S3 Fig). ANOVA tests and Student's *t* tests showed that the mutant lines displayed no significant difference to TBR225, in terms of the examined agronomic traits, under our net-house conditions (Table 3). These

results suggest that the tested CRISPR/Cas9-induced mutations in the *OsSWEET14* promoter did not negatively impact the main agronomic traits of TBR225.

Table 3. Agronomic traits evaluation of homozygous T_2 mutant lines.

Lines	Growth duration (day)	Plant height (cm)	No. of tillers per plant	No. of grains per panicle	No. of filled grains per panicle	Amylose content (%)
WT	108.4 ± 1.1^a	86.6 ± 3.2^a	5 ± 0.7^a	144.4 ± 4.9^a	125 ± 4.5^a	13.2 ± 0.38^a
L-5.7(-6)	108 ± 1.2^a	86.4 ± 4.3^a	5.2 ± 0.4^a	144.2 ± 4.4^a	123.4 ± 5.5^a	13.7 ± 0.35^a
L-15.4(+1)	107.8 ± 0.8^a	86.4 ± 5.0^a	4.8 ± 0.4^a	147.8 ± 5.1^a	121.8 ± 3.0^a	13.5 ± 0.41^a
L-31.12(-3)	108 ± 1.2^a	88.4 ± 4.3^a	5.4 ± 0.5^a	144.6 ± 5.3^a	124.2 ± 7.4^a	13.8 ± 0.21^a

Five plants per line were measured. Experiments were repeated three time.
Means followed by the same letter do not differ significantly ($P < 0.05$).

Discussion

Recently, the CRISPR/Cas9 system has emerged as a powerful tool for gene editing in many organisms including plants. Because of its specificity and efficiency, this system has been widely used to improve important agronomic traits of major crops such as rape, tomato, soybean, rice, wheat and maize [32]. Excluding easy-to-transform reference accessions such as Nipponbare and Kitaake that are widely used in the laboratory, the number of reports on the improvement of agriculturally relevant elite rice cultivars for pertinent traits using the CRISPR/Cas9 technology (see for example [33–36]) is gradually increasing but is still limited.

TBR225 [24], a major commercial rice variety cultivated in large areas of Northern Vietnam, has the advantages of early maturity, high and stable yield, as well as cooking quality. However, it is very susceptible to BLB. Here, the CRISPR/Cas9-mediated editing method was applied in order to rapidly improve the BLB resistance of TBR225 by modifying the AvrXa7, PthXo3 and TalF EBEs on the promoter of *OsSWEET14*. Of the three generated homozygous mutant lines tested for resistance, the one carrying the largest deletion at the target site (6 bp) showed a significantly improved resistance to infection with two *Xoo* strains VXO_11 and VXO_15. Therefore, using the major commercial rice variety TBR225 as an example, we illustrate the advantages of CRISPR/Cas9 tool for rice breeding.

In the present study, the frequency of individuals with CRISPR/Cas9-induced mutations in T_0 transgenic plants was 90%, which is similar to previous observation [28]. We obtained only two heterozygous mutant/wild type lines versus seven homozygous or bi-allelic mutant lines. This high frequency of mutated alleles is another proof that the CRISPR/Cas9 system is indeed an efficient tool for gene editing in plant. We also observed the stable transmission of edited alleles to subsequent generations. This is a common phenomenon that has been repeatedly documented for rice plants carrying CRISPR/Cas9-induced mutations [33,35]. In this study, we obtained only two types of induced mutations in T_0 plants: insertion (11.1%) and deletion (66.7%), but no substitution were observed. In some earlier studies, new mutations were continuously obtained in the T_1 offspring of heterozygous T_0 mutants because the Cas9 complex

remains active on edited targets until the seed or PAM regions cease to be functional [35,37,38]. In contrast, here, all the T_1 plants generated from both heterozygous lines L-21 and L-29, regardless of whether they had a CRISPR/Cas9 T-DNA transgene integrated in their genome, did not show any new mutation possibly because CRISPR/Cas9 T-DNA transgene was no longer functional. We could also readily obtain transgene-free plants from most of the T_1 segregation populations without any laborious crossing or backcrossing steps, which illustrates an advantage of the CRISPR/Cas9 technology compared to conventional breeding.

Clade III SWEET family proteins are involved in a number of biological processes such as seed and pollen development or pathogen susceptibility [39]. Their inactivation has previously been shown to cause pleiotropic and/or detrimental effects. For example, both *ossweet11* single and *ossweet11-ossweet15* double Kitaake rice mutants showed defects in endosperm development and filling [40]. In addition, RNA-mediated silencing of either *Os11N3/OsSWEET14* [7] or *Os8N3/OsSWEET11* [6] in BLB resistant Kitaake lines causes negative effects on seed production. In contrast, here, we show that T-DNA-free TBR225 plants harboring homozygous mutations generated with the CRISPR/Cas9 system in the AvrXa7/PthXo3 EBE of the *OsSWEET14* promoter exhibited enhanced *Xoo* resistance but did not show any significant difference in all examined agronomic traits compared to wild-type plants under net-house growth conditions. It is conceivable that limited modifications in promoter regions do not affect the normal expression of *SWEET* genes in contrast to KO or silenced lines. Our findings are consistent with the

previous work of Oliva et al. [11] who studied 30 combinations of EBE mutations in the *OsSWEET11*, *OsSWEET13* and *OsSWEET14* promoters of the IR64 or Ciherang-Sub1 varieties and detected only a single line with abnormal agronomic traits.

Some individual *Xoo* strains have evolved a set of distinct TALE effectors that collectively target several members of the clade III SWEET family. The presence of these redundant TALEs thereby trumps single "loss-of-tale-responsiveness" resistance alleles [11,12,17,41]. For example, Kitaake lines carrying TALEN-induced mutation in the *SWEET14* promoter [13,15] exhibit resistance to strains which depend exclusively on matching AvrXa7/PthXo3 for clade III *SWEET* family induction. Likewise, the natural *xa13* allele [42] or CRISPR/Cas9-induced mutation in the *SWEET11* promoter [11] exhibit resistance to strains such as PXO99 which depend exclusively on PthXo1, for virulence. However, the BLB resistance of the Kitaake lines harboring mutations in both AvrXa7/PthXo3 (*OsSWEET14*) and PthXo1 (*OsSWEET11*) EBEs was defeated by *Xoo* strains expressing simultaneously the AvrXa7/PthXo3 and PthXo2B TALEs [11]. Recently, the stacking of EBE-edited alleles in several *OsSWEET* promoters have overcome this limitation and was shown to achieve a broad spectrum of resistance to strains from most BLB-prone countries in Asia [11,12].

All of the three T_2 lines tested for BLB resistance were affected for the AvrXa7/PthXo3 EBE and conserved an otherwise wild type TalF EBE (Fig 1C). The homozygous mutant TBR225 line L-5.7(-6) carrying a 6-bp deletion in the AvrXa7/PthXo3 EBE exhibited a significantly enhanced resistance to two Vietnamese *Xoo* strains compared to WT

TBR225. The L-15.4(+1) and L-31.12(-3) lines that harbored more subtle alterations in the 3'-end of this EBE (a 1-bp insertion and a 3-bp deletion, respectively) in contrast remained susceptible to VXO strains. Our *OsSWEET14* expression analysis after Vietnamese Xoo strains inoculation (Fig 1C) suggests that these editing events did not alter the EBE sequence sufficiently to compromise promoter recognition by an AvrXa7/PthXo3-like Vietnamese TALE. With less than 2 cm average lesion length, the resistance of line L-5.7(-6) (6-bp deletion) to the VXO_11 strain is rather extreme (versus average lesion length of 20.1 cm on wild type plants). Moreover, in this line, *OsSWEET14* expression following bacterial inoculation is strongly reduced relative the parental line and the two other edited lines, which suggest that in this case, recognition by an AvrXa7/PthXo3-like Vietnamese TALE is abrogated. Consistent with *OsSWEET14* expression analysis and as shown in S4 Fig, the Talvez [43] target prediction scores for AvrXa7 and PthXo3 on the *OsSWEET14* promoter L-5-a1 allele sequence of line L-5.7(-6) are markedly lower than on the wild type promoter sequence. This is not the case however for the edited alleles carried by lines L-15.4(+1) and L-31.12(-3) (respectively L-15 and L-31 in S4 Fig) whose Talvez scores are identical or slightly lower than those of the wild type promoter sequence.

The magnitude of the effect of the 6-bp deletion allele on susceptibility to VXO_11 is comparable to the dramatic effect of previously characterized alterations of the same EBEs in the Kitaake background against the PXO86 strain that possesses a single TALE, AvrXa7, targeting *OsSWEET14* for clade III *OsSWEET* gene induction [15]. By analogy,

this suggests that *OsSWEET14* is also the only clade III *OsSWEETs* target of VXO_11 in the TBR225 background but, in order to confirm this hypothesis an examination of other clade III *OsSWEET* genes expression patterns in response to this strain would be required. The situation with the VXO_15 strain is not as straightforward to interpret and will require further investigations. Although the 6-bp deletion in the AvrXa7/PthXo3 EBE did provide an increased resistance to the edited plants, the VXO_15 strain caused intermediate disease severity (7.3 cm average lesion length on Fig 3). This incomplete resistance is unlikely to result from the partial but still productive recognition of subsequences of the altered EBE by a VXO_15 AvrXa7/PthXo3-like TALE because *OsSWEET14* expression is similarly decreased in response to either this strain or VXO_11 (Fig 3C). Alternatively, contrary to all Asian *Xoo* examined so far, but similar to African *Xoo* [15], VXO_15 may have the intrinsic potential to cause disease in the absence of clade III *OsSWEET* gene induction. More likely, analogous to other Asian strains, VXO_15 may encode alternative TALEs, such as PthXo2B or PthXo1 that compensate the loss of *OsSWEET14* induction by targeting other clade III *OsSWEET* genes. In this regard, long read genome sequencing will ultimately help describe TALEs variability in Vietnamese *Xoo* strains.

In conclusion, we showed that editing specific EBEs of *Xoo* TALEs via CRISPR/Cas9 tool is an efficient method for improving BLB resistance of elite rice varieties such as TBR225 without detectable yield penalties. This also uncovered the potential diversity of TALEs in Vietnamese *Xoo* population, which will thus require future investigations to

address the TALE repertoires of Vietnamese *Xoo* strains in order to generate broad-spectrum BLB-resistant rice varieties in Vietnam.

Authors' Contributions

Nguyen Duy Phuong and Tran Lan Dai are equal contributors

Conceptualization: Pham Xuan Hoi, Sebastien Cunnac, Nguyen Duy Phuong

Data Curation: Nguyen Duy Phuong

Formal Analysis: Nguyen Duy Phuong, Tran Lan Dai**,** Sebastien Cunnac, Pham Xuan Hoi

Funding Acquisition: Nguyen Duy Phuong, Pham Xuan Hoi, Tran Manh Bao

Investigation: Nguyen Duy Phuong, Tran Lan Dai, Pham Thu Hang, Phung Thi Thu Huong, Nguyen Thanh Ha, Pham Phuong Ngoc, Florence Auguy, Sebastien Cunnac

Methodology: Nguyen Duy Phuong**,** Tran Lan Dai, Pham Thu Hang, Sebastien Cunnac, Nguyen Thanh Ha, Pham Xuan Hoi

Project Administration: Pham Xuan Hoi

Resources: Pham Thu Hang, Florence Auguy, Nguyen Thanh Ha

Supervision: Sebastien Cunnac, Pham Xuan Hoi

Validation: Sebastien Cunnac, Pham Xuan Hoi

Writing – Original Draft Preparation: Nguyen Duy Phuong, Tran Lan Dai

Writing – review & editing: Sebastien Cunnac, Pham Xuan Hoi, Tran Manh Bao, Bui Thi Thu Huong

Acknowledgments

We are grateful to Msc. Pham Thi Van, Dr. Cao Le Quyen and Dr. Nguyen Van Cuu from the Institute of Agricultural Genetics for rice transformation experiments, Msc. Nguyen Thi Thu Ha from the Institute of Agricultural Genetics for managing the *Xoo* strains collection and Msc. Nguyen Thi Nhung from Thaibinh Seeds Cor. for kindly providing the rice accessions.

References

1. Zhang H, Wang S. Rice versus *Xanthomonas oryzae* pv. *oryzae*: A unique pathosystem. Curr Opin Plant Biol. 2013; 16(2):188-195. doi:10.1016/j.pbi.2013.02.008 PMID: 23466254

2. White FF, Potnis N, Jones JB, Koebnik R. The type III effectors of *Xanthomonas*. Mol Plant Pathol. 2009; 10(6):749-766. doi:10.1111/j.1364-3703.2009.00590.x PMID: 19849782

3. Boch J, Bonas U, Lahaye T. TAL effectors - pathogen strategies and plant resistance engineering. New Phytol. 2014; 204(4):823-832. doi:10.1111/nph.13015 PMID: 25539004

4. Bogdanove AJ, Schornack S, Lahaye T. TAL effectors: Finding plant genes for disease and defense. Curr Opin Plant Biol. 2010; 13(4):394-401. doi:10.1016/j.pbi.2010.04.010 PMID: 20570209

5. Bezrutczyk M, Yang J, Eom JS, Prior M, Sosso D, Hartwig T, Szurek B, Oliva R, Vera-Cruz C, White FF, Yang B, Frommer WB. Sugar flux and signaling in plant–microbe interactions. Plant J. 2018; 93(4):675-685. doi:10.1111/tpj.13775 PMID: 29160592

6. Yang B, Sugio A, White FF. *Os8N3* is a host disease-susceptibility gene for

bacterial blight of rice. Proc Natl Acad Sci U S A. 2006; 103(27):10503-10508. doi:10.1073/pnas.0604088103 PMID: 16798873

7. Antony G, Zhou J, Huang S, Li T, Liu B, White F, Yang B. Rice *xa13* recessive resistance to bacterial blight is defeated by induction of the disease susceptibility gene *Os-11N3*. Plant Cell. 2010; 22(11):3864-3876. doi:10.1105/tpc.110.078964 PMID: 21098734

8. Yu Y, Streubel J, Balzergue S, Champion A, Boch J, Koebnik R, Feng J, Verdier V, Szurek B. Colonization of rice leaf blades by an African strain of X*anthomonas oryzae* pv. *oryzae* depends on a new TAL effector that induces the rice nodulin-3 *Os11N3* gene. Mol Plant-Microbe Interact. 2011; 24(9):1102-1113. doi:10.1094/MPMI-11-10-0254 PMID: 21679014.

9. Streubel J, Pesce C, Hutin M, Koebnik R, Boch J, Szurek B. Five phylogenetically close rice *SWEET* genes confer TAL effector-mediated susceptibility to *Xanthomonas oryzae* pv. *oryzae*. New Phytol. 2013; 200(3):808-819. doi:10.1111/nph.12411 PMID: 23879865

10. Zhou J, Peng Z, Long J, Sosso D, Liu B, Eom JS, Huang S, Liu S, Vera Cruz C, Frommer WB, White FF, Yang B. Gene targeting by the TAL effector PthXo2 reveals cryptic resistance gene for bacterial blight of rice. Plant J. 2015; 82(4):632-643. doi:10.1111/tpj.12838 PMID: 25824104

11. Oliva R, Ji C, Atienza-Grande G, Huguet-Tapia JC, Perez-Quintero A, Li T, Eom

JS, Li C, Nguyen H, Liu B, Auguy F, Sciallano C, Luu VT, Dossa GS, Cunnac S, Schmidt SM, Slamet-Loedin IH, Vera Cruz C, Szurek B, Frommer WB, White FF, Yang B. Broad-spectrum resistance to bacterial blight in rice using genome editing. Nat Biotechnol. 2019; 37(11):1344-1350. doi:10.1038/s41587-019-0267-z PMID: 31659337

12. Xu Z, Xu X, Gong Q, Li Z, Li Y, Wang S, Yang Y, Ma W, Liu L, Zhu B, Zou L, Chen G. Engineering broad-spectrum bacterial blight resistance by simultaneously disrupting variable TALE-binding elements of multiple susceptibility genes in rice. Mol Plant. 2019; 12(11):1434-1446. doi:10.1016/j.molp.2019.08.006 PMID: 31493565

13. Li T, Liu B, Spalding MH, Weeks DP, Yang B. High-efficiency TALEN-based gene editing produces disease-resistant rice. Nat Biotechnol. 2012; 30(5):390-392. doi:10.1038/nbt.2199 PMID: 22565958

14. Hutin M, Pérez-Quintero AL, Lopez C, Szurek B. MorTAL Kombat: The story of defense against TAL effectors through loss-of-susceptibility. Front Plant Sci. 2015; 6:535. doi:10.3389/fpls.2015.00535 PMID: 26236326

15. Blanvillain-Baufumé S, Reschke M, Solé M, Auguy F, Doucoure H, Szurek B, Meynard D, Portefaix M, Cunnac S, Guiderdoni E, Boch J, Koebnik R. Targeted promoter editing for rice resistance to *Xanthomonas oryzae* pv. *oryzae* reveals differential activities for *SWEET14*-inducing TAL effectors. Plant Biotechnol J. 2017; 15(3):306-317. doi:10.1111/pbi.12613 PMID: 27539813

16. Zaka A, Grande G, Coronejo T, Quibod IL, Chen CW, Chang SJ, Szurek B, Arif M, Cruz CV, Oliva R. Natural variations in the promoter of *OsSWEET13* and *OsSWEET14* expand the range of resistance against *Xanthomonas oryzae* pv. *oryzae*. PLoS One. 2018; 13(9):e0203711. doi:10.1371/journal.pone.0203711 PMID: 30212546

17. Huang S, Antony G, Li T, Liu B, Obasa K, Yang B, White FF. The broadly effective recessive resistance gene *xa5* of rice is a virulence effector-dependent quantitative trait for bacterial blight. Plant J. 2016; 86(2):186-194. doi:10.1111/tpj.13164 PMID: 26991395

18. Li J, Li Y, Ma L. CRISPR/Cas9-based genome editing and its applications for functional genomic analyses in plants. Small Methods. 2019; 3(3):1800473. doi:10.1002/smtd.201800473 PMID: 27895652

19. Van Hop D, Phuong Hoa PT, Quang ND, Ton PH, Ha TH, Van Hung N, et al. Biological control of *Xanthomonas oryzae* pv. *oryzae* causing rice bacterial blight disease by Streptomyces toxytricini VN08-A-12, isolated from soil and leaf-litter samples in Vietnam. Biocontrol Sci. 2014; 19(3):103-111. doi:10.4265/bio.19.103 PMID: 25252641

20. Furuya N, Taura S, Goto T, Trong Thuy B, Huu Ton P, Tsuchiya K, Yoshimura A. Diversity in virulence of *Xanthomonas oryzae* pv. *oryzae* from Northern Vietnam. Japan Agric Res Q. 2012; 46(4):329-338. doi:10.6090/jarq.46.329

21. Nguyen HT, Vu QH, Van Mai T, Nguyen TT, Vu LD, Nguyen TT, Nguyen LV, Vu HTT, Nong HT, Dinh TN, Toshitsugu N, Vu LV. Marker-Assisted Selection of *Xa21* conferring resistance to bacterial leaf blight in indica rice cultivar LT2. Rice Sci. 2018; 25(1):52-56. doi:10.1016/j.rsci.2017.08.004

22. Jiang N, Yan J, Liang Y, Shi Y, He Z, Wu Y, Zeng Q, Liu X, Peng J. Resistance genes and their interactions with bacterial blight/leaf streak pathogens (*Xanthomonas oryzae*) in rice (*Oryza sativa* L.) - an updated review. Rice. 2020; 13(1):3. doi:10.1186/s12284-019-0358-y PMID: 31915945

23. Eom JS, Luo D, Atienza-Grande G, Yang J, Ji C, Thi Luu V, Huguet-Tapia JC, Char SN, Liu B, Nguyen H, Schmidt SM, Szurek B, Vera Cruz C, White FF, Oliva R, Yang B, Frommer WB. Diagnostic kit for rice blight resistance. Nat Biotechnol. 2019; 37(11):1372-1379. doi:10.1038/s41587-019-0268-y PMID: 31659338

24. Manh Bao T, Thi Hop T, Thi Tiec T, Thi Nhung N, Van Hoan N. Results of breeding TBR225 rice cultivar. Vietnam J. Agri. Sci. 2016; 14(9):1360-1367. In Vietnamese. Available from: http://www1.vnua.edu.vn/tapchi/Upload/15112016-so%208.7.pdf

25. Zhou J. Host target genes of the *Xanthomonas oryzae* pv . *oryzae* type III effectors for bacterial blight in rice. Graduate Theses and Dissertations, The Iowa State University. 2015. Available from: https://lib.dr.iastate.edu/cgi/viewcontent.cgi?article=5476&context=etd

26. Li T, Huang S, Zhou J, Yang B. Designer TAL effectors induce disease susceptibility and resistance to *Xanthomonas oryzae* pv. *oryzae* in rice. Mol Plant. 2013; 6(3):781-789. doi:10.1093/mp/sst034 PMID: 23430045

27. Herbert L, Meunier AC, Bes M, Vernet A, Portefaix M, Durandet F, Michel R, Chaine C, This P, Guiderdoni E, Périn C. Beyond seek and destroy: how to generate allelic series using genome editing tools. Rice. 2020; 13(1):5. doi:10.1186/s12284-020-0366-y PMID: 31993780

28. Zhou H, Liu B, Weeks DP, Spalding MH, Yang B. Large chromosomal deletions and heritable small genetic changes induced by CRISPR/Cas9 in rice. Nucleic Acids Res. 2014; 42(17):10903-10914. doi:10.1093/nar/gku806 PMID: 25200087

29. Hiei Y, Ohta S, Komari T, Kumashiro T. Efficient transformation of rice (*Oryza sativa* L.) mediated by *Agrobacterium* and sequence analysis of the boundaries of the T- DNA. Plant J. 1994; 6(2):271-282. doi:10.1046/j.1365-313X.1994.6020271.x PMID: 7920717

30. Bai J, Choi SH, Ponciano G, Leung H, Leach JE. *Xanthomonas oryzae* pv. *oryzae* avirulence genes contribute differently and specifically to pathogen aggressiveness. Mol Plant Microbe Interact. 2000; 13(12):1322-1329. doi:10.1094/MPMI.2000.13.12.1322 PMID: 11106024

31. Ma X, Chen L, Zhu Q, Chen Y, Liu YG. Rapid decoding of sequence-specific nuclease-induced heterozygous and biallelic mutations by direct sequencing of

PCR products. Mol Plant. 2015; 8(8):1285-1287. doi:10.1016/j.molp.2015.02.012 PMID: 25747846

32. Razzaq A, Saleem F, Kanwal M, Mustafa G, Yousaf S, Imran Arshad HM, Hameed MK, Khan MS, Joyia FA. Modern trends in plant genome editing: An inclusive review of the CRISPR/Cas9 Toolbox. Int J Mol Sci. 2019; 20(16):4045. doi:10.3390/ijms20164045 PMID: 31430902

33. Wang F, Wang C, Liu P, Lei C, Hao W, Gao Y, Liu YG, Zhao K. Enhanced rice blast resistance by CRISPR/Cas9-Targeted mutagenesis of the ERF transcription factor gene *OsERF922*. PLoS One. 2016;11(4):e0154027. doi:10.1371/journal.pone.0154027 PMID: 27116122

34. Zhou H, He M, Li J, Chen L, Huang Z, Zheng S, Zhu L, Ni E, Jiang D, Zhao B, Zhuang C. Development of commercial thermo-sensitive genic male sterile rice accelerates hybrid rice breeding using the CRISPR/Cas9-mediated TMS5 editing system. Sci Rep. 2016; 6:37395. doi:10.1038/srep37395 PMID: 27874087

35. Zhang A, Liu Y, Wang F, Li T, Chen Z, Kong D, Bi J, Zhang F, Luo X, Wang J, Tang J, Yu X, Liu G, Luo L. Enhanced rice salinity tolerance via CRISPR/Cas9-targeted mutagenesis of the *OsRR22* gene. Mol Breed. 2019; 39:47. doi:10.1007/s11032-019-0954-y PMID: 32803201

36. Zafar K, Khan MZ, Amin I, Mukhtar Z, Yasmin S, Arif M, Ejaz K, Mansoor S. Precise CRISPR-Cas9 mediated genome editing in super Basmati rice for

resistance against bacterial blight by targeting the major susceptibility gene. Front Plant Sci. 2020; 11:575. doi:10.3389/fpls.2020.00575 PMID: 32595655

37. Kim YA, Moon H, Park CJ. CRISPR/Cas9-targeted mutagenesis of *Os8N3* in rice to confer resistance to *Xanthomonas oryzae* pv. *oryzae*. Rice. 2019; 12(1):67. doi:10.1186/s12284-019-0325-7 PMID: 31446506

38. Molla KA, Shih J, Yang Y. Single-nucleotide editing for zebra3 and wsl5 phenotypes in rice using CRISPR/Cas9-mediated adenine base editors. *aBIOTECH*. 2020;1(2):106-118. doi:10.1007/s42994-020-00018-x

39. Chen LQ. SWEET sugar transporters for phloem transport and pathogen nutrition. New Phytol. 2014; 201(4):1150-1155. doi:10.1111/nph.12445 PMID: 24649486

40. Yang J, Luo D, Yang B, Frommer WB, Eom JS. SWEET11 and 15 as key players in seed filling in rice. New Phytol. 2018; 218(2):604-615. doi:10.1111/nph.15004 PMID: 293935104

41. Doucouré H, Pérez-Quintero AL, Reshetnyak G, Tekete C, Auguy F, Thomas E, Koebnik R, Szurek B, Koita O, Verdier V, Cunnac S. Functional and genome sequence-driven characterization of *tal* effector gene repertoires reveals novel variants with altered specificities in closely related malian *Xanthomonas oryzae* pv. *oryzae* strains. Front Microbiol. 2018; 9:1657. doi:10.3389/fmicb.2018.01657 PMID: 30127769

42. Chu Z, Yuan M, Yao J, Ge X, Yuan B, Xu C, Li X, Fu B, Li Z, Bennetzen JL,

Zhang Q, Wang S. Promoter mutations of an essential gene for pollen development result in disease resistance in rice. Genes Dev. 2006; 20(10):1250-1255. doi:10.1101/gad.1416306 PMID: 16648463

43. Pérez-Quintero AL, Rodriguez-R LM, Dereeper A, López C, Koebnik R, Szurek B, Cunnac S. An Improved Method for TAL Effectors DNA-Binding Sites Prediction Reveals Functional Convergence in TAL Repertoires of Xanthomonas oryzae Strains. PLoS One. 2013; 8(7):68464. doi:10.1371/journal.pone.0068464 PMID: 23869221

Supporting information

S1_raw_images. Original photograph used in Fig 2 for the RT-PCR gels panel.

S2_raw_images. Original photograph used in Fig 3C for the RT-PCR gels panel.

S1 Fig. Nucleotide sequence of the *OsSEET14* promoter in TBR225.

S2 Fig. Virulence of Vietnamese *Xoo* strains VXO_11 and VXO_15 on TBR225 rice. Grey points correspond to individual lesion length measurements while the black points indicate the calculated average value. The line range represents standard deviation.

S3 Fig. Picture of an individual plant from the homozygous mutant rice lines L-5.7(-6).

S4 Fig. Talvez scoring of AvrXa7, PthXo3 and TalF target EBES in the edited *OsSWEET14* promoter allele sequences. Score values are represented both by the length of the horizontal bar and a fill color scale. Higher Talvez prediction scores reflect a better match between a predicted EBE and the sequence of RVD of the query TALE.

S5 Fig. Amplicon sequencing of predicted off-target sites for the *OsSWEET14* promoter-sgRNA in annotated exons of the TBR225 edited line L-5.7(-6). Potential unintended target sequences including the PAM are highlighted in boxes. They are all identical to the expected wild type Nipponbare sequences.

S1 Table. Key figures on the TBR225 transformation procedure for *OsSWEET14* promoter editing.

S2 Table. Output of the CCTop tool used with the *OsSWEET14* promoter sgRNA for off-target prediction on the rice Nipponbare genome.

Click here to access/download;Figure;Fig1.tif

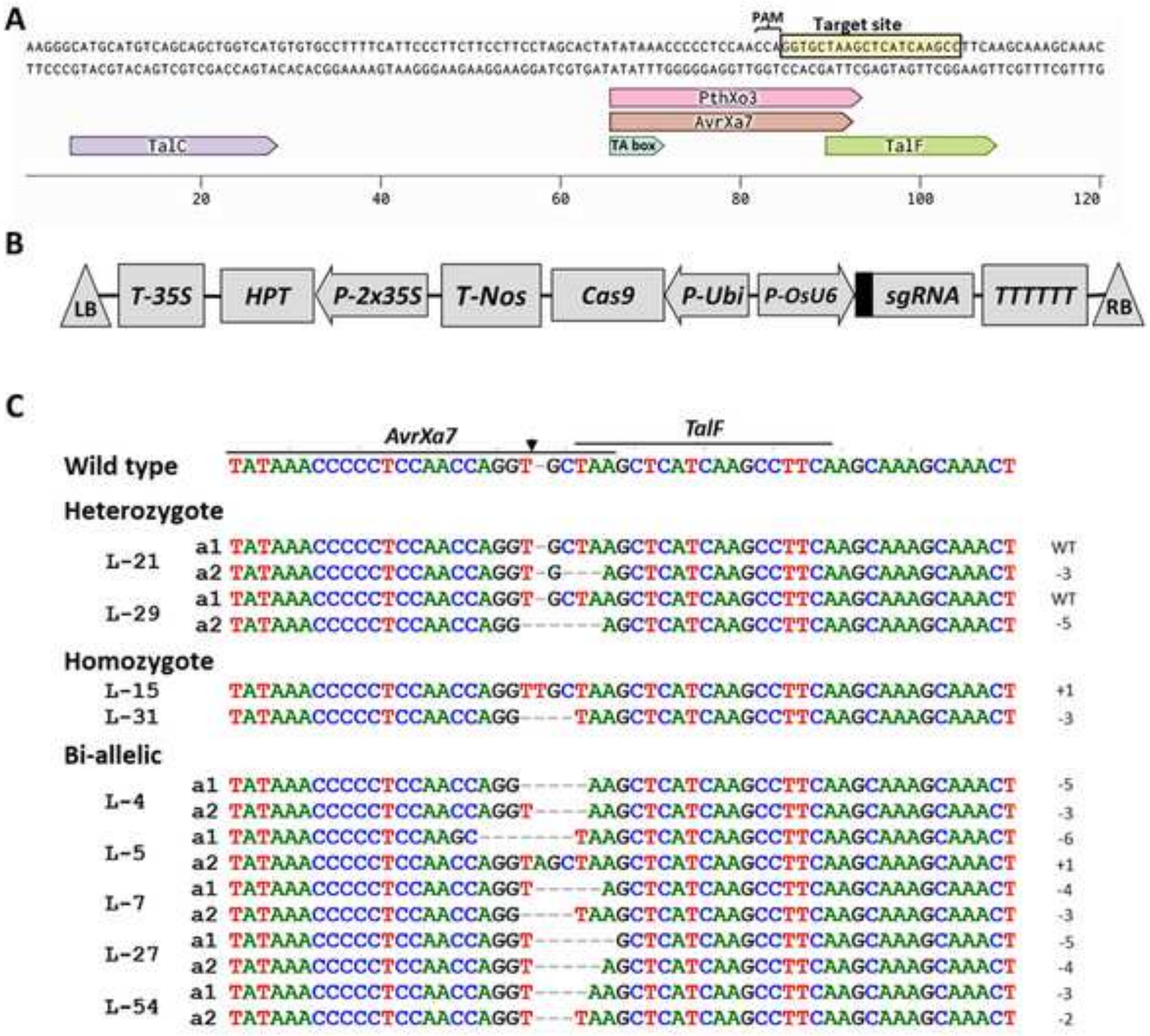

Click here to access/download;Figure;Fig2.tif

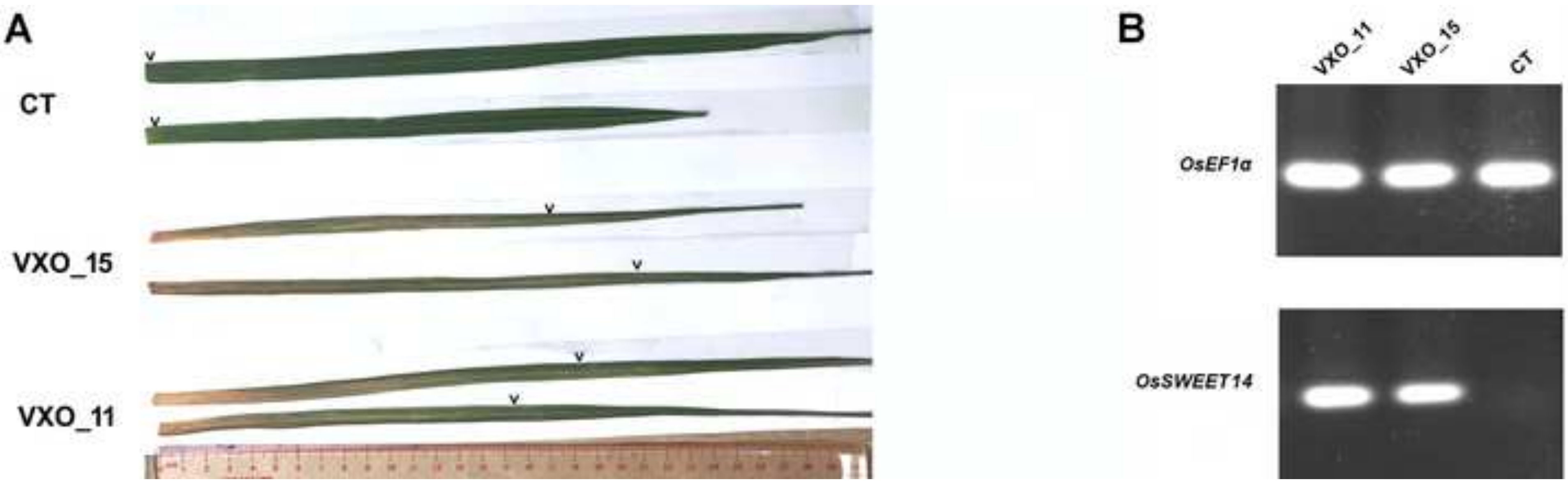

Fig3

Click here to access/download;Figure;Fig3.tif

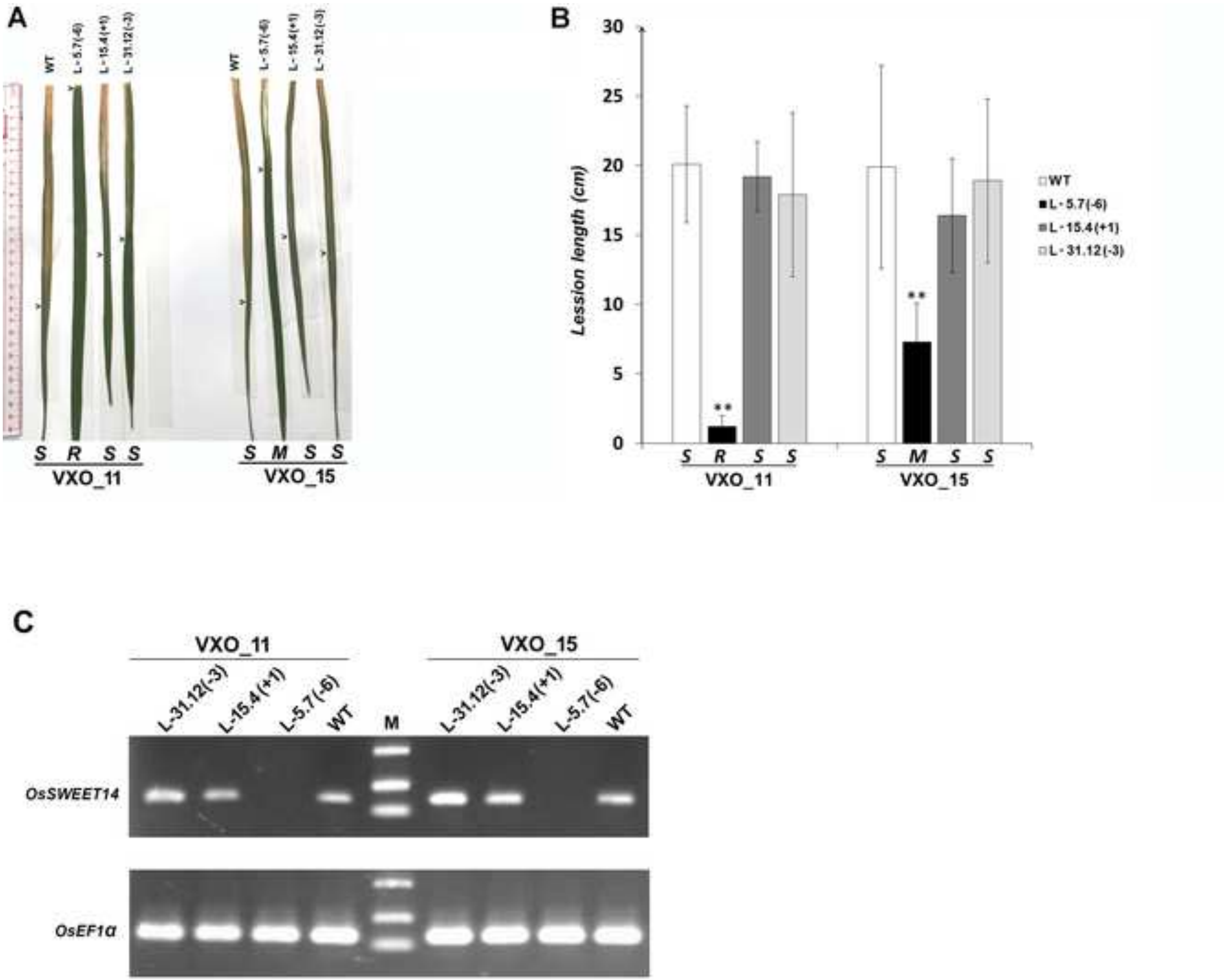

Click here to access/download
Supporting Information - Compressed/ZIP File Archive
Supporting Information.rar

Full title:

Improved bacterial leaf blight disease resistance in the major elite Vietnamese rice cultivar TBR225 via editing of the *OsSWEET14* promoter

Short title:

Improved bacterial leaf blight disease resistance in Vietnamese rice cultivar TBR225

Nguyen Duy Phuong[1¶], Tran Lan Dai[1,2¶], Pham Thu Hang[1], Phung Thi Thu Huong[1], Nguyen Thanh Ha[1], Pham Phuong Ngoc[1,#a], Florence Auguy[3], Bui Thi Thu Huong[4], Tran Manh Bao[5], Sebastien Cunnac[3], Pham Xuan Hoi[1]*

[1]*Department of Molecular Pathology, Institute of Agricultural Genetics, Vietnam Academy of Agricultural Sciences, Hanoi, Vietnam.*

[2]*Department of Applied Biology and Agriculture, Faculty of Natural Sciences, Quynhon University, Quynhon, Vietnam.*

[3]*PHIM Plant Health Institute, Univ Montpellier, IRD, CIRAD, INRAE, Institut Agro, Montpellier, France~~IRD, Cirad, Univ Montpellier, IPME, Montpellier, France~~.*

[4]*Vietnam National University of Agriculture, Hanoi, Vietnam.*

[5]*ThaiBinh Seed Corporation, Thaibinh, Vietnam.*

* Corresponding author: xuanhoi.pham@gmail.com

[¶] These authors contributed equally to this work.

[#a] Current address: Faculty of Biology, Hanoi University of Sciences, Hanoi, Vietnam.

Abstract

TBR225 is one of the most popular commercial rice ~~variety~~varieties in Northern Vietnam. However, this variety is very susceptible to bacterial leaf blight (BLB), a disease caused by *Xanthomonas oryzae* pv. *oryzae* (*Xoo*) which inflicts important yield losses. *OsSWEET14* belongs to the *SWEET* ~~(*Sugars Will Eventually be Exported Transporter*)~~ gene family that encodes sugar transporters. Together with other Clade III members, it behaves as a susceptibility (*S*) gene whose induction by Asian *Xoo* Transcription-Activator-Like Effectors (TALEs) is absolutely necessary for disease. In this study, we sought to introduce BLB resistance in the TBR225 elite variety. First, two Vietnamese *Xoo* strains were shown to up-regulate *OsSWEET14* upon TBR225 infection. To investigate if this induction is connected with disease susceptibility, nine TBR225 mutant lines with mutations in the AvrXa7, PthXo3 or TalF TALEs DNA target sequences of the *OsSWEET14* promoter were obtained using the CRISPR/Cas9 editing system. Genotyping analysis of T_0 and T_1 individuals showed that mutations were stably inherited. None of the examined agronomic traits of three transgene-free T2 edited lines were significantly different from those of wild-type TBR225~~All examined agronomic traits of three transgene-free T_2 lines were not significantly different from those of wild-type TBR225~~. Importantly, one of these T_2 lines, harboring the largest homozygous 6-bp deletion, displayed decreased *OsSWEET14* expression as well as a significantly reduced susceptibility to ~~both~~ a Vietnamese *Xoo* strains and complete resistance to ~~one~~ the other

Formatted: Font: Italic

one~~of them~~. Our finding indicated that CRISPR/Cas9 is a useful and effective approach to improve BLB resistance of commercial elite rice varieties.

Keywords**: **Bacterial leaf blight; CRISPR/Cas9; Xanthomonas oryzae* **pv.** *oryzae; OsSWEET14; TBR225; transgene-free plants.

Introduction

Bacterial leaf blight (BLB) caused by *Xanthomonas oryzae* pv. *oryzae* (*Xoo*) is a major bacterial disease that causes 10%-20% annual reduction in rice production worldwide [1]. The use of improved rice varieties resistant to *Xoo* is probably the most efficient, economical and environmentally-friendly way to control BLB.

The virulence of *Xoo* depends on the transcriptional activation of specific host disease-susceptibility (*S*) genes by a subgroup of bacterial type III effectors, called transcription activator-like effectors (TALEs) [2]. Upon translocation into the plant cell, TALEs bind to specific host nuclear gene promoter sequences termed Effector-Binding Elements (EBEs) and induce target gene expression to the benefit of the pathogen. The central repetitive domain of TALEs is responsible for DNA target sequence binding. DNA binding involves recognition principles that have been largely deciphered and applied to the computational prediction of TALEs target DNA sequences [3,—4]. This and earlier work has fostered the identification of TALEs transcriptional targets in the rice genome and ultimately, of rice BLB *S* genes [2].

All *Xoo* strains recurrently target *S* genes belonging to the *SWEET* gene family and coding for transmembrane sugar exporter proteins [3]. The over accumulation of SWEETs due to TALE induction is presumed to provide an additional ration of apoplastic carbohydrates for full bacterial pathogen multiplication and disease expression [5]. Although all five rice clade III *SWEET* genes can function as *S* genes for bacterial

Formatted: Font: (Default) Times New Roman, 13 pt

Formatted: Font: Italic

blight, only three, namely *OsSWEET11*, *OsSWEET13* and *OsSWEET14*, are known to be targeted by several unrelated TALEs in nature [6–11]. *OsSWEET11* is activated by PthXo1 [6], *OsSWEET13* is targeted by different variants of PthXo2 [11,~~–~~12], while *OsSWEET14* is a target of multiple TAL effectors, including AvrXa7, PthXo3, TalC and TalF [7–9].

Previous studies established that rice ~~recessive~~ resistance to *Xoo* resulting from "TALE-unresponsive" alleles can be conferred by natural DNA polymorphisms or targeted editions in EBEs located in *OsSWEET* genes promoters of rice germplasm accessions or engineered rice varieties, respectively [6,13–16]. For example, early resistance engineering work has used TALENs to individually alter the AvrXa7, TalC or TalF EBEs in the *OsSWEET14* promoter and successfully obtained resistance to some Asian *Xoo* strains [13,15]. However, ~~many~~ strains collected in Asian countries such as China, Japan, Phillippines, Taiwan, Thailand, India, Nepal or South Korea can express combinations of up to three major TALEs redundantly targeting clade III *OsSWEET* genes with either PthXo3 or AvrXa7 being ~~often~~ occasionally associated ~~to~~ with PthXo2 [11,17]. Broad BLB resistance engineering thus required multiplex *OsSWEET* promoters EBE editing using the CRISPR/Cas9 system [11,12].

The clustered regularly interspaced short palindromic repeats/CRISPR-associated protein-9 nuclease (CRISPR/Cas9) system is a simple and efficient gene-editing tool developed in the past few years. Moreover, the targeted mutations generated by CRISPR/Cas9 can be ~~faithfully and~~ stably transmitted to the next generation. Thus,

CRISPR/Cas9 has become a routine tool in plant laboratories around the world to create various mutants for many applications, including the genetic improvement of crops [18].

BLB is a major rice disease which occurs in many rice cultivating areas of Vietnam [19,20]. Most Vietnamese commercial rice varieties, including TBR225, are susceptible to BLB, resulting in annual yield loss of about 15 – 30% on average [21]. A few studies have identified rice resistance genes effective against Vietnamese *Xoo* lineages [20,21]. However, no information is currently available on the nature of Vietnamese *Xoo* TALEs and their corresponding *S* genes.

Despite the large number of mapped rice BLB resistance genes [22], there is a need for alternative breeding approaches that enable the rapid introduction of broad BLB resistance in elite varieties in order to cope with swift pathogen populations adaptive shifts in the fields [11,23].

Here, we report on the identification of *OsSWEET14* as a transcriptional target of Vietnamese *Xoo*. CRISPR/Cas9-mediated mutagenesis of the *OsSWEET14* promoter in TBR225, a major elite variety in rice production areas of North Vietnam is shown to confer BLB resistance without detectable yield penalty. ~~For the first time, t~~This study found this quintessential *S* gene to be associated with the virulence of Vietnamese *Xoo* strains. This is an important step for the future design and implementation of broad-spectrum BLB-resistant in elite rice varieties using genome editing in Vietnam.

Materials and methods

Plant and pathogen materials

Rice cultivar TBR225 (*Oryza sativa* L. ssp. *indica*) were obtained from ThaiBinh Seed Cor. [24]. All edited and wild-type (WT) TBR225 plants were grown in a net-house under the following average conditions: 30°C for 14 h (light) and 25°C for 10 h (dark) with 80% humidity. The *Xoo* VXO_11 and VXO_15 strains used in this study were isolated from diseased leaves collected in Hanoi-Vietnam in 2013 and 2016, respectively. Bacteria were cultured as described in Zhou et al. (2015) [25].

Gene expression analysis

Gene expression analyses were carried out as described previously [26] by RT-PCR method. The rice leaves were infiltrated with the indicated bacterial strains and used for total RNA extraction 48 h post inoculation using the TRIzol reagent (Invitrogen, USA). One microgram of RNA was used for each RT-PCR with oligo (dT) primer followed by PCR with *OsSWEET14*-specific primers (forward 5′-ACTTGCAAGCAAGAACAGTAGT-3′ and reverse 5′-ATGTTGCCTAGGAGACCAAAGG-3′). An *Eppendorf Mastercycler ep Gradient S* was used for 35 PCR cycles. The *OsEF1α* gene was used as a constitutive control [15] using specific primers (forward 5′-GAAGTCTCATCCTACCTGAAGAAG-3′ and reverse 5′-GTCAAGAGCCTCAAGCAAGG-3′).

gRNA design

The *OsSWEET14* promoter (GenBank, accession number: AP014967.1) was amplified by PCR with forward primer 5’-TTGCGGCTCATCAGTTTCTC-3’ and reverse primer 5’-CTAGGAGACCAAAGGCGAAG-3’ from genomic DNA of TBR225 rice plants and ligated in pGEM-T Easy vector (Promega) for sequencing. The gRNA target sequence (Fig 1A) for editing the TBR225 *OsSWEET14* promoter was designed based on the sequence of the cloned TBR225 *OsSWEET14* promoter using a combination of two bioinformatics tools CRISPR-P v2.0 (http://crispr.hzau.edu.cn/CRISPR2/) and CCTop (https://crispr.cos.uni-heidelberg.de/). ~~The~~ A gRNA sequence with high on-target and low off-target scores in both prediction tools was chosen for vector construction.

Fig 1. CRISPR/Cas9-induced *OsSWEET14* promoter modification in TBR225 rice. (A) A region of the *OsSWEET14* promoter containing four EBEs (TalC, PthXo3, AvrXa7 and TalF) and putative TATA box from TBR225. The target site (complementary to the guide RNA) is shown in the box, immediately following the protospacer adjacent motif (PAM). (B) T-DNA region of the CRISPR/Cas9-mediated genome editing construct carrying OsSWEET14-sgRNA (indicated by the black box). The expression of *Cas9* is driven by the maize *ubiquitin* promoter (P-Ubi); the expression of the OsSWEET14-sgRNA is driven by the rice *OsU6* promoter (P-OsU6a); the expression of *HPT* is driven by two *CaMV35S* promoters (P-2×35S); T-35S, T-Nos and TTTTTT: gene terminators; LB and RB: left and right border, respectively. (C) Alignment of the *OsSWEET14*

promoter fragment in the nine T_0 transgenic TBR225 rice plants edited in the AvrXa7, PthXo3 and TalF EBEs. The lines on top of the wild-type sequence represent the binding sites of AvrXa7, PthXo3 and TalF. The arrow indicates the expected cutting site of the Cas9 complex used in this study. The labels on the left indicate the name of examined mutant lines; (a1) and (a2) distinguish alleles in the same line. The numbers on the right indicate the type of mutation and the number of nucleotides involved; (+) and (-) indicate insertion and deletion, respectively.

Vector construction

The Cas9 rice expression vector (pUbi-Cas9) [27] and the sgRNA expression vector (pENTR-sgRNA) under the control of the *OsU6* promoter [28] were used to construct the pCas9/OsSWEET14-gRNA expression vector. The complementary oligonucleotides with appropriate 4-bp overhangs were synthesized by Macrogen (Korea). After heat denaturation, the complementary oligonucleotides (5′-gtgtGGTGCTAAGCTCATCAAGCC-3’ and 5′-aaacGGCTTGATGAGCTTAGCACC-3’) were first annealed to each other, phosphorylated, and ligated into the *Bsa*I-digested vector pENTR-sgRNA. The integrity of the inserted fragment was verified by sequencing. Subsequently, the sgRNA cassette was cloned into pUbi-Cas9 using the Gateway LR clonase (Life Technologies) (Fig 1B). The resulting construct was confirmed by Sanger sequencing of the insertion junctions.

Agrobacterium-mediated rice transformation

The pCas9-OsSWEET14-gRNA was electroporated into *Agrobacterium tumefaciens* EHA105 and the resulting strain was used to transform rice using the method described by Hiei et al. (1994) [29]. The presence of the transgene in the genome of T_0 hygromycin-resistant plants or segregating T_1 individuals was evaluated by PCR using 5′-ATGGCCCCAAAGAAGAAG-3′ and 5′- GCCTCGGCTGTCTCGCCA-3′ primers specific for *Cas9*. T1 individuals were analyzed by PCR using *Cas9*, *OsSWEET14-gRNA* (5′- GGATCATGAACCAACG-3′ and 5′- GAATTCGATATCAAGCTT-3′) and *HPT* (5’-AAACTGTGATGGACGACACCGT-3’ and 5′- GTGGCGATCCTGCAAGCTCC - 3′) specific diagnostic primer pairs together with a positive control pair (5′-TTGCGGCTCATCAGTTTCTC-3′ and 5′- TGGATCAGATCAAAGGCAAC -3′) specific to the *OsSWEET14* promoter.

Bacterial blight inoculation

Rice cultivation and disease assays were done according to the methods of Blanvillain-Baufumé et al. (2017) [15]. Bacteria were cultured in PSA media (10 g/liter peptone, 10 g/liter sucrose, 1 g/liter glutamic acid, 15 g/liter Bacto Agar) at 28°C for two days [30] and inoculated at an optical density (OD_{600}) of 0.5 (infiltrations) or 0.4 (leaf clipping) in water. For lesion length measurements, at least three inoculated leaves per plant and three

plants for each line were measured 14 days after inoculation (DAI), and scored as follows: high resistance (lesion length < 8 cm), moderate resistance (lesion length 8-12 cm) and susceptibility (lesion length > 12 cm). For gene expression analyses, 4-cm leaf sections which were infiltrated with bacterial ~~solution~~ suspensions were collected at 48 h after inoculation for RNA extraction. Experiments included samples from three pooled biological replicate leaves. The plants inoculated with distilled water only were used as negative controls ~~in all experiments~~.

Analysis of *OsSWEET14* edited allele sequences

To determine the nature of the mutation at the target site, all transgenic T_0 or T_1 plants were analyzed by PCR using genomic DNA (50 ng) as a template and *OsSWEET14* specific primers (5′-TTGCGGCTCATCAGTTTCTC-3′ and 5′-TGGATCAGATCAAAGGCAAC -3′). The PCR products were directly sequenced using the Sanger method. The sequencing chromatograms were decoded using the Degenerate Sequence Decoding method [31] in order to identify the mutations.

Evaluation of major agronomic traits under net-house conditions

WT and selected mutant plants were planted under net-house conditions in a randomized pot design experiment. At maturity, five plants of each line were investigated for the following agronomic traits: growth duration, plant height, number of tillers per plant,

number of grains per panicle, number of filled grains per panicle and yield (seed mass) per plant. The experiment was repeated three times, so a total of fifteen plants were evaluated for each line.

Analysis of potential off-target ~~sequences~~editing

O~~O~~ff-target sequences were predicted ~~by~~with the ~~–~~CCTop tool~~s~~ (https://crispr.cos.uni-heidelberg.de~~/~~) **against the** *OsSWEET14* promoter ~~-~~**sgRNA** and the rice Nipponbare genome with default parameters. A total of 18 potential off-target sequences were identified. Three of them ~~, of which 3~~ were located in coding regions (Table S2). These regions were amplified by PCR using the specific primers listed in Table S2 and analyzed by sequencing.

Formatted: Heading 2, Left, Line spacing: single

Formatted: fontstyle01, Font: (Default) Times New Roman, 13 pt

Formatted: fontstyle01, Font: (Default) Times New Roman, 13 pt

Formatted: fontstyle01, Font: (Default) Times New Roman, 13 pt

Formatted: fontstyle01, Font: (Default) Times New Roman, 13 pt, Not Bold, Font color: Auto

Formatted: fontstyle01, Font: (Default) Times New Roman, 13 pt, Not Bold, Font color: Auto

Formatted: fontstyle01, Font: (Default) Times New Roman, 13 pt

Results

Vietnamese *Xoo* strains induce *OsSWEET14* during infection of the TBR225 rice variety

OsSWEET14/Os11N3 was previously identified as a susceptibility gene for *Xoo* strains relying on either of the AvrXa7, PthXo3, TalF (formerly Tal5) or TalC TALEs for infection of the rice cultivars Nipponbare and Kitaake [11]. Because *Xoo* strains tend to frequently target this gene, we first sequenced ~~In this study,~~ a region of the *OsSWEET14*

Formatted: Font: Italic

promoter from rice cultivar TBR225 ~~was sequenced~~ to examine if it also carries documented target EBEs. Based on the Nipponbare genome sequence in database (AP014967.1), the region encompassing 1343 bp sequence upstream and 52 bp sequence downstream of the predicted transcription start site of *OsSWEET14* gene from TRB225 rice cultivar was PCR amplified and sequenced (S1 Fig). The promoter region including the putative TATA box (TATAAA) and the AvrXa7, PthXo3, TalF/Tal5 and TalC EBEs (Fig 1A), located 319 bp to 216 bp upstream of the ATG initiation codon, showed 100% identity to the Nipponbare sequence. This therefore implied that in principle, the TBR225 *OsSWEET14* promoter can be recognized by characterized major *Xoo* TALEs.

As illustrated by the representative experiment of Fig 2A, we also challenged TBR225 plants with two Vietnamese *Xoo* strains VXO_11 and VXO_15, both originating from the Hanoi province, using leaf clipping assays. We consistently obtained typical extended disease lesions 14 days after inoculation (25.5 cm and 26.6 cm average lesions length for VXO_11 and VXO_15, respectively in the experiment of S2 Fig), indicating that the TBR225 variety is susceptible to BLB.

To test if *OsSWEET14* is a potential direct virulence target of Vietnamese *Xoo* strains, we infiltrated TBR225 rice leaves with the two Vietnamese *Xoo* strains. Forty-eight hours post infiltration, TBR225 plants inoculated with VXO strains displayed a strong induction of *OsSWEET14* relative to water controls (Fig 2B). These results suggest that *OsSWEET14* is a transcriptional target of VXO strains and that it may act as a susceptibility gene in TBR225.

Fig 2. *OsSWEET14* is likely a susceptibility gene for Vietnamese *Xoo* strains in rice cultivar TBR225. (A) Representative images of the disease lesions obtained 14 days after leaf clipping inoculation of TBR225 rice leaves with Vietnamese *Xoo* strains VXO_11 and VXO_15 or with water (CT). The chevrons above the leaves indicate the maximum visible extent of lesions away from the inoculation point on the left (B). *OsSWEET14* expression pattern obtained by RT-PCR two day post-infiltration of TBR225 rice leaves with Vietnamese *Xoo* strains. CT Plants were inoculated with water only. The experiment was repeated three times.

CRISPR/Cas9 design for *OsSWEET14* promoter editing

Our main objective was to engineer resistance to BLB caused by Vietnamese *Xoo* strains. To this end, we subsequently sought to specifically modify the *OsSWEET14* promoter in TBR225 rice with CRISPR/Cas9-mediated editing. Previous work revealed that while African *Xoo* strains rely on TalC and occasionally, TalF, all Asian *Xoo* strains use either PthXo3- or AvrXa7-like TALEs to activate *OsSWEET14* [11]. Because the *talC* gene is currently exclusively found in African strains, we reasoned that it is unlikely that Vietnamese strains carry a *talC* copy. Thus, to maximize our chances to perturb all remaining documented EBEs, we selected a 20-bp nucleotide target site overlapping the PthXo3, AvrXa7 and TalF EBEs and having a predicted cut site located near the 3'-end of

the AvrXa7 EBE (Fig 1A). The recombinant binary plasmid pCas9/OsSWEET14-gRNA for CRISPR/Cas9 mediated editing of *OsSWEET14* was transformed into the rice variety TBR225 via *Agrobacterium*-mediated transformation (S1 Table). A total of nine TBR225 transformants were selected from 10 independent PCR-validated transgenic T_0 TBR225 plants to further investigate CRISPR/Cas9-targeted mutagenesis of the *OsSWEET14* promoter. In order to decipher the nature of the editing events in *OsSWEET14*, the promoter sequencing data of transgenic lines were analyzed using the Degenerate Sequence Decoding software [31]. All 9 T_0 transgenic plants harbored at least an edition event (Fig 1C): two were heterozygous mutant/wild type, two had homozygous mutations, and five had bi-allelic mutations. Regarding the type of mutations, 66.7% were nucleotide deletions, 11.1% of the mutations were nucleotide insertions and no substitution was detected (Table 1).

Table 1. Frequencies of mutant genotypes and target mutation types in T_0 transgenic plants.

**Mutant genotype ratios[a] (%)**			**Mutation type ratios[b] (%)**		
**Heterozygote**	**Homozygote**	**Bi-allelic**	**Deletion**	**Insertion**	**Substitution**
22.2 (2/9)	22.2 (2/9)	55.6 (5/9)	66.7 (12/18)	11.1 (2/18)	0 (0/18)

[a] (Number of on-target mutant genotype/total number of on-target mutant genotypes) x 100%.
[b] (Number of allele mutation type/number of all allele mutation types) x 100%.

Inheritance of CRISPR/Cas9-induced mutations in the T_1 generation

To assess the inheritance of the CRISPR/Cas9-induced *OsSWEET14* mutations in the next generation, all T_0 mutant transgenic plants (Fig 1C) were allowed to self-pollinate, and T_1 transgenic plants were randomly selected in the progeny of T_0 plants for sequencing and analysis of their edited site (Table 2). All T_1 individuals derived from T_0 plants previously genotyped as homozygous possessed the same allele as their parent, suggesting stable inheritance of the mutations to the next generation. Similarly, the T1 progeny of each of both bi-allelic and heterozygous mutation T_0 lines showed a segregation ratio which is consistent with Mendelian segregation ($\chi^2 < \chi^2_{0.05,\ 2} = 5.99$), indicating that the CRISPR/Cas9-induced mutations in T_0 plants were transmitted as expected to the next generation. Interestingly, no new mutant allele was detected in the T_1 generation of both heterozygous mutants L-21 and L-27, even though most of them still carried the transgene. Overall, consistent with previous similar studies, our results indicate that the CRISPR/Cas9-mediated mutations generated here are stably transmitted to the next generation in a Medelian fashion.

Table 2. Transmission of CRISPR/Cas9 editing events to the T_1 generation.

**T_0 plant**	**Genotype**	**Allele(s)**	**No. of T_1 plants tested**	**Mutation inheritance in the T_1 generation**		**No. of T-DNA-free plants**
				**Alleles segregation**	**χ^2 (1:2:1)**	

L-4	Bi-allelic	-5/-3	32	10 (-5), 18 (-5/-3), 4 (-3)	2,750	5 (2*)
L-5	Bi-allelic	-6/+1	44	9 (-6), 22 (-6/+1), 13 (+1)	0,727	10 (2*)
L-7	Bi-allelic	-4/-3	38	14 (-4), 17 (-4/-3), 7 (-3)	3,000	11 (4*)
L-15	Homozygote	+1	5	5 (+1)	-	1 (1*)
L-21	Heterozygote	-3	26	3 (-3), 13 (-3/wt), 10 (wt)	3,769	7 (1*)
L-27	Bi-allelic	-5/-4	7	1 (-5), 3(-5/-4), 3 (-4)	1,286	0
L-29	Heterozygote	-5	33	6 (-5), 19 (-5/wt), 8 (wt)	1,000	2 (0*)
L-31	Homozygote	-3	15	15 (-3)	-	5 (5*)
L-54	Bi-allelic	-3/-2	21	3 (-3), 12 (-3/-2), 6(-2)	1,286	3 (0*)

"+" and "-" indicate respectively, insertion and deletion, of the indicated number of nucleotides.
"w", wild type.
*Number of homozygous mutant plants without T-DNA.

Selection of transgene-free mutant TBR225 rice lines

To identify T-DNA free T_1 rice plants containing a mutation in EBEs of the *OsSWEET14* promoter, PCR analysis was carried out using primers specific to *Cas9*, sgRNA and *HPT* sequences (Table 2). A T1 individual was considered devoid of the transgene if the control amplification of the *OsSWEET14* promoter was successful and if none of the PCR reactions with independent primer pairs designed on the T-DNA produced a detectable diagnostic band. The results of this PCR screen show that the T-DNA could be segregated out in the progeny of most T_0 lines, with 88.9% of the T_0 lines generating T-DNA-free progeny. In total, 44 of 221 analyzed edited T_1 plants did not generate a specific amplicon from the T-DNA construct and 15 of them were homozygous mutant

Formatted: Font: Italic

harboring the desired *OsSWEET14* modifications. Our results demonstrate that transgene-free, homozygous mutant individuals could be obtained in the segregating progeny of selfed T_0 individuals.

TBR225 *OsSWEET14* promoter editing confers resistance to Vietnamese *Xoo*

To characterize the BLB-resistance phenotype of the generated rice mutants, three T-DNA-free, homozygous TBR225 edited lines, namely, L-5.7(-6), L-31.12(-3) and L-15.4(+1) with *OsSWEET14* promoter alleles corresponding respectively to L-5-a1 (6bp deletion), L-31 (3bp deletion) and L-15 (1bp insertion) in Fig 1C, were established. Selected T_1 individuals were propagated to obtain T_2 seeds which were used to perform BLB susceptibility assays. Edited T_2 and WT TBR225 plants were inoculated by leaf-clipping with the VXO_11 and VXO_15 strains at the eight-week stage. The inoculated leaves of wild type TBR225 plants and of edited lines L-15.4(+1) and L-31.12(-3) developed long water-soaked lesions typical of BLB, ranging from 18.3 cm to 29.0 cm in length. In contrast, the edited line L-5.7(-6), harboring a longer 6-bp deletion at the target site, displayed high (1.2 cm average lesion length) and moderate (7.3 cm average lesion length) resistance to VXO_11 and VXO_15 strains, respectively (Fig 3). Means comparisons with a Tukey's HSD test further indicated that irrespective of the inoculated strain, the mean lesion lengths measured on the L-15.4(+1), L-31.12(-3) or wild type

lines were not significantly different. In contrast, the mean lesion lengths recorded on the L-5.7(-6) mutant line were significantly different from those obtained on the wild type and the two other edited lines challenged with either of the Vietnamese strains (Fig 3B). Furthermore, our off-target editing analysis on line L-5.7(-6) did not reveal unintended modifications of other annotated rice loci (Table S2 and Figure S5), indicating that the 6-bp deletion in the *OsSWEET14* promoter is probably responsible for this phenotype.

Formatted: Font: Italic

Consistent with disease assays and as shown in Figure 3C, whereas a semiquantitative RT-PCR signal for *OsSWEET14* expression was detected on the parental variety and the L-15.4(+1) and L-31.12(-3) edited lines following VXO_11 and VXO_15 infiltration, this amplicon was undetectable in the resistant L-5.7(-6) line.

Formatted: Font: Italic

In conclusion, this data shows that ~~whereas~~ the ~~a~~ 6-bp deletion in the AvrXa7/PthXo3 EBE reduces dramatically *OsSWEET14* expression following VXO strains inoculation and confers ~~measureable~~ resistance to ~~VXO~~these strains. In contrast, shorter modifications on the 3'-end of this EBE are insufficient to perturb *OsSWEET14* expression after inoculation and do not confer detectable protection ~~to~~ against the corresponding strains. ~~Furthermore~~Finally, while these results strongly support the view that *OsSWEET14* functions as a unique susceptibility gene in the interaction between strain VXO_11 and the TBR225 rice variety, the resistance to strain VXO_15 is not as dramatic and may suggest that other mechanisms partially counteract the effects of the AvrXa7/PthXo3 EBE 6-bp deletion in edited TBR225 plants.

Formatted: Font: Italic

Fig 3. BLB resistance assays for homozygous mutant rice lines L-5.7(-6), L-15.4(+1) and L-31.12(-3). (A) Leaves were photographed 14 days post-leaf clipping inoculation of *Xoo* strains VXO_11 and VXO_15; arrow heads indicate the end of the lesion. (B) Mean lesion lengths (bars) and standard deviations (error bars). Values were measured 14 days post-leaf clipping inoculation of two *Xoo* strains VXO_11 and VXO_15 and were computed from at least three leaves from each of three plants. Asterisks indicate significant differences relative to wild type plants (Tukey's HSD test; **P < 0.05). The number in the parentheses following the line name indicates the type of mutation and the number of nucleotides involved. The letters above strain labels indicate susceptibility score (R - high resistance; M – moderate resistance; S - susceptibility). The experiment was repeated three times. (C) *OsSWEET14* expression pattern obtained by RT-PCR two day post-infiltration of genome edited homozygous mutant rice lines L-31.12(-3), L-15.4(+1) and L-5.7(-6) and parental TBR225 rice leaves with Vietnamese *Xoo* strains. This experiment was repeated two times with similar results.

Formatted: Font: Not Bold

TBR225 *OsSWEET14* promoter edited lines agronomic performances are undistinguishable from the parental variety

To determine if and how mutations in the *OsSWEET14* promoter affect agronomic traits of TRB225 rice plants, three independent homozygous mutant lines were analyzed by measuring their growth duration, plant height, number of tillers per plant, number of grains per panicle, number of filled grains per panicle, yield per plant and amylose content under net-house conditions (see picture of S3 Fig). ANOVA tests and Student's *t* tests showed that the mutant lines displayed no significant difference to TBR225, in terms of the examined agronomic traits, under our net-house conditions (Table 3). These results suggest that the tested CRISPR/Cas9-induced mutations in the *OsSWEET14* promoter did not negatively impact the main agronomic traits of TBR225.

Table 3. Agronomic traits evaluation of homozygous T_2 mutant lines.

**Lines**	**Growth duration (day)**	**Plant height (cm)**	**No. of tillers per plant**	**No. of grains per panicle**	**No. of filled grains per panicle**	**Amylose content (%)**
WT	108.4 ± 1.1^a	86.6 ± 3.2^a	5 ± 0.7^a	144.4 ± 4.9^a	125 ± 4.5^a	13.2 ± 0.38^a
L-5.7(-6)	108 ± 1.2^a	86.4 ± 4.3^a	5.2 ± 0.4^a	144.2 ± 4.4^a	123.4 ± 5.5^a	13.7 ± 0.35^a
L-15.4(+1)	107.8 ± 0.8^a	86.4 ± 5.0^a	4.8 ± 0.4^a	147.8 ± 5.1^a	121.8 ± 3.0^a	13.5 ± 0.41^a
L-31.12(-3)	108 ± 1.2^a	88.4 ± 4.3^a	5.4 ± 0.5^a	144.6 ± 5.3^a	124.2 ± 7.4^a	13.8 ± 0.21^a

Five plants per line were measured. Experiments were repeated three time.
Means followed by the same letter do not differ significantly ($P < 0.05$).

Discussion

Recently, the CRISPR/Cas9 system has emerged as a powerful tool for gene editing in many organisms including plants. Because of its specificity and efficiency, this system

has been widely used to improve important agronomic traits of major crops such as rape, tomato, soybean, rice, wheat and maize [32]. Excluding easy-to-transform reference accessions such as Nipponbare and Kitaake that are widely used in the laboratory, the number of reports on the improvement of agriculturally relevant elite rice cultivars for pertinent traits using the CRISPR/Cas9 technology (see for example [33–36]) is gradually increasing but is still limited.

TBR225 [24], a major commercial rice variety cultivated in large areas of Northern Vietnam, has the advantages of early maturity, high and stable yield, as well as cooking quality. However, it is very susceptible to BLB. Here, the CRISPR/Cas9-mediated editing method was applied in order to rapidly improve the BLB resistance of TBR225 by modifying the AvrXa7, PthXo3 and TalF EBEs on the promoter of *OsSWEET14*. Of the three generated homozygous mutant lines tested for resistance, the one carrying the largest deletion at the target site (6 bp) showed a significantly improved resistance to infection with two *Xoo* strains VXO_11 and VXO_15. Therefore, using the major commercial rice variety TBR225 as an example, we illustrate the advantages of CRISPR/Cas9 tool for rice breeding.

In the present study, the frequency of individuals with CRISPR/Cas9-induced mutations in T_0 transgenic plants was 90%, which is similar to previous observation [28]. We obtained only two heterozygous mutant/wild type lines versus seven homozygous or bi-allelic mutant lines. This high frequency of mutated alleles is another proof that the CRISPR/Cas9 system is indeed an efficient tool for gene editing in plant. We also

observed the stable transmission of edited alleles to subsequent generations. This is a common phenomenon that has been repeatedly documented for rice plants carrying CRISPR/Cas9-induced mutations [33,35]. In this study, we obtained only two types of induced mutations ~~were observed~~ in T_0 plants: ~~single-nucleotide~~ insertion (11.1%) and deletion (66.7%), but no substitution ~~or combinations of the different mutation types~~were observed. In some earlier studies, new mutations were continuously obtained in the T_1 offspring of heterozygous T_0 mutants because ~~of the continuous activity of~~ the ~~CRISPR/~~Cas9 complex remains active on edited targets until the seed or PAM regions~~system~~ cease to be functional [35,37,38]. In contrast, here, all the T_1 plants generated from both heterozygous lines L-21 and L-29, regardless of whether they had a CRISPR/Cas9 T-DNA transgene integrated in their genome, did not show any new mutation possibly because CRISPR/Cas9 T-DNA transgene was no longer functional. We could also readily obtain transgene-free plants from most of the T_1 segregation populations without any laborious crossing or backcrossing steps, which illustrates an advantage of the CRISPR/Cas9 technology compared to conventional breeding.

Clade III SWEET family proteins are involved in a number of biological processes such as seed and pollen development or pathogen susceptibility [39]. Their inactivation has previously been shown to cause pleiotropic and/or detrimental effects. For example, both *ossweet11* single and *ossweet11-ossweet15* double Kitaake rice mutants showed defects in endosperm development and filling [40]. In addition, RNA-mediated silencing of either *Os11N3/OsSWEET14* [7] or *Os8N3/OsSWEET11* [6] in BLB resistant Kitaake

Formatted: Not Highlight

Formatted: Not Highlight

Field Code Changed

Formatted: Check spelling and grammar, Not Superscript/ Subscript

Field Code Changed

Field Code Changed

Formatted: Not Superscript/ Subscript

Field Code Changed

Formatted: Not Superscript/ Subscript

lines causes negative effects on seed production. In contrast, here, we show that T-DNA-free TBR225 plants harboring homozygous mutations generated with the CRISPR/Cas9 system in the AvrXa7/PthXo3 EBE of the *OsSWEET14* promoter exhibited enhanced *Xoo* resistance but did not show any significant difference in all examined agronomic traits compared to wild-type plants under net-house growth conditions. It is conceivable that limited modifications in promoter regions do not affect the normal expression of *SWEET* genes in contrast to KO or silenced lines. Our findings are consistent with the previous work of Oliva et al. [11] who studied 30 combinations of EBE mutations in the *OsSWEET11*, *OsSWEET13* and *OsSWEET14* promoters of the IR64 or Ciherang-Sub1 varieties and detected only a single line with abnormal agronomic traits.

Some individual *Xoo* strains have evolved a set of distinct TALE effectors that collectively target several members of the clade III SWEET family. The presence of these redundant TALEs thereby trumps single "~~loss~~ loss-~~of~~ of-~~tale~~ tale-responsiveness" resistance alleles [11,12,17,41]. For example, Kitaake lines carrying TALEN-induced mutation in the *SWEET14* promoter [13,15] exhibit resistance to strains which depend exclusively on matching AvrXa7/PthXo3 for clade III *SWEET* family induction. Likewise, the natural *xa13* allele [42] or CRISPR/Cas9-induced mutation in the *SWEET11* promoter [11] exhibit resistance to strains such as PXO99 which depend exclusively on PthXo1, for virulence. However, the BLB resistance of the Kitaake lines harboring mutations in both AvrXa7/PthXo3 (*OsSWEET14*) and PthXo1 (*OsSWEET11*) EBEs ~~were~~ was defeated by *Xoo* strains expressing simultaneously the AvrXa7/PthXo3

Field Code Changed

Formatted: Not Superscript/ Subscript

Formatted: Not Superscript/ Subscript

Field Code Changed

and PthXo2B TALEs [11]. Recently, the stacking of EBE-edited alleles in several *OsSWEET* promoters have overcome this limitation and was shown to achieve a broad spectrum of resistance to strains from most BLB-prone countries in Asia [11,12].

All of the three T_2 lines tested for BLB resistance were affected ~~only~~ for the AvrXa7/PthXo3 EBE and conserved an otherwise wild type TalF EBE (Fig 1C). The homozygous mutant TBR225 line L-5.7(-6) carrying a 6-bp deletion in the AvrXa7/PthXo3 EBE exhibited a significantly enhanced resistance to two Vietnamese *Xoo* strains compared to WT TBR225. The L-15.4(+1) and L-31.12(-3) lines that harbored more subtle alterations in the 3'-end of this EBE (a 1-bp insertion and a 3-bp deletion, respectively) in contrast remained susceptible to VXO strains. Our *OsSWEET14* expression analysis after Vietnamese Xoo strains inoculation (Fig 1C) suggests ~~It is therefore possible~~ that these editing events did not alter the EBE sequence sufficiently to compromise promoter recognition by an AvrXa7/PthXo3-like Vietnamese TALE. With less than 2 cm average lesion length, the resistance of line L-5.7(-6) (6-bp deletion) to the VXO_11 strain is rather extreme (versus average lesion length of 20.1 cm on wild type plants). Moreover, in this line, *OsSWEET14* expression following bacterial inoculation is strongly reduced relative the parental line and the two other edited lines, which suggest that in this case, recognition by an AvrXa7/PthXo3-like Vietnamese TALE is abrogated. Consistent with ~~this interpretation~~*OsSWEET14* expression analysis and as shown in S4 Fig, the Talvez [43] target prediction scores for AvrXa7 and PthXo3 on the *OsSWEET14* promoter L-5-a1 allele sequence of line L-5.7(-6) are markedly lower than on the wild

Formatted: Font: Italic, Not Superscript/ Subscript

Formatted: Font: Italic, Not Superscript/ Subscript

Formatted: Font: Italic, Not Superscript/ Subscript

Field Code Changed

Formatted: Not Superscript/ Subscript

type promoter sequence. This is not the case however for the edited alleles carried by lines L-15.4(+1) and L-31.12(-3) (respectively L-15 and L-31 in S4 Fig) whose Talvez scores are identical or slightly lower than those of the wild type promoter sequence.

The magnitude of the effect of the 6-bp deletion allele on susceptibility to VXO_11 is comparable to the dramatic effect of previously characterized alterations of the same EBEs in the Kitaake background against the PXO86 strain that possesses a single TALE, AvrXa7, targeting *OsSWEET14* for clade III *OsSWEET* gene induction [15]. By analogy, this suggests that *OsSWEET14* is also the only clade III *OsSWEETs* target of VXO_11 in the TBR225 background but, in order to confirm this hypothesis an examination of other clade III *OsSWEET*~~*s*~~ genes expression~~ induction~~ patterns in response to this strain would be required. The situation with the VXO_15 strain is not as straightforward to interpret and will require further investigations. Although the 6-bp deletion in the AvrXa7/PthXo3 EBE did provide an increased resistance to the edited plants, the VXO_15 strain caused intermediate disease severity ~~was still intermediate against the VXO_15 strain~~ (7.3 cm average lesion length on Fig 3). This incomplete resistance is unlikely to ~~could~~ result from the partial but still productive recognition of subsequences of the altered EBE by a VXO_15 AvrXa7/PthXo3-like TALE because *OsSWEET14* expression is similarly decreased in response to either this strain or VXO_11 (Fig 3C). Alternatively, ~~analogous to other Asian strains, VXO_15 may encode additional alternative TALEs, such as PthXo2B or PthXo1 that compensate the putative loss of~~ ~~*OsSWEET14*~~ ~~induction by targeting other clade III~~ ~~*OsSWEET*~~ ~~genes. C~~contrary to all Asian *Xoo* examined so far,

Formatted: Font: Italic, Not Superscript/ Subscript

(and thus less likely) but similar to African *Xoo* [15], VXO_15 may have the intrinsic potential to cause disease in the absence of clade III *OsSWEET* gene induction. More likely, analogous to other Asian strains, VXO_15 may encode alternative TALEs, such as PthXo2B or PthXo1 that compensate the loss of *OsSWEET14* induction by targeting other clade III *OsSWEET* genes. In this regard, long read genome sequencing will ultimately help describe These results nonetheless suggest that there are at least two groups within Vietnamese *Xoo* in terms of TALEs diversityvariability in Vietnamese *Xoo* strains.

In conclusion, we showed that editing specific EBEs of *Xoo* TALEs via CRISPR/Cas9 tool is an efficient method for improving BLB resistance of elite rice varieties such as TBR225 without detectable yield penalties. This also uncovered the potential diversity of TALEs in Vietnamese *Xoo* population, which will thus require future investigations to address the TALE repertoires of Vietnamese *Xoo* strains in order to generate broad-spectrum BLB-resistant rice varieties in Vietnam.

Authors' Contributions

Nguyen Duy Phuong and Tran Lan Dai are equal contributors

Conceptualization: Pham Xuan Hoi, Sebastien Cunnac, Nguyen Duy Phuong

Data Curation: Nguyen Duy Phuong

Formal Analysis: Nguyen Duy Phuong, Tran Lan Dai**,** Sebastien Cunnac, Pham Xuan Hoi

Funding Acquisition: Nguyen Duy Phuong, Pham Xuan Hoi, Tran Manh Bao

Investigation: Nguyen Duy Phuong, Tran Lan Dai, Pham Thu Hang, Phung Thi Thu Huong, Nguyen Thanh Ha, Pham Phuong Ngoc, Florence Auguy, Sebastien Cunnac

Methodology: Nguyen Duy Phuong**,** Tran Lan Dai, Pham Thu Hang, Sebastien Cunnac, Nguyen Thanh Ha, Pham Xuan Hoi

Project Administration: Pham Xuan Hoi

Resources: Pham Thu Hang, Florence Auguy, Nguyen Thanh Ha

Supervision: Sebastien Cunnac, Pham Xuan Hoi

Validation: Sebastien Cunnac, Pham Xuan Hoi

Writing – Original Draft Preparation: Nguyen Duy Phuong, Tran Lan Dai

Writing – review & editing: Sebastien Cunnac, Pham Xuan Hoi, Tran Manh Bao, Bui Thi Thu Huong

Acknowledgments

We are grateful to Msc. Pham Thi Van, Dr. Cao Le Quyen and Dr. Nguyen Van Cuu from the Institute of Agricultural Genetics for rice transformation experiments, Msc.

Nguyen Thi Thu Ha from the Institute of Agricultural Genetics for managing the *Xoo* strains collection and Msc. Nguyen Thi Nhung from Thaibinh Seeds Cor. for kindly providing the rice accessions.

References

1. Zhang H, Wang S. Rice versus *Xanthomonas oryzae* pv. *oryzae*: A unique pathosystem. Curr Opin Plant Biol. 2013; 16(2):188-195. doi:10.1016/j.pbi.2013.02.008 PMID: 23466254Zhang H, Wang S. Rice versus Xanthomonas oryzae pv. oryzae: A unique pathosystem. *Curr Opin Plant Biol*. 2013;16(2):188-195. doi:10.1016/j.pbi.2013.02.008

2. White FF, Potnis N, Jones JB, Koebnik R. The type III effectors of *Xanthomonas*. Mol Plant Pathol. 2009; 10(6):749-766. doi:10.1111/j.1364-3703.2009.00590.x PMID: 19849782

3. Boch J, Bonas U, Lahaye T. TAL effectors - pathogen strategies and plant resistance engineering. New Phytol. 2014; 204(4):823-832. doi:10.1111/nph.13015 PMID: 25539004

4. Bogdanove AJ, Schornack S, Lahaye T. TAL effectors: Finding plant genes for disease and defense. Curr Opin Plant Biol. 2010; 13(4):394-401. doi:10.1016/j.pbi.2010.04.010 PMID: 20570209

5. Bezrutczyk M, Yang J, Eom JS, Prior M, Sosso D, Hartwig T, Szurek B, Oliva R, Vera-Cruz C, White FF, Yang B, Frommer WB. Sugar flux and signaling in plant–microbe interactions. Plant J. 2018; 93(4):675-685. doi:10.1111/tpj.13775 PMID: 29160592

Formatted: Justified

6. Yang B, Sugio A, White FF. *Os8N3* is a host disease-susceptibility gene for bacterial blight of rice. Proc Natl Acad Sci U S A. 2006; 103(27):10503-10508. doi:10.1073/pnas.0604088103 PMID: 16798873

7. Antony G, Zhou J, Huang S, Li T, Liu B, White F, Yang B. Rice *xa13* recessive resistance to bacterial blight is defeated by induction of the disease susceptibility gene *Os-11N3*. Plant Cell. 2010; 22(11):3864-3876. doi:10.1105/tpc.110.078964 PMID: 21098734

8. Yu Y, Streubel J, Balzergue S, Champion A, Boch J, Koebnik R, Feng J, Verdier V, Szurek B. Colonization of rice leaf blades by an African strain of X*anthomonas oryzae* pv. *oryzae* depends on a new TAL effector that induces the rice nodulin-3 *Os11N3* gene. Mol Plant-Microbe Interact. 2011; 24(9):1102-1113. doi:10.1094/MPMI-11-10-0254 PMID: 21679014.

9. Streubel J, Pesce C, Hutin M, Koebnik R, Boch J, Szurek B. Five phylogenetically close rice *SWEET* genes confer TAL effector-mediated susceptibility to *Xanthomonas oryzae* pv. *oryzae*. New Phytol. 2013; 200(3):808-819. doi:10.1111/nph.12411 PMID: 23879865

10. Zhou J, Peng Z, Long J, Sosso D, Liu B, Eom JS, Huang S, Liu S, Vera Cruz C, Frommer WB, White FF, Yang B. Gene targeting by the TAL effector PthXo2 reveals cryptic resistance gene for bacterial blight of rice. Plant J. 2015; 82(4):632-643. doi:10.1111/tpj.12838 PMID: 25824104

11. Oliva R, Ji C, Atienza-Grande G, Huguet-Tapia JC, Perez-Quintero A, Li T, Eom JS, Li C, Nguyen H, Liu B, Auguy F, Sciallano C, Luu VT, Dossa GS, Cunnac S, Schmidt SM, Slamet-Loedin IH, Vera Cruz C, Szurek B, Frommer WB, White FF, Yang B. Broad-spectrum resistance to bacterial blight in rice using genome editing. Nat Biotechnol. 2019; 37(11):1344-1350. doi:10.1038/s41587-019-0267-z PMID: 31659337

12. Xu Z, Xu X, Gong Q, Li Z, Li Y, Wang S, Yang Y, Ma W, Liu L, Zhu B, Zou L, Chen G. Engineering broad-spectrum bacterial blight resistance by simultaneously disrupting variable TALE-binding elements of multiple susceptibility genes in rice. Mol Plant. 2019; 12(11):1434-1446. doi:10.1016/j.molp.2019.08.006 PMID: 31493565

13. Li T, Liu B, Spalding MH, Weeks DP, Yang B. High-efficiency TALEN-based gene editing produces disease-resistant rice. Nat Biotechnol. 2012; 30(5):390-392. doi:10.1038/nbt.2199 PMID: 22565958

14. Hutin M, Pérez-Quintero AL, Lopez C, Szurek B. MorTAL Kombat: The story of defense against TAL effectors through loss-of-susceptibility. Front Plant Sci. 2015; 6:535. doi:10.3389/fpls.2015.00535 PMID: 26236326

15. Blanvillain-Baufumé S, Reschke M, Solé M, Auguy F, Doucoure H, Szurek B, Meynard D, Portefaix M, Cunnac S, Guiderdoni E, Boch J, Koebnik R. Targeted promoter editing for rice resistance to *Xanthomonas oryzae* pv. *oryzae* reveals differential activities for *SWEET14*-inducing TAL effectors. Plant Biotechnol J.

2017; 15(3):306-317. doi:10.1111/pbi.12613 PMID: 27539813

16. Zaka A, Grande G, Coronejo T, Quibod IL, Chen CW, Chang SJ, Szurek B, Arif M, Cruz CV, Oliva R. Natural variations in the promoter of *OsSWEET13* and *OsSWEET14* expand the range of resistance against *Xanthomonas oryzae* pv. *oryzae*. PLoS One. 2018; 13(9):e0203711. doi:10.1371/journal.pone.0203711 PMID: 30212546

17. Huang S, Antony G, Li T, Liu B, Obasa K, Yang B, White FF. The broadly effective recessive resistance gene *xa5* of rice is a virulence effector-dependent quantitative trait for bacterial blight. Plant J. 2016; 86(2):186-194. doi:10.1111/tpj.13164 PMID: 26991395

18. Li J, Li Y, Ma L. CRISPR/Cas9-based genome editing and its applications for functional genomic analyses in plants. Small Methods. 2019; 3(3):1800473. doi:10.1002/smtd.201800473 PMID: 27895652

19. Van Hop D, Phuong Hoa PT, Quang ND, Ton PH, Ha TH, Van Hung N, et al. Biological control of *Xanthomonas oryzae* pv. *oryzae* causing rice bacterial blight disease by Streptomyces toxytricini VN08-A-12, isolated from soil and leaf-litter samples in Vietnam. Biocontrol Sci. 2014; 19(3):103-111. doi:10.4265/bio.19.103 PMID: 25252641

20. Furuya N, Taura S, Goto T, Trong Thuy B, Huu Ton P, Tsuchiya K, Yoshimura A. Diversity in virulence of *Xanthomonas oryzae* pv. *oryzae* from Northern Vietnam.

Japan Agric Res Q. 2012; 46(4):329-338. doi:10.6090/jarq.46.329

21. Nguyen HT, Vu QH, Van Mai T, Nguyen TT, Vu LD, Nguyen TT, Nguyen LV, Vu HTT, Nong HT, Dinh TN, Toshitsugu N, Vu LV. Marker-Assisted Selection of *Xa21* conferring resistance to bacterial leaf blight in indica rice cultivar LT2. Rice Sci. 2018; 25(1):52-56. doi:10.1016/j.rsci.2017.08.004

22. Jiang N, Yan J, Liang Y, Shi Y, He Z, Wu Y, Zeng Q, Liu X, Peng J. Resistance genes and their interactions with bacterial blight/leaf streak pathogens (*Xanthomonas oryzae*) in rice (*Oryza sativa* L.) - an updated review. Rice. 2020; 13(1):3. doi:10.1186/s12284-019-0358-y PMID: 31915945

23. Eom JS, Luo D, Atienza-Grande G, Yang J, Ji C, Thi Luu V, Huguet-Tapia JC, Char SN, Liu B, Nguyen H, Schmidt SM, Szurek B, Vera Cruz C, White FF, Oliva R, Yang B, Frommer WB. Diagnostic kit for rice blight resistance. Nat Biotechnol. 2019; 37(11):1372-1379. doi:10.1038/s41587-019-0268-y PMID: 31659338

24. Manh Bao T, Thi Hop T, Thi Tiec T, Thi Nhung N, Van Hoan N. Results of breeding TBR225 rice cultivar. Vietnam J. Agri. Sci. 2016; 14(9):1360-1367. In Vietnamese. Available from: http://www1.vnua.edu.vn/tapchi/Upload/15112016-so%208.7.pdf

25. Zhou J. Host target genes of the *Xanthomonas oryzae* pv . *oryzae* type III effectors for bacterial blight in rice. Graduate Theses and Dissertations, The Iowa State University. 2015. Available from:

https://lib.dr.iastate.edu/cgi/viewcontent.cgi?article=5476&context=etd

26. Li T, Huang S, Zhou J, Yang B. Designer TAL effectors induce disease susceptibility and resistance to *Xanthomonas oryzae* pv. *oryzae* in rice. Mol Plant. 2013; 6(3):781-789. doi:10.1093/mp/sst034 PMID: 23430045

27. Herbert L, Meunier AC, Bes M, Vernet A, Portefaix M, Durandet F, Michel R, Chaine C, This P, Guiderdoni E, Périn C. Beyond seek and destroy: how to generate allelic series using genome editing tools. Rice. 2020; 13(1):5. doi:10.1186/s12284-020-0366-y PMID: 31993780

28. Zhou H, Liu B, Weeks DP, Spalding MH, Yang B. Large chromosomal deletions and heritable small genetic changes induced by CRISPR/Cas9 in rice. Nucleic Acids Res. 2014; 42(17):10903-10914. doi:10.1093/nar/gku806 PMID: 25200087

29. Hiei Y, Ohta S, Komari T, Kumashiro T. Efficient transformation of rice (*Oryza sativa* L.) mediated by *Agrobacterium* and sequence analysis of the boundaries of the T- DNA. Plant J. 1994; 6(2):271-282. doi:10.1046/j.1365-313X.1994.6020271.x PMID: 7920717

30. Bai J, Choi SH, Ponciano G, Leung H, Leach JE. *Xanthomonas oryzae* pv. *oryzae* avirulence genes contribute differently and specifically to pathogen aggressiveness. Mol Plant Microbe Interact. 2000; 13(12):1322-1329. doi:10.1094/MPMI.2000.13.12.1322 PMID: 11106024

31. Ma X, Chen L, Zhu Q, Chen Y, Liu YG. Rapid decoding of sequence-specific

Formatted: French (France)

nuclease-induced heterozygous and biallelic mutations by direct sequencing of PCR products. Mol Plant. 2015; 8(8):1285-1287. doi:10.1016/j.molp.2015.02.012 PMID: 25747846

32. Razzaq A, Saleem F, Kanwal M, Mustafa G, Yousaf S, Imran Arshad HM, Hameed MK, Khan MS, Joyia FA. Modern trends in plant genome editing: An inclusive review of the CRISPR/Cas9 Toolbox. Int J Mol Sci. 2019; 20(16):4045. doi:10.3390/ijms20164045 PMID: 31430902

33. Wang F, Wang C, Liu P, Lei C, Hao W, Gao Y, Liu YG, Zhao K. Enhanced rice blast resistance by CRISPR/Cas9-Targeted mutagenesis of the ERF transcription factor gene *OsERF922*. PLoS One. 2016;11(4):e0154027. doi:10.1371/journal.pone.0154027 PMID: 27116122

34. Zhou H, He M, Li J, Chen L, Huang Z, Zheng S, Zhu L, Ni E, Jiang D, Zhao B, Zhuang C. Development of commercial thermo-sensitive genic male sterile rice accelerates hybrid rice breeding using the CRISPR/Cas9-mediated TMS5 editing system. Sci Rep. 2016; 6:37395. doi:10.1038/srep37395 PMID: 27874087

35. Zhang A, Liu Y, Wang F, Li T, Chen Z, Kong D, Bi J, Zhang F, Luo X, Wang J, Tang J, Yu X, Liu G, Luo L. Enhanced rice salinity tolerance via CRISPR/Cas9-targeted mutagenesis of the *OsRR22* gene. Mol Breed. 2019; 39:47. doi:10.1007/s11032-019-0954-y PMID: 32803201

36. Zafar K, Khan MZ, Amin I, Mukhtar Z, Yasmin S, Arif M, Ejaz K, Mansoor S.

Precise CRISPR-Cas9 mediated genome editing in super Basmati rice for resistance against bacterial blight by targeting the major susceptibility gene. Front Plant Sci. 2020; 11:575. doi:10.3389/fpls.2020.00575 PMID: 32595655

37. Kim YA, Moon H, Park CJ. CRISPR/Cas9-targeted mutagenesis of *Os8N3* in rice to confer resistance to *Xanthomonas oryzae* pv. *oryzae*. Rice. 2019; 12(1):67. doi:10.1186/s12284-019-0325-7 PMID: 31446506~~White FF, Potnis N, Jones JB, Koebnik R. The type III effectors of Xanthomonas. *Mol Plant Pathol*. 2009;10(6):749-766. doi:10.1111/j.1364-3703.2009.00590.x~~

~~3. Boch J, Bonas U, Lahaye T. TAL effectors - pathogen strategies and plant resistance engineering. *New Phytol*. 2014;204(4):823-832. doi:10.1111/nph.13015~~

~~4. Bogdanove AJ, Schornack S, Lahaye T. TAL effectors: Finding plant genes for disease and defense. *Curr Opin Plant Biol*. 2010;13(4):394-401. doi:10.1016/j.pbi.2010.04.010~~

~~5. Bezrutczyk M, Yang J, Eom JS, et al. Sugar flux and signaling in plant–microbe interactions. *Plant J*. 2018;93(4):675-685. doi:10.1111/tpj.13775~~

~~6. Yang B, Sugio A, White FF. Os8N3 is a host disease-susceptibility gene for bacterial blight of rice. *Proc Natl Acad Sci U S A*. 2006;103(27):10503-10508. doi:10.1073/pnas.0604088103~~

~~7. Antony G, Zhou J, Huang S, et al. Rice xa13 recessive resistance to bacterial blight is defeated by induction of the disease susceptibility gene Os-11N3. *Plant Cell*.~~

2010;22(11):3864-3876. doi:10.1105/tpc.110.078964

8. Yu Y, Streubel J, Balzergue S, et al. Colonization of rice leaf blades by an African strain of xanthomonas oryzae pv. oryzae depends on a new TAL effector that induces the rice nodulin-3 Os11N3 gene. *Mol Plant-Microbe Interact*. 2011;24(9):1102-1113. doi:10.1094/MPMI-11-10-0254

9. Streubel J, Pesce C, Hutin M, Koebnik R, Boch J, Szurek B. Five phylogenetically close rice SWEET genes confer TAL effector-mediated susceptibility to Xanthomonas oryzae pv. oryzae. *New Phytol*. 2013;200(3):808-819. doi:10.1111/nph.12411

10. Zhou J, Peng Z, Long J, et al. Gene targeting by the TAL effector PthXo2 reveals cryptic resistance gene for bacterial blight of rice. *Plant J*. 2015;82(4):632-643. doi:10.1111/tpj.12838

11. Oliva R, Ji C, Atienza-Grande G, et al. Broad-spectrum resistance to bacterial blight in rice using genome editing. *Nat Biotechnol*. 2019;37(11):1344-1350. doi:10.1038/s41587-019-0267-z

12. Xu Z, Xu X, Gong Q, et al. Engineering Broad-Spectrum Bacterial Blight Resistance by Simultaneously Disrupting Variable TALE-Binding Elements of Multiple Susceptibility Genes in Rice. *Mol Plant*. 2019;12(11):1434-1446. doi:10.1016/j.molp.2019.08.006

13. Li T, Liu B, Spalding MH, Weeks DP, Yang B. High-efficiency TALEN-based

gene editing produces disease-resistant rice. *Nat Biotechnol*. 2012;30(5):390-392. doi:10.1038/nbt.2199

14. Hutin M, Pérez-Quintero AL, Lopez C, Szurek B. MorTAL Kombat: The story of defense against TAL effectors through loss-of-susceptibility. *Front Plant Sci*. 2015;6(JULY). doi:10.3389/fpls.2015.00535

15. Blanvillain-Baufumé S, Reschke M, Solé M, et al. Targeted promoter editing for rice resistance to *Xanthomonas oryzae* pv. *oryzae* reveals differential activities for *SWEET14* -inducing TAL effectors. *Plant Biotechnol J*. 2017;15(3):306-317. doi:10.1111/pbi.12613

16. Zaka A, Grande G, Coronejo T, et al. Natural variations in the promoter of OsSWEET13 and OsSWEET14 expand the range of resistance against Xanthomonas oryzae pv. Oryzae. *PLoS One*. 2018;13(9). doi:10.1371/journal.pone.0203711

17. Huang S, Antony G, Li T, et al. The broadly effective recessive resistance gene xa5 of rice is a virulence effector-dependent quantitative trait for bacterial blight. *Plant J*. 2016;86(2):186-194. doi:10.1111/tpj.13164

18. Li J, Li Y, Ma L. CRISPR/Cas9-Based Genome Editing and its Applications for Functional Genomic Analyses in Plants. *Small Methods*. 2019;3(3):1800473. doi:10.1002/smtd.201800473

19. Hop D Van, Hoa PTP, Quang ND, et al. Biological control of Xanthomonas oryzae

pv. oryzae causing rice bacterial blight disease by Streptomyces toxytricini VN08-A-12, isolated from soil and leaf-litter samples in Vietnam. *Biocontrol Sci*. 2014;19(3):103-111. doi:10.4265/bio.19.103

20. Furuya N, Taura S, Goto T, et al. Diversity in virulence of Xanthomonas oryzae pv. Oryzae from Northern Vietnam. *Japan Agric Res Q*. 2012;46(4):329-338. doi:10.6090/jarq.46.329

21. Nguyen HT, Vu QH, Van Mai T, et al. Marker-Assisted Selection of Xa21 Conferring Resistance to Bacterial Leaf Blight in indica Rice Cultivar LT2. *Rice Sci*. 2018;25(1):52-56. doi:10.1016/j.rsci.2017.08.004

22. Jiang N, Yan J, Liang Y, et al. Resistance Genes and their Interactions with Bacterial Blight/Leaf Streak Pathogens (Xanthomonas oryzae) in Rice (Oryza sativa L.) — an Updated Review. *Rice*. 2020;13(1). doi:10.1186/s12284-019-0358-y

23. Eom JS, Luo D, Atienza-Grande G, et al. Diagnostic kit for rice blight resistance. *Nat Biotechnol*. 2019;37(11):1372-1379. doi:10.1038/s41587-019-0268-y

24. Mạnh Báo T, Thị Hợp T, Thị Tiệc T, Thị Nhung N, Văn Hoan N. *KẾT QUẢ CHỌN TẠO GIỐNG LÚA TBR225*. Vol 14.; 2016. Accessed October 13, 2020. www.vnua.edu.vn

25. Zhou J, Yang B. Host target genes of the Xanthomonas oryzae pv . oryzae type III effectors for bacterial blight in rice. Published online 2015.

26. Li T, Huang S, Zhou J, Yang B. Designer TAL effectors induce disease

susceptibility and resistance to xanthomonas oryzae pv. oryzae in rice. *Mol Plant*. 2013;6(3):781-789. doi:10.1093/mp/sst034

27. Herbert L, Meunier AC, Bes M, et al. Beyond Seek and Destroy: how to Generate Allelic Series Using Genome Editing Tools. *Rice*. 2020;13(1). doi:10.1186/s12284-020-0366-y

28. Zhou H, Liu B, Weeks DP, Spalding MH, Yang B. Large chromosomal deletions and heritable small genetic changes induced by CRISPR/Cas9 in rice. *Nucleic Acids Res*. 2014;42(17):10903-10914. doi:10.1093/nar/gku806

29. Hiei Y, Ohta S, Komari T, Kumashiro T. Efficient transformation of rice (Oryza sativa L.) mediated by Agrobacterium and sequence analysis of the boundaries of the T- DNA. *Plant J*. 1994;6(2):271-282. doi:10.1046/j.1365-313X.1994.6020271.x

30. Bai J, Choi SH, Ponciano G, Leung H, Leach JE. Xanthomonas oryzae pv. oryzae avirulence genes contribute differently and specifically to pathogen aggressiveness. *Mol Plant-Microbe Interact*. 2000;13(12):1322-1329. doi:10.1094/MPMI.2000.13.12.1322

31. Ma X, Chen L, Zhu Q, Chen Y, Liu YG. Rapid Decoding of Sequence-Specific Nuclease-Induced Heterozygous and Biallelic Mutations by Direct Sequencing of PCR Products. *Mol Plant*. 2015;8(8):1285-1287. doi:10.1016/j.molp.2015.02.012

32. Razzaq A, Saleem F, Kanwal M, et al. Modern trends in plant genome editing: An

inclusive review of the CRISPR/Cas9 Toolbox. *Int J Mol Sci*. 2019;20(16). doi:10.3390/ijms20164045

33. Wang F, Wang C, Liu P, et al. Enhanced rice blast resistance by CRISPR/ Cas9-Targeted mutagenesis of the ERF transcription factor gene OsERF922 - Supplementary data. *PLoS One*. 2016;11(4). doi:10.1371/journal.pone.0154027

34. Zhou H, He M, Li J, et al. Development of Commercial Thermo-sensitive Genic Male Sterile Rice Accelerates Hybrid Rice Breeding Using the CRISPR/Cas9-mediated TMS5 Editing System. *Sci Rep*. 2016;6. doi:10.1038/srep37395

35. Zhang A, Liu Y, Wang F, et al. Enhanced rice salinity tolerance via CRISPR/Cas9-targeted mutagenesis of the OsRR22 gene. *Mol Breed*. 2019;39(3). doi:10.1007/s11032-019-0954-y

36. Zafar K, Khan MZ, Amin I, et al. Precise CRISPR-Cas9 Mediated Genome Editing in Super Basmati Rice for Resistance Against Bacterial Blight by Targeting the Major Susceptibility Gene. *Front Plant Sci*. 2020;11. doi:10.3389/fpls.2020.00575

37. Kim YA, Moon H, Park CJ. CRISPR/Cas9-targeted mutagenesis of Os8N3 in rice to confer resistance to Xanthomonas oryzae pv. oryzae. *Rice*. 2019;12(1). doi:10.1186/s12284-019-0325-7

38. Molla KA, Shih J, Yang Y. Single-nucleotide editing for zebra3 and wsl5 phenotypes in rice using CRISPR/Cas9-mediated adenine base editors. *aBIOTECH*. 2020;1(2):106-118. doi:10.1007/s42994-020-00018-x

39. Chen LQ. SWEET sugar transporters for phloem transport and pathogen nutrition. New Phytol. 2014; 201(4):1150-1155. doi:10.1111/nph.12445 PMID: 24649486~~Chen LQ. SWEET sugar transporters for phloem transport and pathogen nutrition. *New Phytol*. 2014;201(4):1150-1155. doi:10.1111/nph.12445~~

40. Yang J, Luo D, Yang B, Frommer WB, Eom JS. SWEET11 and 15 as key players in seed filling in rice. New Phytol. 2018; 218(2):604-615. doi:10.1111/nph.15004 PMID: 29393510~~Yang J, Luo D, Yang B, Frommer WB, Eom JS. SWEET11 and 15 as key players in seed filling in rice. *New Phytol*. 2018;218(2):604-615. doi:10.1111/nph.1500~~4

41. Doucouré H, Pérez-Quintero AL, Reshetnyak G, Tekete C, Auguy F, Thomas E, Koebnik R, Szurek B, Koita O, Verdier V, Cunnac S. Functional and genome sequence-driven characterization of *tal* effector gene repertoires reveals novel variants with altered specificities in closely related malian *Xanthomonas oryzae* pv. *oryzae* strains. Front Microbiol. 2018; 9:1657. doi:10.3389/fmicb.2018.01657 PMID: 30127769~~Doucouré H, Pérez-Quintero AL, Reshetnyak G, et al. Functional and genome sequence-driven characterization of tal effector gene repertoires reveals novel variants with altered specificities in closely related malian Xanthomonas oryzae pv. oryzae strains. *Front Microbiol*. 2018;9(AUG). doi:10.3389/fmicb.2018.01657~~

42. Chu Z, Yuan M, Yao J, Ge X, Yuan B, Xu C, Li X, Fu B, Li Z, Bennetzen JL, Zhang Q, Wang S. Promoter mutations of an essential gene for pollen development

result in disease resistance in rice. Genes Dev. 2006; 20(10):1250-1255. doi:10.1101/gad.1416306 PMID: 16648463Chu Z, Yuan M, Yao J, et al. Promoter mutations of an essential gene for pollen development result in disease resistance in rice. *Genes Dev*. 2006;20(10):1250-1255. doi:10.1101/gad.1416306

43. Pérez-Quintero AL, Rodriguez-R LM, Dereeper A, López C, Koebnik R, Szurek B, Cunnac Set al. An Improved Method for TAL Effectors DNA-Binding Sites Prediction Reveals Functional Convergence in TAL Repertoires of Xanthomonas oryzae Strains. PLoS One. 2013; 8(7):68464. doi:10.1371/journal.pone.0068464 PMID: 23869221

Formatted: Font: Not Italic, Not Superscript/ Subscript

Supporting information

S1_raw_images. Original photograph used in Fig 2 for the RT-PCR gels panel.

S2_raw_images. Original photograph used in Fig 3C for the RT-PCR gels panel.

S1 Fig. Nucleotide sequence of the *OsSEET14* promoter in TBR225.

S2 Fig. Virulence of Vietnamese *Xoo* strains VXO_11 and VXO_15 on TBR225 rice. Grey points correspond to individual lesion length measurements while the black points indicate the calculated average value. The line range represents standard deviation.

S3 Fig. Picture of an individual plant from the homozygous mutant rice lines L-5.7(-6).

S4 Fig. Talvez scoring of AvrXa7, PthXo3 and TalF target EBES in the edited *OsSWEET14* promoter allele sequences. Score values are represented both by the length of the ~~a horizontal~~horizontal -bar and a fill color scale. Higher Talvez prediction scores reflect a better match between a predicted EBE and the sequence of RVD of the query TALE.

~~S5 Fig. S6~~5 Fig. ~~DNA~~Amplicon sequencing ~~analysis~~ of predicted off-target sites for the *OsSWEET14* promoter-sgRNA in annotated exons of the TBR225 edited line L-5.7(-6). Potential unintended target sequences including the PAM ~~Off-target sequences~~ are ~~were showed~~highlighted -in ~~the~~ boxes. They are all identical to the expected wild type Nipponbare sequences.

Formatted: Font color: Auto, Not Superscript/ Subscript

Formatted: Font: Italic, Font color: Auto, Not Superscript/ Subscript

Formatted: Font: Not Bold, Not Superscript/ Subscript

Formatted: Font: Not Bold, Font color: Auto, Not Superscript/ Subscript

S1 Table. Key figures on the TBR225 transformation procedure for *OsSWEET14* promoter editing.

S2 Table. Output of the CCTop tool used with the *OsSWEET14* promoter sgRNA for off-target prediction on the rice Nipponbare genome.~~**Off-target sequences of *OsSWEET14* sgRNA**~~

Formatted: Font: Not Bold

Formatted: Font: Bold, Not Superscript/ Subscript

Formatted: Font: Bold

Formatted: Font: Bold

Formatted: Font color: Auto

Formatted: Font: Bold

Formatted: Font: Italic, Font color: Auto

RESPONSE TO REVIEWERS

You will find below the itemized list of our replies to the comments made about our re-submission.

Review Comments to the Author

Reviewer #1

	Reviewer's Comments	Responses
1	Though there are still some discrepancies that could have improved the manuscript, authors have adequately justified those. For example, they assume that 6 base pair deletion in the promoter region can make a difference in TALE binding but not in gene expression or induction of expression. While working on a promoter editing, it is generally expected to check the expression pattern of the gene under normal and infested condition after editing. Since they did not find any difference in phenotype and it was probably difficult to conduct the experiment, they have avoided the experiment.<br>Authors claim in the conclusion that the study uncovered potential diversity of TALEs. Though the discussion made by authors indicate towards it, they have not done any experiment to verify that. Hence the statement must be modified accordingly.	As also described in our response to comment #3 of Reviewer 2, we have performed the experiment recommended by the reviewers and the results have been integrated in this version of the manuscript (Figure 3C and results section). We believe that this new data (loss of *OsSWEET14* expression in response to both VXO_11 and VXO_15) brings more indirect support to our hypothesis that VXO_15 encodes additional TALE(s) capable of targeting other *OsSWEET* genes. We modified the discussion to accommodate this new data and rephrased this section to attenuate the strength of our statement.
2	Though most of the grammatical errors have been rectified still there are some errors as mentioned below:<br>- Ln 33: 'All examined agronomic traits of three transgene-free T2 lines were not significantly different from those of wild-type TBR225' may read as 'None of the examined agronomic traits of three transgene-free T2 lines were significantly different from those of wild-type TBR225'<br>- Ln 67: recessive resistance? – I pointed	- This was revised as suggested by the Reviewer

	it earlier, but the explanation was more confusing and must be addressed<br>- Ln 381: The single nucleotide mutation was observed only in two of nine plants. So the type of mutation should be only insertion or deletion but not single nucleotide insertion or deletion	- This was revised as "resistance"<br><br>- This was corrected as suggested by the Reviewer

Reviewer #2

	Reviewer's Comments	Responses
1	1. The reply to my comment 3 is not satisfactory scientifically. If authors need to back their gene selection in a rational way, they should cite earlier paper that described Asian strains target *SWEET14* or *SWEET13*. For easy reference see below my comment and author's response-<br><br>My original comment 3: I am wondering if Vietnamese Xoo VXO_11 and VXO_15 strains are known to secrete AvrXa7/PthXo3 from any earlier studies. If not, then how the authors have selected *SWEET14* for expression analysis and then editing the EBEs? How the authors hypothesized that *SWEET14* is the probable target *S* gene for *Xoo* VXO_11 and VXO_15 strains?<br>Authors replied: As thoughtfully pointed out by the reviewer below, based on previous studies, we knew that Asian strains tend to target either *OsSWEET14* or *OsSWEET13*. So, we just tested OsSWEET14 induction and were very lucky it turned to be the good choice.	We apologize if we do not fully grasp the point of the reviewer. Candidate gene selection could in principle be entirely random and still yield scientifically sound and serendipitous results in the end. Nevertheless, we believe we provide a reasonable rationale for why focus on *OsSWEET14*:<br><br>- in the introduction, we comment on the specificity of *OsSWEET* gene targeting by Asian strains in the paragraph "Previous studies established that rice resistance to *Xoo* resulting from "TALE-unresponsive" alleles can be conferred by natural DNA polymorphisms […] BLB resistance engineering thus required multiplex *OsSWEET* promoters EBE editing using the CRISPR/Cas9 system [11,12]." and extensively cite the literature that previously addressed this issue.<br><br>- we begin the result section with this sentence "*OsSWEET14/Os11N3* was previously identified as a susceptibility gene for *Xoo* strains relying on either of the AvrXa7, PthXo3, TalF (formerly Tal5) or TalC TALEs for infection of the rice cultivars Nipponbare and Kitaake". We modified the following sentence to say that because it is often targeted by Xoo strains, we focused our initial work on this promoter.
2	Figure caption: S4: Explain a bit more about TALVEZ scoring for making it easy for the	We added a sentence in the Figure legend briefly explaining the nature of the Talvez score and making it

	readers	clear that it increases with the likeliness of a candidate EBE being a genuine target based on the RVD-nucleotide association code.
3	Similarly, reply to my original comment 6 is not satisfactory. It is not understood why authors are reluctant to perform expression analysis. This experiment needs to be done, otherwise the manuscript looks like a substandard one.<br><br>Original comment 6: For another line of confirmation, expression analysis of SWEET14 gene from the edited lines (before/after infection) would be a great addition. Authors discussed “This incomplete resistance could result from the partial but still productive recognition of subsequences of the altered EBE by a VXO_15 AvrXa7/PthXo3-like TALE.” This could be simply analysed by expression analysis in the edited line.<br><br>Authors replied: We agree that examining OsSWEET14 expression in the edited lines would help decide between possible explanations for the partial resistance phenotype against<br>VX0_15. As described in our reply to Reviewer 1's comment #1, we however believe this is beyond the scope of the core results of our study. To tackle this issue, we are in the process of generating the resources to obtain a good vision of the tal genes content of some VXO strains (including VXO_11 and VXO_15). This and the suggested expression assays will be part of a follow up study focusing on the mechanisms explaining these phenotypes	At the reviewers' request, we have performed additional experiments and have included this data in a new panel of Figure 3. The corresponding *OsSWEET14* expression data in the parent and edited lines following Vietnamese strains inoculation is consistent with the interpretation that only the most dramatic 6bp deletion in the AvrXa7 EBE abrogates *OsSWEET14* upregulation and causes resistance to the examined Vietnamese Xoo strains.

4	Authors have not performed off-target analysis even for revised manuscript. Which is a standard practice for performing CRISPR-Cas9 experiment. Authors used a single guide and analyzing off-targets for a single guide is an easy task	At the reviewer's request, we have performed off-target sites predictions with the sequence of the gRNA used for editing and selected three likely potential unintended target sites located in annotated exons for PCR amplicon sequencing with genomic DNA from the resistant L-5.7(-6) line. This analysis did not detect genome modifications and the results were included in the new version of the manuscript (Table S2, Figure S5, results section).
5	Authors have not taken care of the following original comment in their discussion in the revised manuscript. Line number 382-390 in the revised manuscript.<br>Original comment: other 5: Line 347-349: See the discussion of an earlier publication (https://doi.org/10.1007/s42994-020-00018-x). The authors may cite and take help from the paper to discuss additional mutations in T1 generation.<br>In the Page 116 of the suggested paper, it is discussed "Plants descendent from mutants generated by active Cas9 are prone to further rounds of editing until the PAM and seed region of protospacer are destroyed by editing." Please also discuss your result in this line	We have added this reference to the list of papers we previously cited in this section of the discussion and have revised our sentence to convey the notion suggested by the reviewer.
