# Supplementary material for: Improved bacterial leaf blight disease resistance in the major elite Vietnamese rice cultivar TBR225 via editing of the OsSWEET14 promoter
Source: PLoS One. 2021 Sep 9;16(9):e0255470. doi: 10.1371/journal.pone.0255470 (PMC8428762; doi:10.1371/journal.pone.0255470)
Supplement: Attachment — Submitted filename: Response to Reviewers.docx [file pone.0255470.s010.docx]

**Response to reviewers**

You will find below the itemized list of our replies to the comments made about our original submission. We would like to thank the appointed reviewers for their time and efforts assessing our work. The feedback we received was extremely helpful for improving the manuscript and we hope we did a satisfactory job at addressing the various points raised during the reviewing process.

**Journal requirements**

| **Requirements** | **Responses** |
| --- | --- |
| 1. Please ensure that your manuscript meets PLOS ONE's style requirements, including those for file naming. | The manuscript was revised to meet PLOS ONE's style |
| 2. We suggest you thoroughly copyedit your manuscript for language usage, spelling, and grammar. If you do not know anyone who can help you do this, you may wish to consider employing a professional scientific editing service. | The manuscript was thoroughly checked and revised. |
| 3. At this time, please address the following queries:     a. Please clarify the sources of funding (financial or material support) for your study. List the grants or organizations that supported your study, including funding received from your institution. b. State what role the funders took in the study. If the funders had no role in your study, please state: “The funders had no role in study design, data collection and analysis, decision to publish, or preparation of the manuscript.” c. If any authors received a salary from any of your funders, please state which authors and which funders. d. If you did not receive any funding for this study, please state: “The authors received no specific funding for this work.”     Please include your amended statements within your cover letter; we will change the online submission form on your behalf. | The amended statements were included in the cover letter |
| 4. PLOS ONE now requires that authors provide the original uncropped and unadjusted images underlying all blot or gel results reported in a submission’s figures or Supporting Information files. This policy and the journal’s other requirements for blot/gel reporting and figure preparation are described in detail at [https://journals.plos.org/plosone/s/figures#loc-blot-and-gel-reporting-requirements](https://journals.plos.org/plosone/s/figures#_blank) and [https://journals.plos.org/plosone/s/figures#loc-preparing-figures-from-image-files](https://journals.plos.org/plosone/s/figures#_blank). When you submit your revised manuscript, please ensure that your figures adhere fully to these guidelines and provide the original underlying images for all blot or gel data reported in your submission. See the following link for instructions on providing the original image data: [https://journals.plos.org/plosone/s/figures#loc-original-images-for-blots-and-gels](https://journals.plos.org/plosone/s/figures#_blank). | The original uncropped and unadjusted image underlying the reported gel results was provided as the "S1_raw_images" supporting information file. |

**Review Comments to the Author**

**Reviewer #1**

|  | **Reviewer’s Comments** | **Responses** |
| --- | --- | --- |
| 1 | Authors have reported a deletion of six-bases in the promoter region of OsSWEET14 gene that resulted in mutants, which were resistant to one of the Xoo strains tested. However, they were unable to verify what is the minimum number of nucleotide deletion required to get high level resistance. If it is possible for authors to do further experiments to verify this, it will strengthen the manuscript. | In our mind, the main and most important merit of our study is to report on the production of a genome edited Vietnamese elite variety line resistant to BLB caused by Xoo strains originating from this country which has practical implications for the design of domestic BLB control strategies.  We fully agree with the reviewer that understanding why other edited alleles, with less pronounced deletions failed to confer resistance is a very interesting question. Clearly, further experiments examining *OsSWEET14* induction in these lines may provide conclusive evidence regarding this question. We definitely have started and will continue to address this and a number of related questions insightfully asked by both reviewers because, first, they are academically fundamental issues, but also, because they impinge on our ability to design broad resistance adapted to the diversity of Vietnamese strains. But as emphasized on several occasions below, we feel that although this would unquestionably strengthen the anticipated impact of the work, which is not a criteria for publication into PLOS One, it requires an elaborate set of integrated experiments (gene expression studies, bacterial genome sequencing to elucidate actual tal sequences , …) that is beyond the scope of the present work.  As an alternative way to start addressing this question and the question #6 of reviewer 2, we have used the TALE target DNA prediction software Talvez to compare the predicted scores of the AvrXa7, PthXo3 and TalF TALEs on the complete set of our edited promoter allele sequences. This result has been included in S4 Fig and commented in the discussion |
| 2 | In many cases, the authors have mentioned the experimental method in the results section and those should be moved back to materials and methods section. For example, Ln 228-232 can be moved to material and methods section. | We revised the entire results section with this comment in mind and hope we fixed the most blatant cases like we did for example for former lines 228-232. |
| 3 | Ln 36: The plants have been numbered as x.y.z. Commonly, plants are numbered as x-y-z format where x, y and z represent the offspring number of the plant in subsequent generations. I strongly suggest renaming the plants for easy understanding and to follow the convention. | The plants were renamed as “L-x” for T_0_ plants, “L-x.y” for T_1_ plants.  The progenies of T_1_ plants were numbered using the format L-x.y(mutation type). |
| 4 | Ln 159: cite appropriate reference for selecting OsEF1α as reference gene for RT-PCR | The reference “Blanvillain-Baufumé S, Reschke M, Solé M, Auguy F, Doucoure H, Szurek B, et al. Targeted promoter editing for rice resistance to *Xanthomonas oryzae* pv. *oryzae* reveals differential activities for *SWEET14*-inducing TAL effectors. Plant Biotechnol J. 2017; 15(3):306-317. doi:10.1111/pbi.12613 PMID: 27539813” was added in. |
| 5 | Ln 303: Significant difference is clearly visible but no asterisks in Fig. 3B | This was a mistake. Asterisks were added in Fig. 3B |
| 6 | Ln 319: use lower-case or upper-case letters for all means that don’t vary significantly. | This was revised |
| 7 | Ln 325-327: Gives an impression that only three studies have been reported for rice improvement, which is not true. Please modify the statement! | We have revised this paragraph in order to avoid giving this misleading impression and hope that it now more adequately conveys the idea that rice improvement with genome editing, while growing in number and scope, is still in early stages. |
| 8 | Ln 367: Authors assume that limited modifications in promoter regions might not have affected the normal expression of SWEET genes in contrast to KO or silenced lines but they must prove it by using RT-PCR or QRT-PCR before the manuscript can be accepted for publication. | The statement pointed out in the reviewer's comment is part of a section of the discussion whose main purpose is to make a case about the conceivable merits of precise editing relative to gross gene inactivation with regards to minimizing the unintentional impact of genome modifications on the physiology of the edited plants in an agronomic context.  We agree that this is still a conjecture but it seems plausible considering that we and several other groups have not detected significant alterations in grow and reproductive traits of similar editing events whereas clade III OsSWEET-silenced or knock out lines were reported to have developmental defects. In order to unquestionably demonstrate that the *OsSWEET14* promoter edition events have no impact on gene expression, one would need to monitor its expression in various tissues, organs, developmental stages and growth conditions. Other research groups have indeed embarked on such a labor intensive endeavor in an attempt to also better characterize the function of this gene in processes unrelated to BLB susceptibility which is still an area where knowledge is very limited at best. We therefore believe that even if interesting from an academic stand point, the requested experiments go beyond the primary scope of our study. |
| 9 | Ln 393: Authors claim that probably OsSWEET14 is the only S gene target of tested Xoo strain VXO_11 without testing other SWEET genes. The lesion observed is very less but that is not the enough evidence support this statement. Either they must test the EBEs related to other genes or should remove this statement. | We have revised this section of the discussion in order to cite previous studies that are consistent with our hypothesis and to moderate the strength of the claim made by this statement. We hope this will satisfactorily address the reviewer's point while at the same time allow us to still mention this open issue of interest for the discussion. |
| 10 | Additionally, there are a lot of typos in the manuscript. I have mentioned few of those below, but the manuscript needs to be verified thoroughly for such grammatical errors. |  |
|  | Ln 60: ration should read as ratio | We deliberately used the word ['ration'](https://dictionary.cambridge.org/dictionary/english/ration) |
|  | Ln 67: recessive – delete? | Here is “recessive” |
|  | Ln 72: obtained? | Revised as “obtained” |
|  | Ln 82: World should read as world | Revised as “world” |
|  | Ln 97: conferred/ confer? | Revised as “confer” |
|  | Ln 97: Is this new identification or verification? | This point was clarified |
|  | Ln 220: OsSWEET14 2 day post-infiltration can be written as OsSWEET14 two day post-infiltration | Revised as “OsSWEET14 two day post-infiltration” |
|  | Ln 384: very broad spectrum can be written as broad spectrum | Revised as “broad spectrum” |

**Reviewer #2**

|  | **Reviewer’s Comments** | **Responses** |
| --- | --- | --- |
| 1 | Overall, the work is interesting, but the manuscript is poorly written with numerous grammatical mistakes/typos/sentence construction errors | We have made every efforts to address these flaws and the manuscript has been edited and revised to address the most conspicuous problems, hopefully in a satisfactorily fashion. |
| 2 | The authors have a similar paper published in Vietnamese (<http://www.tapchikhoahocnongnghiep.vn/uploads/news/2019_01/7_1.pdf>) . I could understand authors have designed 3 guide RNAs in that published report. The full paper is not accessible for a detail verification. Authors need to clarify what additional work they have done in this study. | In the previous paper (pdf file provided with our revisions), we focused on gRNA design methods and bioinformatics tools. *OsSWEET14* was used as an example. This previous study reports on the results of identifying and screening target gRNAs with different bioinformatics tools: CRISPR MultiTargeter for the identification of gRNAs, Genetyx 4.0. for the identification of GC%, Mfold 2.3 for the prediction of sgRNA structure, CCTop for the prediction of off-target sites. Ultimately, 3 gRNAs, targeting the TalC or Tal5 EBEs located on the promoter or Exon I of the *OsSWEET14* gene, were tentatively designed. The published study did not entail vector construction or plant transformation experiment.  Here, a different gRNA targeting AvrXa7/PthXo3/TalF EBEs was designed by using a combination of CRISPR-P v2.0 and CCTop tools. |
| 3 | Abstract: “In this study, we proved that the expression of TBR225 OsSWEET14 was induced by the infection of two representative Vietnamese Xoo VXO_11 and VXO_15 strains”. This claim is not supported by the results obtained in the study. Authors have studied the expression of only one member (SWEET14) of SWEET gene family. The data is not sufficient to claim that the two strains induce only SWEET14. Additional SWEET genes need to be included to get a complete picture. | The abstract has been substantially revised and this statement has been reworded to " two representative Vietnamese Xoo strains were shown to up-regulate OsSWEET14 upon TBR225 infection" which in our view does not claim that the VXO strains 'exclusively' induce OsSWEET14 but just that they induce it. |
| 4 | I am wondering if Vietnamese Xoo VXO_11 and VXO_15 strains are known to secrete AvrXa7/PthXo3 from any earlier studies. If not, then how the authors have selected SWEET14 for expression analysis and then editing the EBEs? How the authors hypothesized that SWEET14 is the probable target S gene for Xoo VXO_11 and VXO_15 strains? | As thoughtfully pointed out by the reviewer below, based on previous studies, we knew that Asian strains tend to target either *OsSWEET14* or *OsSWEET13*. So we just tested *OsSWEET14* induction and were very lucky it turned to be the good choice. |
| 5 | After the initial expression analysis, authors have set out to edit the EBE sequence in the SWEET14 promoter. There are 4 different known EBEs (TalC, TalF, AvrXa7, and PthXo3) located in the promoter. The guide RNAs designed in the study makes a double strand break in the overlapping EBEs for AvrXa7 and PthXo3. Why the authors preferred to target this EBE over TalC and TalF is not clear from the introduction section. Briefly mention that TalC and TalF are mainly present in African lineages. From the analysis of Oliva et al (2019), “Asian strains had approximately equal numbers of PthXo2 (targeting SWEET13) and PthXo3/AvrXa7 (SWEET14)”. This again raises question, why authors choose to go for only Sweet14? | As a follow up of the previous reply and again as precisely pointed out by the reviewer, indeed, there are 4 EBEs that have been identified in the *OsSWEET14* promoter. Because TalC is currently exclusively found in African strains, we reasoned that it was unlikely that Vietnamese strains carried a copy of it. We therefore designed the gRNA in order to maximize our chances to perturb all remaining TalF, AvrXa7, and PthXo3 EBEs that are overlapping to some extent. We have modified the beginning of the "CRISPR/Cas9 design for OsSWEET14 promoter editing" results section to explain this point. |
| 6 | From Figure 1C, it is understood that in some lines (e.g. L1.27), deleted bases comprise of both AvrXa7 and TalF EBEs. It would be interesting to know their disease reactions compared to the lines with only AvrXa7 disrupted. | Thank you for this insightful comment. In reaction to this, we revised the multiple alignment of edited alleles sequences (Figure 1C) that did not accurately reflect the potential for unchanged binding sites (TalF EBE) for some of the alleles. We also added a short statement in connection to this topic in the discussion.  Regarding those three lines that have been analyzed further: L-5.7(-6), L-31.12(-3) and L-15.4(+1). All three are predicted to still be sensitive to TalF because its EBE is intact.  From the complete list of lines in Table 2 we are still in the process of analyzing the segregation and propagating seeds for additional events and we are currently unable to test transgene-free homozygous lines. If extrapolating, based on analogous alleles obtained previously by Blanvillain-Baufumé *et al*. on Kitaake, some of the alleles in Figure 1C (e.g. L4, L7, L27, L29) have indeed a high chance to be insensitive to TalF because the first (T) and/or second 5'-nucleotide of the EBE is/are absent. However, even if these were indeed unresponsive to TalF, we believe it is highly unlikely that they would be resistant to African Xoo strains because these always carry also TalC and because, we and others, have observed that African strains in contrast to their Asian counter parts are able to maintain virulence in the absence of clade III SWEET induction.  Finally, even if we agree that this will definitely need to be tested, we predict that those alleles will not provide resistance to Vietnamese strains because in our view the lesions in the AvrXa7/PthXo3 EBE are not dramatic enough to prevent recognition by their cognate TALE.  Finally, these statements are substantiated by the new S4 Fig mentioned in our reply to Reviewer's 1 comment #1. |
| 7 | For another line of confirmation, expression analysis of SWEET14 gene from the edited lines (before/after infection) would be a great addition. Authors discussed “This incomplete resistance could result from the partial but still productive recognition of subsequences of the altered EBE by a VXO_15 AvrXa7/PthXo3-like TALE.” This could be simply analysed by expression analysis in the edited line. | We agree that examining *OsSWEET14* expression in the edited lines would help decide between possible explanations for the partial resistance phenotype against VX0_15. As described in our reply to Reviewer 1's comment #1, we however believe this is beyond the scope of the core results of our study. To tackle this issue, we are in the process of generating the resources to obtain a good vision of the tal genes content of some VXO strains (including VXO_11 and VXO_15). This and the suggested expression assays will be part of a follow up study focusing on the mechanisms explaining these phenotypes. |
| 8 | 7. Line 1.5.7 had 6 bp deletion and showed the best resistance response. Please add the sequence details in Figure 1C. | The T_2_ line 1.5.7 (now referred to as L-5.7(-6)) was generated from the T_1_ line 1.5; and its *OsSWEET14* promoter sequence corresponds to the 6bp deletion allele referenced to as "L-5-a1" in Fig 1C. We have made this explicit in the revised version of the "TBR225 OsSWEET14 promoter editing confers resistance to Vietnamese Xoo" paragraph and the reader can directly consult Fig 1C for the sequence. |
| 9 | L1.5.7 line was resistant to VXO_11 but moderately to the VXO_15 strain. This indicates VXO_15 might possess additional TALE and induces distinct SWEET. Authors have discussed this in line 399-403. This demands additional experiments, at least analyzing expression of SWEET genes. | Please see our reply to comment #7. |
| 10 | What is the percentage of editing in T0 generation? Please provide a table. | The percentage of editing in T0 generation was present in the original manuscript (line 235). Following the reviewer’s comment, we added the S1 Table as Supporting information with key figures about the transformation procedure. |
| 11 | Have the authors analyzed off-target editing? If so, please mention here. | No, we did not. |
| 12 | Line no 368…. Authors said …. “our findings are consistent with the previous work with Oliva et al 2019 ----------- multiple combinations of EBE mutations in the OsSWEET11, OsSWEET13 and OsSWEET14 promoters and did not observe abnormal agronomic traits in Kitaake rice”. This information is not fully correct. Genome edited rice line IR64-106 showed phenotypic differences regarding yield, panicle length and fertility. Consider mentioning it. | We have revised our statement. |
| 13 | Please provide an image of genome edited TBR225 mutant rice line with its wild type counterpart to show Phenotypic similarity. | This image was added in “Supporting informations” under name “S3 Fig” |
| 14 | Abstract: 1. Please check if ‘Viet Nam’ or ‘Vietnam’ is correct | Revised as “Vietnam” |
|  | 2. “OsSWEET14 belongs …. host S gene”. Please split into two sentences to make it easy to follow. | This sentence was split into two sentences. |
|  | 3. “Using CRISPR/Cas9 gene editing system, nine TBR225 mutant lines targeting the AvrXa7/PthXo3 effector binding element (EBE) located on OsSWEET14 promoter region were identified from ten transgenic plants.” Replace ‘targeting’ with “with disrupted AvrXa7/PthXo3……” | Revised as reviewer’s comment |
|  | 4. Line 31-32: “Genotyping analysis of T0 and T1 showed that all mutations were stably inherited to the offspring next generation” Follow the suggestion. | Revised as reviewer’s comment |
| 15 | Introduction: Line 51-53: “TALEs are injected into the plant cell, bind to specific nuclear host gene promoter sequences termed Effector-Binding Elements (EBEs) and induce target gene expression to the benefit of the pathogen”. Modify—Once TALEs are …..cell, they bind to……..” | Revised as reviewer’s comment |
| 16 | Line 58-59: “All Xoo strains recurrently target S genes belonging to the SWEET gene family and coding for transmembrane sugar exporter proteins”. Needs proper citation. | Reference was additionally cited. |
| 17 | Material and Methods: ‘Gene expression analysis’ should go earlier than gRNA design. | This was rearranged |
| 18 | Explain the SWEET once in the beginning. | Explanation was added in Abstract |
| 19 | Why SWEET14 is not clear? Please briefly mention what makes author to go for SWEET14 promoter editing | To keep the abstract short enough, we mentioned that together with other clade III SWEET family members, OsSWEET14 is a BLB susceptibility gene. |
| 20 | Figure legends and tables are misplaced. Please rectify | Figure legends and tables were placed following the guidelines of the Journal |
| 21 | Page 6: gRNA design: I see there are more than 3 distinct guide options to disrupt the EBE (for PthXo3 and AvrXa7). Why the authors have selected the one depicted in Figure 1a need to be briefly explained. | Explanations were provided in the "CRISPR/Cas9 design for *OsSWEET14* promoter editing" paragraph of the results section. |
| 22 | Line 347-349: See the discussion of an earlier publication (<https://doi.org/10.1007/s42994-020-00018-x>). The authors may cite and take help from the paper to discuss additional mutations in T1 generation. | Thanks you for the pointer to this interesting paper. |
| 23 | Figure 1c, replace ‘Tal5’ with TalF to make it consistent with the figure legend. | Revised as reviewer’s comment |
| 24 | Line 60: Correct the carbohydrate spelling | Revised as reviewer’s comment |
| 25 | Line 107. ……the sentence --in Hanoi-Vietnam in 2013 and 2016, respectively). Please omit first bracket ‘)’ at the end of the sentence. | Revised as reviewer’s comment |
| 26 | Line 114…. Please change ‘base on’ to ‘based on’. | Revised as reviewer’s comment |
| 27 | Line 125. ‘The obtained vector was the integrity of the inserted fragment was verified by sequencing’. Rectify it like- ‘The clone was verified by sequencing’. | This was revised as “The integrity of the inserted fragment was verified by sequencing” |
| 28 | Line 142. Explain PSA media | Explanation was added in |
| 29 | Line 351. Please correct. genome, , omit extra comma. | Revised as reviewer’s comment |
| 30 | Line 384. In the sentence ‘to achieve very broad spectrum of resistance’.. Please delete the word very before broad spectrum. | Revised as reviewer’s comment |
| 31 | fig.2B. replace the figure labelling OsSWEET to OsSWEET14 | This was replaced |
| 32 | Line 249: correct analyzis | This was corrected |
| 33 | Line 372-385: very poorly written with poor sentence construction. Make them simpler and grammatically correct. | We have revised this paragraph. |
| 34 | Line 249: “All T1 individuals derived from each of two T0 putative homozygotes..” the highlighted portion is not understood. | This was revised as “All T_1_ individuals derived from each T_0_ putatively homozygous line” |
| 35 | Line 268: “with 88.9% of the T0 lines generating T-DNA-free”. How this 88.9% has been calculated? | The T-DNA-free ratio was calculated with the following formula: (no. of T0 lines generating T-DNA-free T1 plants / no. of T0 edited lines) x 100%.  Here, “no. of T0 lines generating T-DNA-free T1 plants” was “8”; and “no. of T0 edited lines” was “9”. |
| 36 | Line 328: ‘Northern of Vietnam’ correct it | This was corrected |
|  | Line 111: replace Prime with Primer | This was replaced |
