# Supplementary material for: Improved bacterial leaf blight disease resistance in the major elite Vietnamese rice cultivar TBR225 via editing of the OsSWEET14 promoter
Source: PLoS One. 2021 Sep 9;16(9):e0255470. doi: 10.1371/journal.pone.0255470 (PMC8428762; doi:10.1371/journal.pone.0255470)
Supplement: Attachment — Submitted filename: Response to reviewers.docx [file pone.0255470.s011.docx]

**Response to reviewers**

You will find below the itemized list of our replies to the comments made about our re-submission.

**Review Comments to the Author**

**Reviewer #1**

|  | **Reviewer’s Comments** | **Responses** |
| --- | --- | --- |
| 1 | Though there are still some discrepancies that could have improved the manuscript, authors have adequately justified those. For example, they assume that 6 base pair deletion in the promoter region can make a difference in TALE binding but not in gene expression or induction of expression. While working on a promoter editing, it is generally expected to check the expression pattern of the gene under normal and infested condition after editing. Since they did not find any difference in phenotype and it was probably difficult to conduct the experiment, they have avoided the experiment. Authors claim in the conclusion that the study uncovered potential diversity of TALEs. Though the discussion made by authors indicate towards it, they have not done any experiment to verify that. Hence the statement must be modified accordingly. | As also described in our response to comment #3 of Reviewer 2, we have performed the experiment recommended by the reviewers and the results have been integrated in this version of the manuscript (Figure 3C and results section). We believe that this new data (loss of *OsSWEET14* expression in response to both VXO_11 and VXO_15) brings more indirect support to our hypothesis that VXO_15 encodes additional TALE(s) capable of targeting other *OsSWEET* genes. We modified the discussion to accommodate this new data and rephrased this section to attenuate the strength of our statement. |
| 2 | Though most of the grammatical errors have been rectified still there are some errors as mentioned below:   - Ln 33: 'All examined agronomic traits of three transgene-free T2 lines were not significantly different from those of wild-type TBR225' may read as 'None of the examined agronomic traits of three transgene-free T2 lines were significantly different from those of wild-type TBR225' - Ln 67: recessive resistance? – I pointed it earlier, but the explanation was more confusing and must be addressed - Ln 381: The single nucleotide mutation was observed only in two of nine plants. So the type of mutation should be only insertion or deletion but not single nucleotide insertion or deletion | - This was revised as suggested by the Reviewer - This was revised as “resistance” - This was corrected as suggested by the Reviewer |

**Reviewer #2**

|  | **Reviewer’s Comments** | **Responses** |
| --- | --- | --- |
| 1 | 1. The reply to my comment 3 is not satisfactory scientifically. If authors need to back their gene selection in a rational way, they should cite earlier paper that described Asian strains target *SWEET14* or *SWEET13*. For easy reference see below my comment and author’s response-  My original comment 3: I am wondering if Vietnamese Xoo VXO_11 and VXO_15 strains are known to secrete AvrXa7/PthXo3 from any earlier studies. If not, then how the authors have selected *SWEET14* for expression analysis and then editing the EBEs? How the authors hypothesized that *SWEET14* is the probable target *S* gene for *Xoo* VXO_11 and VXO_15 strains? Authors replied: As thoughtfully pointed out by the reviewer below, based on previous studies, we knew that Asian strains tend to target either *OsSWEET14* or *OsSWEET13*. So, we just tested OsSWEET14 induction and were very lucky it turned to be the good choice. | We apologize if we do not fully grasp the point of the reviewer. Candidate gene selection could in principle be entirely random and still yield scientifically sound and serendipitous results in the end. Nevertheless, we believe we provide a reasonable rationale for why focus on *OsSWEET14*:  - in the introduction, we comment on the specificity of *OsSWEET* gene targeting by Asian strains in the paragraph "Previous studies established that rice resistance to *Xoo* resulting from "TALE-unresponsive" alleles can be conferred by natural DNA polymorphisms […] BLB resistance engineering thus required multiplex *OsSWEET* promoters EBE editing using the CRISPR/Cas9 system [11,12]." and extensively cite the literature that previously addressed this issue.  - we begin the result section with this sentence "*OsSWEET14/Os11N3* was previously identified as a susceptibility gene for *Xoo* strains relying on either of the AvrXa7, PthXo3, TalF (formerly Tal5) or TalC TALEs for infection of the rice cultivars Nipponbare and Kitaake". We modified the following sentence to say that because it is often targeted by Xoo strains, we focused our initial work on this promoter. |
| 2 | Figure caption: S4: Explain a bit more about TALVEZ scoring for making it easy for the readers | We added a sentence in the Figure legend briefly explaining the nature of the Talvez score and making it clear that it increases with the likeliness of a candidate EBE being a genuine target based on the RVD-nucleotide association code. |
| 3 | Similarly, reply to my original comment 6 is not satisfactory. It is not understood why authors are reluctant to perform expression analysis. This experiment needs to be done, otherwise the manuscript looks like a substandard one.  Original comment 6: For another line of confirmation, expression analysis of SWEET14 gene from the edited lines (before/after infection) would be a great addition. Authors discussed “This incomplete resistance could result from the partial but still productive recognition of subsequences of the altered EBE by a VXO_15 AvrXa7/PthXo3-like TALE.” This could be simply analysed by expression analysis in the edited line.  Authors replied: We agree that examining OsSWEET14 expression in the edited lines would help decide between possible explanations for the partial resistance phenotype against VX0_15. As described in our reply to Reviewer 1's comment #1, we however believe this is beyond the scope of the core results of our study. To tackle this issue, we are in the process of generating the resources to obtain a good vision of the tal genes content of some VXO strains (including VXO_11 and VXO_15). This and the suggested expression assays will be part of a follow up study focusing on the mechanisms explaining these phenotypes | At the reviewers' request, we have performed additional experiments and have included this data in a new panel of Figure 3. The corresponding *OsSWEET14* expression data in the parent and edited lines following Vietnamese strains inoculation is consistent with the interpretation that only the most dramatic 6bp deletion in the AvrXa7 EBE abrogates *OsSWEET14* upregulation and causes resistance to the examined Vietnamese Xoo strains. |
| 4 | Authors have not performed off-target analysis even for revised manuscript. Which is a standard practice for performing CRISPR-Cas9 experiment. Authors used a single guide and analyzing off-targets for a single guide is an easy task | At the reviewer's request, we have performed off-target sites predictions with the sequence of the gRNA used for editing and selected three likely potential unintended target sites located in annotated exons for PCR amplicon sequencing with genomic DNA from the resistant L-5.7(-6) line. This analysis did not detect genome modifications and the results were included in the new version of the manuscript (Table S2, Figure S5, results section). |
| 5 | Authors have not taken care of the following original comment in their discussion in the revised manuscript. Line number 382-390 in the revised manuscript. Original comment: other 5: Line 347-349: See the discussion of an earlier publication (<https://doi.org/10.1007/s42994-020-00018-x>). The authors may cite and take help from the paper to discuss additional mutations in T1 generation. In the Page 116 of the suggested paper, it is discussed “Plants descendent from mutants generated by active Cas9 are prone to further rounds of editing until the PAM and seed region of protospacer are destroyed by editing.” Please also discuss your result in this line | We have added this reference to the list of papers we previously cited in this section of the discussion and have revised our sentence to convey the notion suggested by the reviewer. |
