# Supplementary material for: Improved bacterial leaf blight disease resistance in the major elite Vietnamese rice cultivar TBR225 via editing of the OsSWEET14 promoter
Source: PLoS One. 2021 Sep 9;16(9):e0255470. doi: 10.1371/journal.pone.0255470 (PMC8428762; doi:10.1371/journal.pone.0255470)
Supplement: Attachment — Submitted filename: Review Comments_June 14 2021.docx [file pone.0255470.s012.docx]

Comments on the manuscript entitles, “Improved bacterial leaf blight disease resistance in the major elite Vietnamese rice cultivar TBR225 via editing of the OsSWEET14 promoter” (PONE-D-20-33201R2)

Although this manuscript is a significantly improved one over its earlier version, the following points need to be addressed before publication. Please find my specific major comments below. Moreover, there are many minor points that require correction and modification. Minor points can be found in the attached annotated PDF file of the manuscript.

1. Why only Sweet 14 expression has been analysed and why authors targeted Sweet 14 and not the others need an acceptable justification in the introduction or discussion section.
2. Fig 2B: I am wondering about the level of normal expression of SWEET14 in H2O treated rice samples. Even the authors have mentioned in discussion that normal expression of SWEET genes are necessary for plant development. “It is conceivable that limited modifications in promoter regions do not affect the normal expression of *SWEET* genes in contrast to KO or silenced lines.”

Authors may have a look at Fig 2B in an earlier publication: [10.1111/pbi.12613](https://dx.doi.org/10.1111%2Fpbi.12613), where H2O treated sample showed expression of SWEET14. Why it is different in the current manuscript? Please discuss.

3. Please cite and discuss the observation of A similar article recently published

“Zeng, X., Luo, Y., Vu, N.T.Q. *et al.* CRISPR/Cas9-mediated mutation of *OsSWEET14* in rice cv. Zhonghua11 confers resistance to *Xanthomonas oryzae* pv. *oryzae* without yield penalty. *BMC Plant Biol* **20,** 313 (2020). <https://doi.org/10.1186/s12870-020-02524-y>”
