# Supplementary material for: Improved bacterial leaf blight disease resistance in the major elite Vietnamese rice cultivar TBR225 via editing of the OsSWEET14 promoter
Source: PLoS One. 2021 Sep 9;16(9):e0255470. doi: 10.1371/journal.pone.0255470 (PMC8428762; doi:10.1371/journal.pone.0255470)
Supplement: Attachment — Submitted filename: Response to reviewers.docx [file pone.0255470.s014.docx]

**Response to reviewers**

You will find below the itemized list of our replies to the comments made about our re-submission.

**Review Comments to the Author**

**Reviewer #1**

|  | **Reviewer’s Comments** | **Responses** |
| --- | --- | --- |
| 1 | Ln 38-40: This conclusive statement of abstract does not match the title. This can be a supplementary statement but not the only statement. | We have modified the last part of the abstract to address this comment. |
| 2 | Ln 410-412: The authors have written “In contrast, here, all the T1 plants generated from both heterozygous lines L-21 and L-29, regardless of whether they had a CRISPR/Cas9 T-DNA transgene integrated in their genome, did not show any new mutation possibly because CRISPR/Cas9 T-DNA transgene was no longer functional.” There is no reason why the CRISPR tools will become inactive, and authors did no effort to test this. The statement must be removed since it can give a wrong message. | The corresponding statement has been deleted. |

**Reviewer #2**

|  | **Reviewer’s Comments** | **Responses** |
| --- | --- | --- |
| 1 | Why only Sweet 14 expression has been analysed and why authors targeted Sweet 14 and not the others need an acceptable justification in the introduction or discussion section | This comment is similar to earlier comments from Reviewer #2. We copy our previous reply :  "*we believe we provide a reasonable rationale for why focus on OsSWEET14:*  *- in the introduction, we comment on the specificity of OsSWEET gene targeting by Asian strains in the paragraph "Previous studies established that rice resistance to Xoo resulting from "TALE-unresponsive" alleles can be conferred by natural DNA polymorphisms […] BLB resistance engineering thus required multiplex OsSWEET promoters EBE editing using the CRISPR/Cas9 system [11,12]." and extensively cite the literature that previously addressed this issue.*  *- we begin the result section with this sentence "OsSWEET14/Os11N3 was previously identified as a susceptibility gene for Xoo strains relying on either of the AvrXa7, PthXo3, TalF (formerly Tal5) or TalC TALEs for infection of the rice cultivars Nipponbare and Kitaake". We modified the following sentence to say that because it is often targeted by Xoo strains, we focused our initial work on this promoter.*"  We honestly believe these explanations are "acceptable", simply because these are the actual principles that truthfully guided our decision making during this work.  We have further revised a section of the discussion "In this regard, deciphering clade III *OsSWEET* genes expression patterns in combination with long read genome sequencing will ultimately help describe TALEs variability in Vietnamese *Xoo* strains and its functional impact on *OsSWEET* genes induction." to highlight the fact that future work will examine induction of other clade III *SWEET* genes to understand the behavior of VXO_15. |
| 2 | Fig 2B: I am wondering about the level of normal expression of SWEET14 in H2O treated rice samples. Even the authors have mentioned in discussion that normal expression of SWEET genes are necessary for plant development. “It is conceivable that limited modifications in promoter regions do not affect the normal expression of *SWEET* genes in contrast to KO or silenced lines.”  Authors may have a look at Fig 2B in an earlier publication: [10.1111/pbi.12613](https://dx.doi.org/10.1111%2Fpbi.12613), where H2O treated sample showed expression of SWEET14. Why it is different in the current manuscript? Please discuss. | In our comment "“It is conceivable that limited modifications in promoter…" it is implicit that "normal expression of *SWEET* genes" is relevant not only in leaves but in any cell type or developmental step where this gene is expressed and where it has a functional role in the biological process.  Here, in order to compare the relative expression of *OsSWEET14* in inoculated wild type and edited lines, we used semi-quantitative RT-PCR (35 cycles). This technique has a much narrower dynamic range than the qRT-PCR used in the Blanvillain-Baufumé et al. paper where cycling is pushed up to 40 cycles and that uses a more sensitive fluorescent DNA detection method.  So, one likely simple explanation for the difference is that in our experimental settings, semi-quantitative RT-PCR lacks sensitivity relative to qRT-PCR and fails to display a band on the gel. Another explanation could be that different rice varieties were examined. Furthermore, in both studies, OsSWEET expression levels are measured in relative terms, so rigorous comparisons across studies are difficult.  Even if we cannot provide a definitive answer for the absolute *SWEET14* leaf basal expression levels in TBR225, based on our unpublished RNA-seq data, it appears that in Nipponbare, this gene is expressed at very low levels in H20 treated leaves, close to detection thresholds or not at all. |
| 3 | Please cite and discuss the observation of A similar article recently published  “Zeng, X., Luo, Y., Vu, N.T.Q. *et al.* CRISPR/Cas9-mediated mutation of *OsSWEET14* in rice cv. Zhonghua11 confers resistance to *Xanthomonas oryzae* pv. *oryzae* without yield penalty. *BMC Plant Biol* **20,** 313 (2020). <https://doi.org/10.1186/s12870-020-02524-y>” | Thank you for pointing this very interesting reference. We have mentioned it in the revised discussion. |
